# SuFEx‐Enabled Catalytic Synthesis of Fluorescent Organosulfur Polymers for the Rapid Detection of Explosives

**DOI:** 10.1002/advs.202506616

**Published:** 2025-07-11

**Authors:** Sun Bu Lee, Jaeyoung Heo, Seunghyun Noh, Tae Eun An, Gyeongsoo Kim, Gang Min Lee, Junggong Kim, Keunyoung Kim, Jongman Lee, Taeyeon Kim, Changsik Song, Han Yong Bae

**Affiliations:** ^1^ Department of Chemistry Sungkyunkwan University Suwon 16419 Republic of Korea; ^2^ PNL Global Co., Ltd. B1, 10 Sapyeong‐daero 2‐gil, Seocho‐gu Seoul 06652 Republic of Korea; ^3^ Present address: RIST Jeollanam‐do Gwangyang 57801 Republic of Korea

**Keywords:** explosives detection, fluorescence quenching, pentiptycene‐based polymers, SuFEx click chemistry, time‐resolved spectroscopy

## Abstract

The rapid and straightforward detection of explosive molecules is crucial for ensuring public safety and national security. Herein, a new series of conjugated polymers, referred to as the SP series, incorporating pentiptycene structures with organosulfur (sulfide and sulfonate ester) side chains, which is developed via sulfur(VI)‐fluoride exchange (SuFEx) click chemistry. These polymers enhance the detection capabilities of nitroaromatic explosives through improved fluorescence quenching mechanisms. The method involved synthesizing β‐sulfido sulfonyl fluorides via superbase‐catalyzed thia‐Michael reactions, introducing sulfonates through SuFEx conjugation, and assembling the polymers via palladium‐catalyzed Sonogashira coupling. The SP‐7 polymer, in particular, shows exceptional performance in explosives detection (DNT and TNT), achieving a >99% quenching efficiency within a few seconds, far surpassing existing polymer sensors. The fluorescence quenching dynamics via electron transfer in the SP‐7 polymer are thoroughly elucidated using time‐resolved photoluminescence and transient absorption spectroscopy, revealing that both static and dynamic quenching processes are highly effective. Remarkably, a straightforward yet powerful TLC‐spotting‐based technique is demonstrated that enables the rapid (<30 s) and selective detection of explosives under UV irradiation. This finding underscores the potential of SP series polymers in explosive practical detection, promising significant improvements in public security technologies.

## Graphical abstract

Rapid detection of explosives is essential for public safety and national security. Here, conjugated polymers (SPs), incorporating pentiptycene with organosulfur side chains, have been reported using sulfur(VI)‐fluoride exchange (SuFEx). The SP‐7 polymer exhibited exceptional fluorescence quenching (>99% in 60 s) for detecting nitroaromatic explosives. A simple and efficient TLC‐spotting method enabled selective explosive detection under UV irradiation, highlighting its practical applications in security technology.

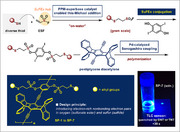

## 1 Introduction

**Figure 1:**
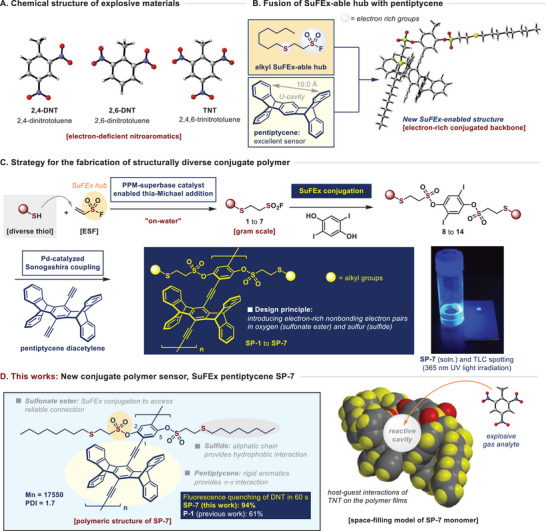
Concept of this work: SuFEx pentiptycene (SP) polymer for new explosive sensor.

Explosive detection technologies are essential for public safety and national security.^[^ ^1^ ^]^ Currently, nitroaromatic explosives such as 2,4,6‐trinitrotoluene (TNT) and 2,4‐ or 2,6‐dinitrotoluene (DNT) can be detected through fluorescence quenching^[^ ^2^ ^]^ mechanisms using conjugated polymer sensors,^[^ ^3^, ^4^ ^]^ which are known for their excellent sensitivity and efficacy (**Figure** **1**A). Particularly, the integration of pentiptycene receptors into polymer structures has significantly advanced the detection of nitroaromatics.^[^ ^5^ ^]^ Yang and Swager disclosed a seminal result regarding poly(*p*‐phenylene–ethynylenes) (PPEs)^[^ ^6^ ^]^ P‐1 which exhibits high sensitivity to TNT vapor, utilizing the amplification properties of conjugated polymers and the fluorescence quenching transduction method.^[^ ^7^ ^]^ This innovation highlighted the potential of conjugated polymer‐based sensors for efficient explosive detection:^[^ ^8^ ^]^ electron‐deficient analytes act as acceptors, and photo‐excited polymers^[^ ^9^ ^]^ function as donors. A critical feature of such polymeric sensor is rigid, 3D pentiptycene^[^ ^10^ ^]^ backbone. This design effectively inhibits the formation of excimers and π‐stacking, maintaining high fluorescence quantum yields and reducing interpolymer interactions.^[^ ^11^ ^]^ In addition, the appropriate solubility of the polymers in organic solvents and create cavities^[^ ^12^ ^]^ within the polymers for explosive molecules to penetrate the polymer‐coated film.^[^ ^13^ ^]^ Such structural advantage enhances the effectiveness of fluorescence quenching upon exposure to analyte gases.^[^ ^14^ ^]^ However, the demand for newly tunable structures persists, and a limitation of known materials is the incapability to modify the electron‐donating effect of the main backbone skeleton by systematically inventing the linker structure through facile chemical transformations to change the electronic environment, enabling the development of next‐generation sensors applicable to a wide range of potential analytes.

Sulfur(VI)‐fluoride exchange (SuFEx)^[^ ^15^, ^16^, ^17^, ^18^, ^19^ ^]^ is a novel click chemistry that facilitates the coupling between electrophilic sulfur molecules and nucleophilic alcohols or amines. Conceptualized by Sharpless et al. in 2014,^[^ ^19^ ^]^ SuFEx has since evolved during the last decade, expanding its conjugation applicability from small molecule to biopolymers.^[^ ^20^, ^21^, ^22^, ^23^ ^]^ Numerous contemporary catalytic studies have highlighted the benefits of reactions performed under “on‐water” environment.^[^ ^24^, ^25^ ^]^ A critical functional hub for these purposes is the sulfonyl fluoride group,^[^ ^26^, ^27^ ^]^ which offers significant advantages due to its high stability in water and resistance to specific catalytic conditions, thereby allowing the introduction of various catalytic reactions. We hypothesized that combining SuFEx‐relevant chemistry with a pentiptycene backbone could allow the design of conjugated polymers with exceptional electron‐deficient nitroaromatic sensing capabilities by introducing organosulfur linkages in various oxidation states, facilitated by multiple nonbonding electron pairs (Figure 1B). In this context, our research group demonstrated that β‐aryl sulfido sulfonyl fluorides could be efficiently synthesized via a high‐turnover catalytic thia‐Michael addition^[^ ^28^ ^]^ between arylated ethenesulfonyl fluorides (ESFs) and diverse thiols, enabled by an “on‐water” hydrophobic amplification effect.^[^ ^29^ ^]^ Subsequently, studies by the An group revealed that sulfonyl fluorides bearing a sulfur atom in the γ‐position can be activated for SuFEx click reaction through intramolecular chalcogen bonding.^[^ ^16^ ^]^ In this scaffold, the γ‐sulfur engages in a non‐covalent 1,5‐sulfur···fluorine interaction, which enhances the electrophilicity of the sulfonyl fluoride group and promotes nucleophilic attack. Inspired by those insights, we developed an efficient strategy for monomer synthesis by incorporating a similar γ‐sulfur motif via thia‐Michael addition to ethenesulfonyl fluoride. A more specific strategy involves i) synthesizing diverse β‐sulfido sulfonyl fluorides via catalytic thia‐Michael reactions, ii) easily introducing sulfonate esters through SuFEx conjugation, and iii) subsequently constructing conjugated polymers through Pd‐catalyzed Sonogashira coupling^[^ ^30^ ^]^ with pentiptycene acetylene (Figure 1C).

Herein, we report highly efficient explosive sensors of organosulfur side chains incorporating pentiptycene polymers through SuFEx‐empowered catalytic processes. Following our proposed strategy, we successfully developed SP series polymer sensors, referred to as SuFEx‐pentiptycene. These SP sensors exhibited exceptional chemical stability. Notably, the incorporation of sulfide side chains was efficiently achieved through a superbase organocatalysis on‐water on gram scales, underscoring their structural robustness. Subsequent SuFEx conjugation allowed for the facile introduction of sulfonate ester, and the Pd‐catalyzed Sonogashira coupling reactions enabled the straightforward synthesis of the target materials. Among these, the SP‐7 materials demonstrated outstanding performance in DNT detection,^[^ ^31^ ^]^ achieving a fluorescence quenching efficiency of 94% within 60 seconds. This result is significantly superior to the previously reported high‐performance reference polymer P1, which showed a quenching efficiency of 61%. Furthermore, the SP‐7 sensor also exhibited top‐tier performance in TNT sensing, matching the highest levels reported (Figure 1D).

## 2 Results and Discussion

### 2.1 Design and Synthesis of SP Series Polymer

**Figure 2:**
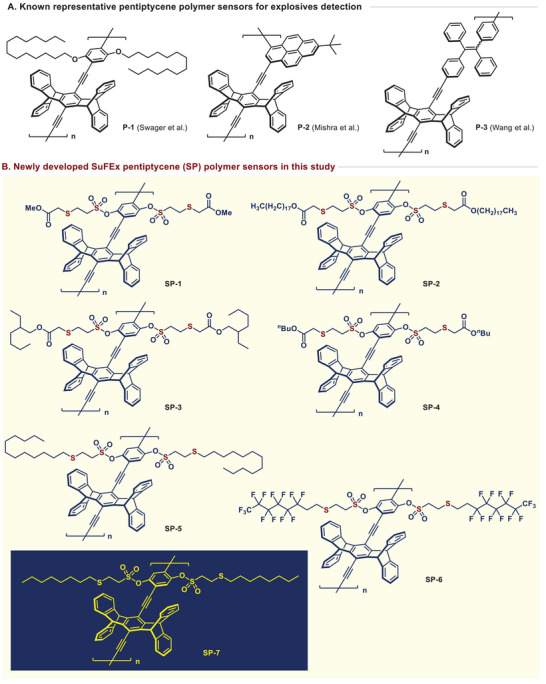
Chemical structures of known polymer sensors and SP polymer sensors.

Inspired by the structure of the pentiptycene polymer P‐1 reported by the Swager group, various laboratories have synthesized polymers with diverse structures and reported their performances. For instance, the Mishra group synthesized polymers containing pyrene linkers,^[^ ^32^ ^]^ while the Wang group reported the synthesis of structures conjugated with tetraphenylethylene (**Figure** **2**A).^[^ ^33^ ^]^ Drawing inspiration from our previous findings on organocatalytic reactions, we designed a range of polymers featuring electron‐rich sulfide and sulfonate ester linkages. Employing the strategy of SuFEx click chemistry, we were able to design variations of the pentiptycene structure, leading to the successful synthesis of seven novel polymers (Figure 2B).

**Figure 3:**
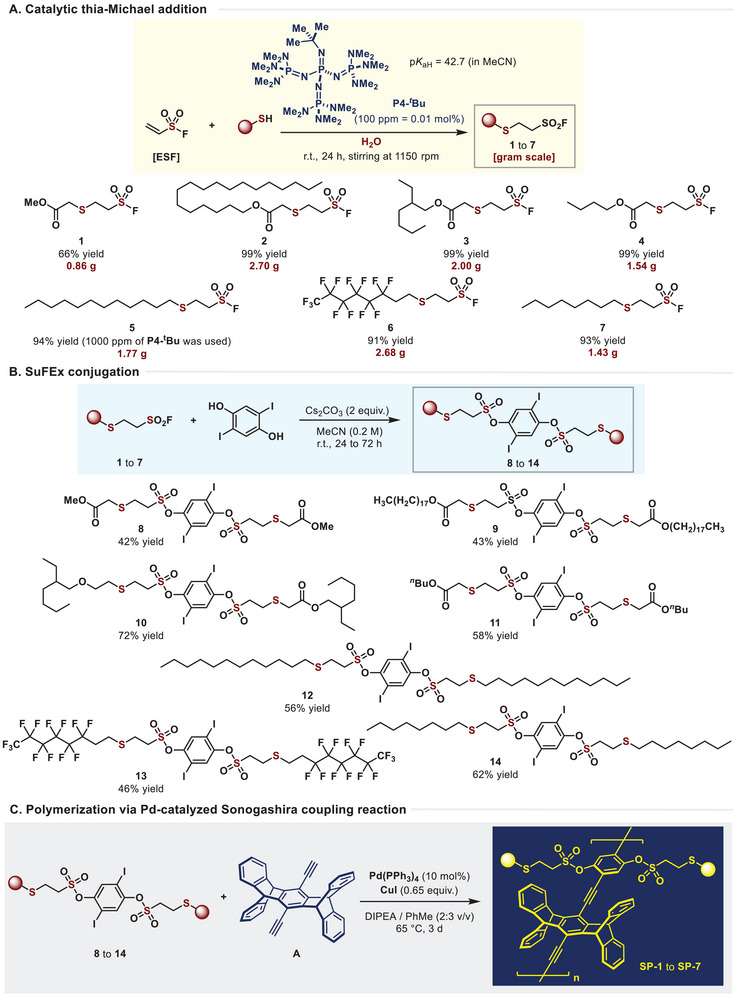
Synthetic procedures of SP series polymers.

The primary modular unit ESF,^[^ ^34^, ^35^ ^]^ has demonstrated significant potential in materials science and medicinal chemistry due to its ability to accommodate substantial substituents at the β‐position. Notably, β‐aryl substituted ESF has been exploited to operate efficiently in reactions mediated by phosphazene superbases,^[^ ^36^ ^]^ *N*‐heterocyclic carbene catalysts,^[^ ^37^ ^]^ and visible‐light photocatalysts.^[^ ^38^ ^]^ Recent catalytic conditions have elucidated that both ionic (involving electron pairs) and radical (involving single electron) mechanisms can effectively facilitate the desired on‐water reactions. Inspired by this fact, we have developed a reaction that directly introduces alkyl thiols into ESF, proceeding in the presence of a P4‐*^t^* Bu superbase catalyst. We successfully introduced seven types of functionalized aliphatic thiols through thia‐Michael addition, synthesizing materials with the intended sulfonyl fluoride structures (compounds **1**–**7**) in yields up to 99%, predominantly on a gram‐scale, forming sulfide bonds (**Figure** **3**A). The synthesized thia‐Michael adducts enables intramolecular chalcogen bonding, thereby increasing the efficiency of SuFEx click reaction.^[^ ^16^, ^29^ ^]^ Our recent studies have found that alkylsulfonyl fluoride effectively engages in SuFEx conjugation with phenols, forming sulfonate ester groups. Consequently, we anticipated facile coupling into disulfones in the presence of 2,5‐diiodobenzene‐1,4‐diol. To our delight, this reaction occurred without any degradation of the side chain structure, proceeding sulfone coupling at the para‐position of diols (Figure 3B, compounds **8**–**14**). Lastly, the functionalized diiodide intermediates were subjected to polymer synthesis under conditions of Sonogashira coupling, facilitated by tetrakis(triphenylphosphine)palladium(0) and copper(I) iodide catalysts. Remarkably, this coupling exhibited excellent reactivity, leading to the successful synthesis of seven novel polymers, SP‐1 to SP‐7, with excellent solubility in solvents (Figure 3C).

### 2.2 Analytical Data of Polymers

We measured the quantum yields (QY) and other key properties of newly synthesized polymers SP‐1 through SP‐7, along with the previously reported polymer P‐1, and summarized the results (**Table** **1**). SP‐7 displayed a QY of 74%, significantly surpassing the 43% of P‐1. Additionally, SP‐7 exhibited absorption and fluorescence emission maxima at 358 and 420 nm, respectively, indicating an optimal range for efficient excitation and emission for sensing applications. In fluorescence quenching sensors, a higher QY is crucial because even minimal light absorption can generate a strong fluorescence signal, which enhances both sensitivity and detection limits. This characteristic is particularly important for detecting trace amounts of nitroaromatic explosives. The combination of SP‐7′s high QY and favorable optical properties suggests superior fluorescence quenching performance, making it a strong candidate for practical explosive detection sensors.

**Table 1:** Chemical properties of prepared polymers (measured in CHCl_3_ solution).

Polymer	Molecular weight, *M*_n_ [PDI]	$\lambda_{\max}^{\mathrm{abs}}$ [nm]	$\lambda_{\max}^{\mathrm{fl}}$ [nm]	Quantum yield, ϕ_fl_
**P‐1**	48 251 (1.6)	434 nm	460 nm	43%
**SP‐1**	3803 (1.3)	350 nm	419 nm	39%
**SP‐2**	5167 (1.4)	356 nm	420 nm	18%
**SP‐3**	13 622 (2.1)	357 nm	418 nm	31%
**SP‐4**	5719 (1.4)	355 nm	417 nm	25%
**SP‐5**	16 967 (1.6)	357 nm	417 nm	67%
**SP‐6**	12 824 (1.8)	355 nm	424 nm	31%
**SP‐7**	17 550 (1.7)	358 nm	420 nm	**74%**

**Figure 4:**
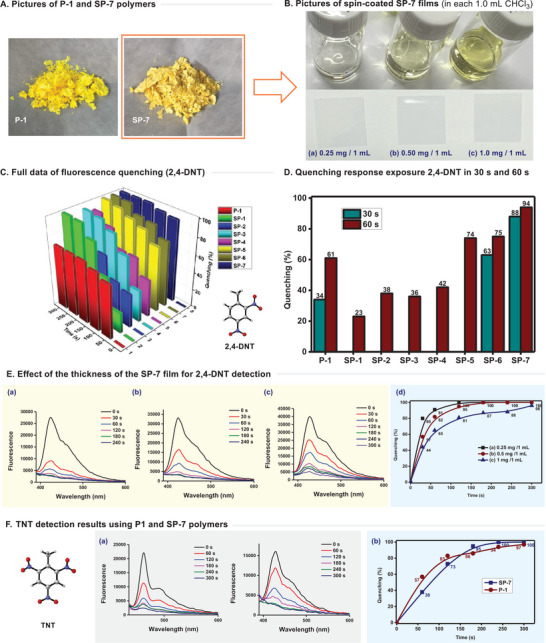
Fluorescence quenching results of SP series polymers using 2,4‐DNT and TNT vapors.

Compared to the known polymer P‐1, our new polymer SP‐7 was observed to have a slightly deeper yellow color (**Figure** **4**A). SP‐7 exhibited excellent solubility in CHCl_3_ solvent, facilitating the simple fabrication of films through spin coating (Figure 4B: 0.25, 0.5, and 1.0 mg mL^−1^). The fluorescence quenching of the polymer was assessed using 2,4‐DNT in the gas phase. The effectiveness of fluorescence quenching for all synthesized polymers against 2,4‐DNT is presented in Figure 4C (detailed data are provided in the Supporting Information). Overall, the SP series polymers displayed performances that were similar to, or in some cases, superior to P‐1. Specific performance was compared by evaluating the sensor efficacy at a 60 s time point. Polymer P‐1 exhibited 34% and 61% quenching abilities at 30 and 60 s, respectively. Results for SP‐1 to SP‐4 were relatively lower at the 60 s mark, ranging from 23% to 42%. However, SP‐5 to SP‐7 demonstrated markedly superior quenching abilities, with SP‐5 at 74%, SP‐6 at 75%, and notably, SP‐7 at 88% and 94% at 30 and 60 s, respectively, confirming its rapid capability to detect nitroaromatic explosives (Figure 4D). Examination of results up to 300 s post‐exposure indicated that SP‐7 achieved quenching saturation within a short period. Comparing fluorescent quenching across different wavelengths highlighted the critical factor of film thickness, with notably better results obtained from thinner spin coatings (0.25 mg/1 mL, 120 s, >99%, Figure 4E).

The developed sensor also demonstrated excellent detection capabilities for TNT, a commonly used explosive material. SP‐7 showed 95% quenching ability within 180 s and achieved >99% quenching at 240 s. These findings, either matching or surpassing those of P‐1, indicate the potential suitability of SP‐7 for deployment in practical field sensors (Figure 4F). Pentiptycene‐derived explosive polymers are generally known to theoretically interact more favorably with electron‐deficient nitroaromatic explosives when the aromatic moieties of polymer possess electron‐donating substituents. However, in practice, the fluorescence quenching ability of polymer‐coated films can vary significantly due to a wide range of factors, including the thickness of the polymer coating, electrostatic interactions between the polymer and nitroaromatic compounds, interactions between polymer chains, and interactions between the polymer's side chains and nitroaromatic compounds. For example, as demonstrated by the Mishra group's study on the P‐2 polymer, the quenching efficiency of P‐2 polymer was observed to be higher than that of a polymer where the pyrene moiety is substituted with two electron‐donating *tert*‐butyl groups. Notably, SP‐5, which has a very similar structure to SP‐7 but differs only in the length of the alkyl chain, shows a quenching rate of 74% for 2,4‐DNT in 1 minute, which is significantly different from the quenching efficiency of SP‐7 (94%).

### 2.3 Photoluminescence Measurement & Mechanism Study

**Figure 5:**
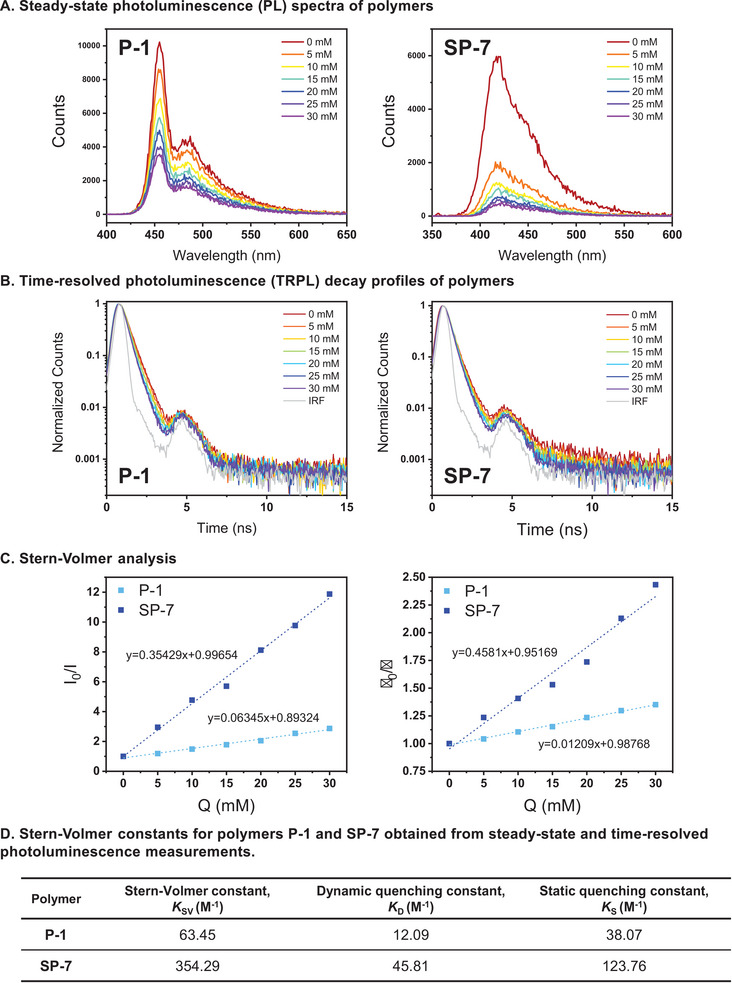
Photoluminescence measurement results of P‐1 and SP‐7 polymer solution using 2,4‐DNT.

Building on the gas‐phase quenching experiments, we further investigated the emission properties of the polymers P‐1 and SP‐7 in solution by exposing their chloroform solutions to DNT and performing both steady‐state and time‐resolved photoluminescence (PL) measurements. The target systems were prepared by varying the concentration of the quencher, 2,4‐DNT, from 0 to 30 mm in increments of 5 mm, while maintaining the polymer concentration (10 µg mL^−1^) where the steady‐state and time‐resolved PL spectra of the polymers are represented (**Figure** **5**A,B). As shown in the steady‐state PL spectra, the emission peaks were identified at ≈455 and 420 nm for P‐1 and SP‐7, respectively. The fluorescence spectra of SP‐7 have less defined vibronic features compared to that of P‐1. Both polymers exhibit a decrease in PL intensity with increasing DNT concentration. In the samples with the highest DNT concentration (30 mm), the intensity dropped by ca. 65% and 92% for P‐1 and SP‐7, respectively, compared to the samples without the quencher, indicating a higher quenching efficiency in SP‐7 (Table S1, Supporting Information). The changes in PL intensity with respect to the quencher concentration [Q] were analyzed using the Stern–Volmer (SV) relationship (Equation 1).^[^ ^39^ ^]^

(1) $$I_{0}/I=1+K_{SV}\left[Q\right]$$

where *I*_0_ and *I* are the fluorescence intensities in the absence and presence of the quencher, respectively, and *K_SV_* is the SV constant, indicating the extent of quenching. The SV plots of both polymers are shown where the plots of *I*_0_/*I* as a function of [Q] have linear relationships (Figure 5C). The *K_SV_* values were determined from the slopes, resulting in ca. 63 and 354 M^−1^ for P‐1 and SP‐7, respectively, as summarized (Figure 5D).

Next, we carried out time‐resolved PL measurements on the target systems using time‐correlated single photon counting (TCSPC) technique.^[^ ^40^ ^]^ Analyzing the decay profiles under the same conditions as in the steady‐state measurements, we obtained fluorescence lifetimes of the polymers (Table S1, Supporting Information). The lifetimes of the polymers without quencher were 450 and 209 ps for P‐1 and SP‐7, respectively. As the DNT concentration in the samples increased, both polymers showed a decrease in their fluorescence lifetimes. Compared to the samples without DNT, the lifetimes in the 30 mM DNT samples were shortened by 26.0% for P‐1 and 58.9% for SP‐7. The decrease in fluorescence lifetimes with increasing quencher concentration indicates that the polymers are under the influence of dynamic quenching process upon photoexcitation.^[^ ^41^ ^]^ The dynamic quenching refers to the process where quenching occurs as excited molecules interact with the quencher and lose energy, leading to emission quenching. In this process, the energy is dissipated via a nonradiative pathway by the quencher, resulting in a reduction of the excited state's lifetime.^[^ ^42^ ^]^ To analyze the dynamic quenching process quantitatively, we plotted the lifetime ratios with respect to quencher concentration [Q] to the SV relationship (Equation 2).^[^ ^43^ ^]^

(2) $$\tau_{0}/\tau =1+K_{D}\left[Q\right]$$

where τ_0_ and τ represent the lifetimes in the absence and presence of the quencher, respectively. *K_D_* is the dynamic quenching constant in the Stern–Volmer equation. τ_0_/τ increases linearly with increasing [Q], and the SP‐7 exhibits a steeper slope than P‐1 (Figure 5C). The *K_D_* values obtained from the slopes of the plots are listed, and the larger *K_D_* in SP‐7 reflects that dynamic quenching by DNT occurs more efficiently in SP‐7 than in P‐1.

Using the *K_SV_* and *K_D_* obtained from the steady‐state and time‐resolved PL measurements, we examined the extent to which the static quenching mechanism influences the two polymers.^[^ ^44^ ^]^ By applying the data to the modified Stern–Volmer equation (Equation 3),^[^ ^43^ ^]^ we calculated the static quenching constant, *K_S_*, to quantitatively determine the quenching mechanisms of the two polymers.

(3) $$\frac{I_{0}}{I}=\left(1+K_{D}\left[Q\right]\right)\left(1+K_{S}\left[Q\right]\right)$$

Using this equation, *K_S_*, the static quenching constant is obtained as summarized along with the SV constants (Figure 5D). By comparing the *K_S_* values, we confirmed that static quenching is more efficient in SP‐7 than in P‐1. Thus, both quenching mechanisms—dynamic and static—occur more efficiently in SP‐7 than in P‐1.

**Figure 6:**
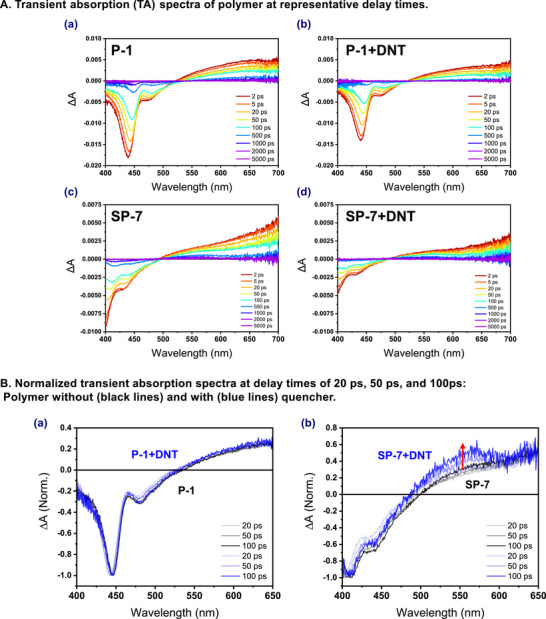
Transient absorption study of P‐1 and SP‐7 polymer solution using 2,4‐DNT.

To investigate the correct quenching mechanism, we conducted transient absorption (TA) measurements on the polymers where TA spectroscopy captures electronic transitions as a function of time upon photoexcitation. The TA measurements were performed in solutions of the two polymers in chloroform with and without the quencher (2,4‐DNT). The pump wavelength of 380 nm and the probe (white‐light continuum) spanning from 400 to 700 nm were used. TA spectra of polymers P‐1 and SP‐7 are exhibited at representative delay times after excitation (**Figure** **6**A).

First, we examined the TA spectra of the polymer systems without the quencher and observed that P‐1 exhibits a negative ΔA signal ≈440 nm, and SP‐7 ≈410 nm. These positions are close to the wavelength ranges where each polymer shows strong fluorescence, as indicated in the PL spectra (Figure 5A), and hence these negative signals are attributed to stimulated emission (SE) signals.^[^ ^45^ ^]^ In addition, the TA spectra exhibit ground‐state bleach (GSB) signals which typically appear as negative ground‐state absorption spectra. From the UV–Vis absorption spectra presented (Figure S1, Supporting Information), we confirm that ground‐state absorption occurs ≈430 and 350 nm for P‐1 and SP‐7, respectively. In the case of P‐1, the ground‐state absorption spectra appear at 430 nm, overlapping with the SE signals. For SP‐7, the ground‐state absorption occurs below 400 nm, combining with the SE signal to produce the strongest signal ≈400 nm. In both polymers, the SE signals decrease, and exhibit spectral redshifts. These redshifts are due to the spectral shifts from the SE signals and originate from the structural relaxation of the emitting species. Broad excited‐state absorption (ESA) signals were observed above 500 nm, which gradually decreases as a function of time.

When the quencher is present (with the same polymer concentration), the overall TA spectra feature similar spectral shape to those without the quencher. However, the SE signals seem to be reduced in the presence of the quencher.^[^ ^46^ ^]^ When the TA spectra at each delay time are normalized distinctions between the two polymers become apparent. The normalized TA spectra, based on the GSB and SE signals at ≈440 and 400 nm for P‐1 and SP‐7, respectively, are shown in the absence (black) and presence (blue) of the quencher (Figure 6B). In P‐1 with the DNT, a slight increase of ESA signals can be seen due to the known quenching dynamics through electron transfer from the polymer to the DNT observed while in the case of SP‐7 with the DNT, the stronger enhancement of ESA signals was detected in the similar region due to the more efficient quenching dynamics through photoinduced electron transfer (PET).^[^ ^8^ ^]^ Additionally, global analysis of the TA spectra using Glotaran software revealed a rise in the ESA signal within the evolution‐associated spectra (EAS) at the time constants corresponding to the lifetimes (Figure S2, Supporting Information).^[^ ^47^ ^]^ These results are in line with the fluorescence experimental results based on the Stern–Volmer constants where SP‐7 has the higher static and dynamic quenching constants than P‐1.

### 2.4 DFT Calculations

**Figure 7:**
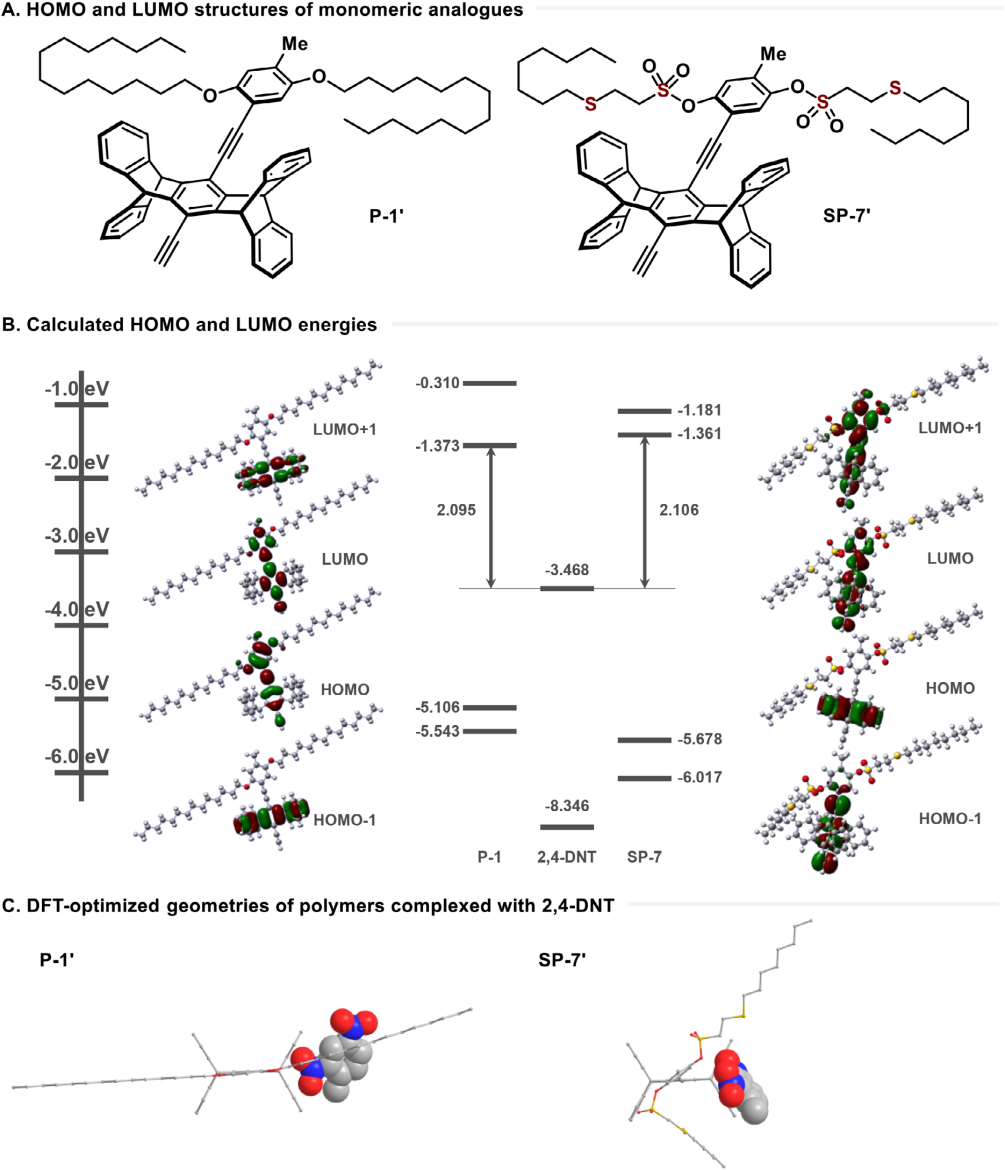
Optimized structures showing the binding sites of 2,4‐DNT to P‐1 and SP‐7.

To analyze why quenching occurs more efficiently in SP‐7 compared to P‐1, we conducted density functional theory (DFT)‐based computational studies to understand the electronic structures of the monomers and interpret the properties of the polymers. For the monomer calculations, we simplified the structures by capping both ends of the polymers with methyl and hydrogen groups, forming monomer units (**Figure** **7**A). Calculations were performed using Gaussian software, employing the B3LYP/6‐31G(d) functional and basis set to optimize the monomer structures.^[^ ^48^ ^]^ From the DFT calculations, we analyzed the molecular orbitals of the optimized monomers, determining the energies of the highest occupied molecular orbital (HOMO) and the lowest unoccupied molecular orbital (LUMO) along with their surrounding energy levels. We illustrated the electron distributions of each orbital (Figure 7B). The LUMO energies of the polymers were calculated to be −1.373 and −1.361 eV for P‐1 and SP‐7. Compared to the optimized LUMO energy level of 2,4‐DNT at −3.468 eV, it is predicted that the greater driving force in SP‐7, which has a higher LUMO energy level, contributes to a more efficient PET mechanism.^[^ ^8^ ^]^ In practice, the SP‐7 polymer exhibits superior fluorescence quenching efficiency compared to the P‐1 polymer. This polymer structure, incorporating organosulfur side chains, represents an optimal design for real‐world explosive fluorescence detection sensors. Thus, the DFT calculations demonstrated that the unit structure of the SP‐7 polymer, which includes sulfide and sulfonate ester substituents, is more favorable for fluorescence quenching via the PET mechanism compared to the unit structure of the P‐1 polymer, which features a long alkyl ether structure. Subsequently, a positive charge (+1) was assigned to the donor, and ground‐state geometry optimizations were performed, followed by time‐dependent density functional theory (TD‐DFT) calculations on each charged species. Both the optimizations and TD‐DFT calculations were carried out using the uB3LYP/6‐31G(d) basis set (Tables S2 and S3, Supporting Information). For the P‐1 radical cation, a significant transition was observed at 663.7 nm with an oscillator strength of 0.0803, whereas for SP‐7, the corresponding transition at 656.5 nm exhibited a much lower oscillator strength of 0.0029. These findings are consistent with the peaks in the transient absorption (TA) spectra, where both P‐1 and SP‐7 show weak transient signals in the 650–700 nm region.

To identify where 2,4‐DNT preferentially interacts with each polymer unit, we carried out DFT optimizations of neutral complexes pairing one 2,4‐DNT molecule with each monomer model (Figure 7C). In the P‐1 complex, DNT is located adjacent to the pentiptycene core, whereas the long alkoxy side chains are oriented away from the analyte. In the SP‐7 complex, one nitro group of DNT is directed toward the sulfide/sulfonate ester side chain, positioning the explosive closer to the donor backbone than in P‐1. This geometry supports the higher fluorescence‐quenching efficiency experimentally observed for SP‐7.

### 2.5 TLC‐Spotting Sensor: Rapid Detection of the Explosive

**Figure 8:**
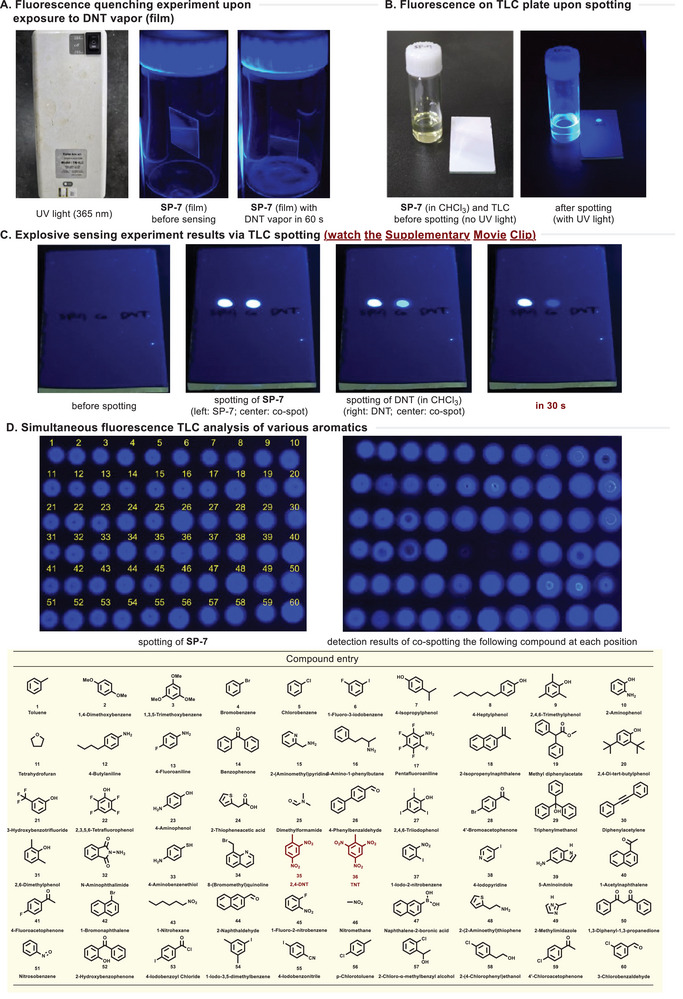
Simple operation of TLC‐spotting detection of explosive molecule.

We aimed to develop a more straightforward and practical detection method, like a TLC‐spotting sensor, that can be efficiently utilized in actual field conditions (**Figure** **8**). As a first step, we tested whether the developed glass‐coated SP‐7 film could function as a fluorescent sensor. A commonly used laboratory UV lamp (365 nm) was employed as the excitation source. A glass chamber containing a solid DNT reagent was equipped to generate vapor, and the resulting quenching effect was visually examined. However, no significant difference was observed with the naked eye (Figure 8A). Interestingly, an apparent fluorescence change was detected when using thin‐layer chromatography (TLC, Merck, Kieselgel 60 F254 0.25 mm), one of the most fundamental and widely accessible analytical tools in chemical researches. Due to the strong emission of SP‐7, the analyte could be directly visualized by simple TLC spotting (Figure 8B).

To further evaluate the sensing capability, co‐spotting of SP‐7 and DNT dissolved in CHCl_3_ (1.0 mg mL^−1^) was performed. Remarkably, an intense fluorescence quenching was observed within very short time (<30 s), clearly indicating the presence of DNT explosives. This remarkable change is captured in the Supporting Information Movie Clip (Figure 8C: watch the Supporting Information Movie Clip). Furthermore, to assess the chemical selectivity of TLC‐spotting sensors for explosive molecules, various aromatic analogue compounds were tested.

Additionally, to assess the chemical selectivity of the TLC‐spotting sensor for explosive molecules, various aromatic analogue compounds were tested. Within just a few seconds, all entries quickly reached a stabilized signal. Notably, out of the sixty tested compounds (entries 1–60), only DNT (entry 35) and TNT (entry 36) displayed significant quenching responses. The mono‐nitro‐substituted aromatic compound (entry 37) exhibited a very weak quenching response, while nitroalkanes (entries 43 and 46) displayed no response. Additionally, other functional groups, including alkanes, alkenes, alkynes, alcohols, amines, anilines, phenols, halides, ketones, aldehydes, *O, S, N*‐heteroaromatics, boronic acids, and perfluoro compounds, were all inactive. These results demonstrate that the TLC‐spotting sensor serves as a highly rapid, straightforward, and cost‐effective platform for explosive detection, even in real‐field conditions where advanced analytical instruments are not readily available (Figure 8D).

## 3 Conclusion

In summary, we have developed the SP series polymers, pentiptycene‐incorporated conjugated polymers with electron‐rich structures, designed as fluorescence quenching chemical sensors for nitroaromatic explosives. The synthesis of the SP polymers harnessed novel SuFEx click chemistry, allowing chemoselective and reliable scale‐up production of materials containing diverse alkyl structures, including both sulfide and sulfonate ester linkages. These structures exhibited significant water‐tolerance and bench stability during synthesis, advantageous characteristics when fabricated into actual sensors. Fluorescence quenching experiments with DNT and TNT revealed that SP‐7 exhibits comparable, and in the case of DNT, superior sensing performance to previously developed polymer sensors. The excellent solubility of SP‐7 enabled straightforward film fabrication via spin coating, where thinner films demonstrated enhanced sensing capabilities. Time‐resolved spectroscopic measurements further confirmed the superior sensing properties of SP‐7 in both static and dynamic quenching processes compared to P‐1. Steady‐state and time‐resolved photoluminescence studies revealed higher Stern–Volmer constants for SP‐7, highlighting its efficient quenching processes. Additionally, transient absorption spectroscopy identified stronger ESA signals in SP‐7, which indicates the PET mechanism drives the quenching processes. Complementary DFT calculations provided insights into the electronic structure of SP‐7, attributing its high sensing efficiency to favorable energy levels and orbital spatial distributions. Our results shed light on the intricate quenching dynamics and electron transfer mechanisms in polymer‐DNT systems, underscoring the potential of SP‐7 as an effective and versatile sensor material. Finally, we developed a rapid and highly selective explosive detection of the TLC‐spotting method using simple UV irradiation without requiring specialized analytical instruments. We believe this technique is expected to be highly useful for real‐field applications.

## Conflict of Interest

The authors declare no conflict of interest.

## Supporting information



Supporting Information

Supplemental Movie 1

## Data Availability

The data that support the findings of this study are available in the supplementary material of this article.
