# Supplementary material for: SuFEx‐Enabled Catalytic Synthesis of Fluorescent Organosulfur Polymers for the Rapid Detection of Explosives
Source: Adv Sci (Weinh). 2025 Jul 11;12(38):e06616. doi: 10.1002/advs.202506616 (PMC12520564; doi:10.1002/advs.202506616)
Supplement: Supplementary file 1 — Supporting Information [file ADVS-12-e06616-s001.pdf]

ADVANCED SCIENCE
Open Access

Supporting Information

for *Adv. Sci.*, DOI 10.1002/advs.202506616

# SuFEx-Enabled Catalytic Synthesis of Fluorescent Organosulfur Polymers for the Rapid Detection of Explosives

*Sun Bu Lee, Jaeyoung Heo, Seunghyun Noh, Tae Eun An, Gyeongsoo Kim, Gang Min Lee, Junggong Kim, Keunyoung Kim, Jongman Lee, Taeyeon Kim*, Changsik Song* and Han Yong Bae**

*Supplementary Information*

# SuFEx-Enabled Catalytic Synthesis of Fluorescent Organosulfur Polymers for the Rapid Detection of Explosives

*Sun Bu Lee,[a†] Jaeyoung Heo,[a†] Seunghyun Noh,[a†] Tae Eun An,[a] Gyeongsoo Kim,[a] Gang Min Lee,[a,b] Junggong Kim,[c] Keunyoung Kim,[c] Jongman Lee,[c] Taeyeon Kim,*[a] Changsik Song,*[a] Han Yong Bae*[a]*

[a]Department of Chemistry, Sungkyunkwan University, Suwon, 16419, Republic of Korea

[b]Present address: RIST, Gwangyang, Jeollanam-do, 57801, Republic of Korea.

[c]PNL Global Co., Ltd., B1, 10 Sapyeong-daero 2-gil, Seocho-gu, Seoul 06652, Republic of Korea

*Corresponding author. Email: hybae@skku.edu (HYB); songcs@skku.edu (CS); taeyeon@skku.edu (TK)

[†]These authors contributed equally.

## Table of contents


1. General information ..................... S3

2. General procedure for the thia-Michael addition ..................... S5

3. General procedure for the SuFEx conjugation ..................... S6

4. Sonogashira polymerization for the SP polymers ..................... S7

5. Characterization data for products ..................... S8

6. Analytical data of polymers ..................... S17

7. Photoluminescence measurement ..................... S19

8. DFT calculations ..................... S22

9. NMR spectra ..................... S25

10 HR-MS spectra ..................... S43

11. Reference ..................... S50

## 1. General information

■ **Materials and instruments:** Unless stated otherwise, Chemicals were purchased from commercial suppliers (e.g., Aldrich, Alfa Aesar, and TCI). HPLC solvents, anhydrous solvents, NMR solvents, and additional organic solvents were purchased from commercial suppliers (e.g., Aldrich, Alfa Aesar, Merck, and Wako) and used without distillation or purification. Chemicals were purchased from commercial vendors (e.g., Aldrich, Alfa Aesar, TCI) and used as received unless otherwise stated. Starting materials for polymerization such as pentiptycene acetylene and 2,5-diiodobenzene-1,4-diol were synthesized by following known reference.[1,2] P-1 polymer and monomer for P-1 polymer were synthesized with reference to previous literature.[3-5] HPLC grade solvents, anhydrous solvents, NMR solvents, and additional organic solvents were purchased from commercial vendors (e.g., Aldrich, Alfa Aesar, CIL Inc., Merck, Wako) and used without further distillation or purification. Column chromatographic purification processes were performed by using silica gel (Merck, 60 Å, 230–400 mesh, 0.040–0.063 mm). Mass data acquired on a Supercritical Fluid Chromatography combined with Xevo G2-XS QTOF Mass Spectrometer (Waters, Milford, MA, USA) at the Chiral Material Core Facility Center of Sungkyunkwan University. Melting points were measured by using Büchi® B–540 at the Chiral Material Core Facility Center for Sungkyunkwan University. $^1$H (500 MHz), $^{13}$C (125 MHz) spectra were recorded using Bruker Ascend 500 spectrometer at the Chiral Material Core Facility Center of Sungkyunkwan University. Chemical shifts (δ) were reported by using tetramethylsilane as an internal standard in $CDCl_3$. UV–visible (UV–vis) absorption measurements were carried out using a UV-1800 (Shimadzu, Kyoto, Japan) spectrophotometer under ambient conditions (i.e., at room temperature and in the air) using a 1.0 cm quartz cell. The average molecular weight and polydispersity index (PDI) of the polymers were determined utilizing Agilent Technology 1260 Infinity equipment (Agilent, CA, USA) via gel permeation chromatography (GPC) with tetrahydrofuran (THF) as the eluent and polystyrene as the standard. The polymer sample was dissolved in 3 mg/mL in THF, filtered using a 0.2 um PVDF filter, and measured by GPC under the condition of flow rate = 1 mL/min. PL spectra were recorded on a FluoroMate FS-2 fluorescence spectrophotometer (SCINCO, Seoul, Korea). Quantum yield was also measured using a device that measures PL. The

polymer film was coated on a glass plate under the condition of 0.5 mg/ml chloroform and used in a fluorescence quenching experiment. UV and PL data were measured by dissolving the polymer in dichloromethane and filtering it with 0.2 um PVDF. PL and time-correlated single-photon counting (TCSPC) measurements were performed using an FS5 spectrofluorometer (Edinburgh Instruments, Livingston, UK). For the steady-state PL measurements, a continuous-wave xenon lamp served as the excitation source, with an excitation wavelength of 430 nm for sample P-1 and 355 nm for sample SP-7. The time-resolved measurements utilized a picosecond pulsed diode laser at 401.7 nm in a TCSPC configuration. All samples for PL and TCSPC were prepared at a concentration of 10 µg/mL in a standard 1 cm path length, 4-sided quartz cuvette under ambient conditions. Emission signals were collected by the system's built-in photomultiplier tube, and lifetime fitting was conducted using the proprietary Fluoracle software. Transient absorption (TA) measurements were carried out using the fundamental pulsed laser from a 1 kHz Ti:Sapphire amplifier (Legend Elite, Coherent, USA), which provided sub-25 fs pulses centered at 790 nm. The output was split into two beams: one beam was directed toward an optical parametric amplifier (TOPAS Prime, Light Conversion, Lithuania) and tuned to 380 nm as a pump pulse (with a pulse energy of ~1 nJ per pulse), while the other beam was focused onto a nonlinear crystal to generate a broadband white-light continuum probe. For TA measurements, the sample solutions were prepared at 16 µg/mL for P-1 and 24 µg/mL for SP-7. The probe beam tracked the pump-induced absorption changes over time by varying the delay between the pump and probe pulses. A TA spectrometer (HELIOS, Ultrafast System, USA) equipped with a 1024-pixel CMOS and 256-pixel InGaAs sensor was used to record the TA spectra. Kinetic time constants and spectra were extracted through global fitting and singular value decomposition using Surface Xplorer software and Glotaran software. Density functional theory (DFT)-based computational studies were conducted on simplified monomer models. Each polymer was truncated into a monomer unit by capping both ends with methyl and hydrogen groups. Geometry optimizations were carried out in Gaussian (Gaussian, Inc., USA) using the B3LYP functional with the 6-31G(d) basis set.

## 2. General procedure for the thia-Michael addition

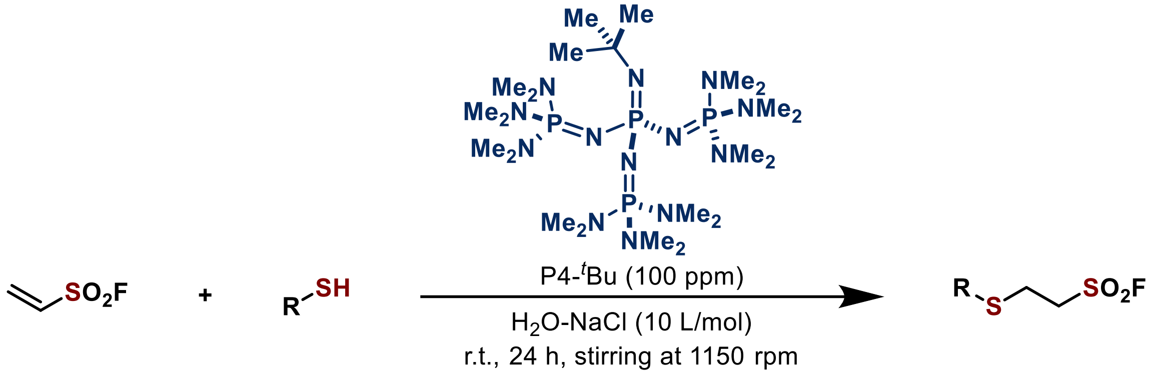


**2 equiv. (12 mmol)** **1 equiv. (6 mmol)** **1** to **7**

To a 250 mL round bottom flask charged with a magnetic bar, $H_2O$-NaCl (sat.) 60 mL was added. Then, thiol (6 mmol, 1 equiv.), ethenesulfonyl fluoride (12 mmol, 2 equiv.), and **P4-*t*Bu** (100 ppm = 0.01 mol%) were added and stirred at room temperature for 24 hours. The reaction mixture was extracted using ethyl acetate and the organic layer was filtered using $Na_2SO_4$. Filtered organic layer was evaporated under reduced pressure until ESF is completely blown away to afford desired product **1** to **7** without other purification.

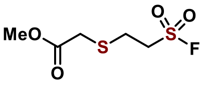


**1**
66% yield
**0.86 g**

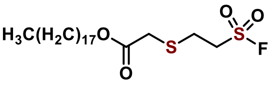


**2**
99% yield
**2.70 g**

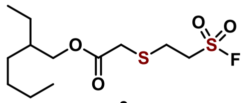

**3**
99% yield
**2.00 g**

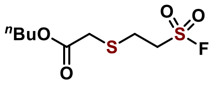


**4**
99% yield
**1.54 g**

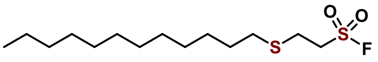

**5**[a]
94% yield
**1.77 g**

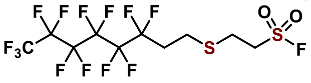


**6**
91% yield
**2.68 g**

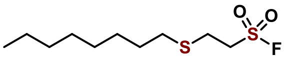

**7**
93% yield
**1.43 g**

[a] 1000 ppm of P4-*t*Bu was used.

## 3. General procedure for the SuFEx conjugation

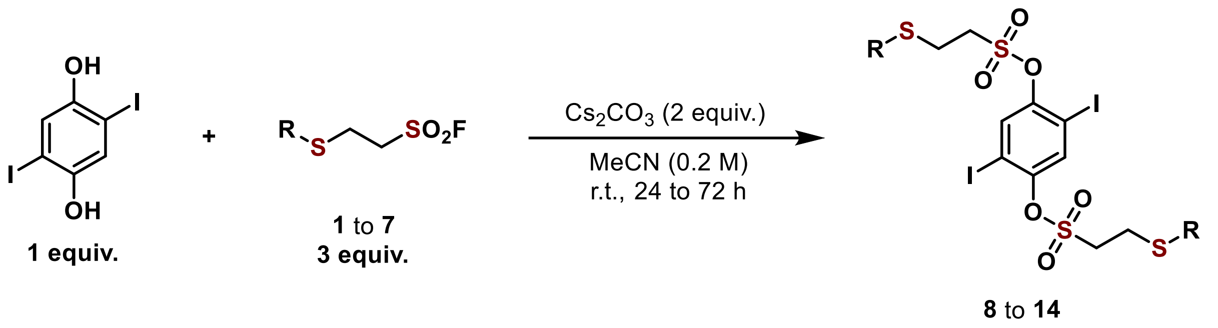


To a flame dried 5 mL vial charged with magnetic bar, thia-Michael adduct **1** to **7** (3 equiv.), 2,5-diiodobenzene-1,4-diol (0.5 mmol, 1 equiv.) and cesium carbonate (2 equiv.) were added under argon atmosphere. Subsequently anhydrous acetonitrile (0.2 M) was added to vial using syringe. The reaction mixture was stirred at room temperature for 24-72 hours. After the reaction time, crude mixture was concentrated in vacuo, and the residue was purified by column chromatography on silica gel to afford desired **8** to **14**.

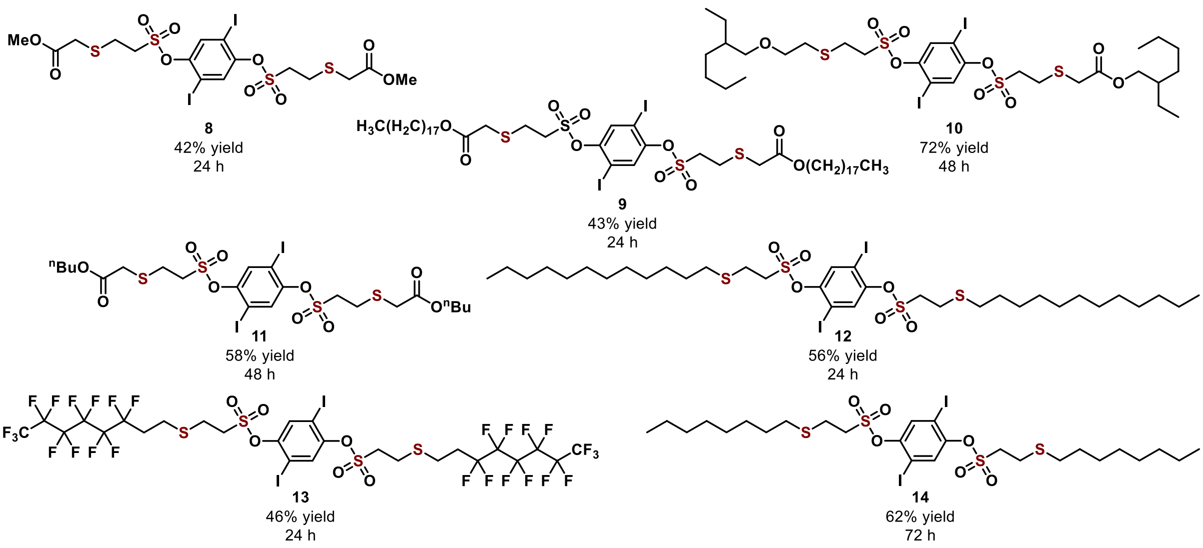

## 4. Sonogashira polymerization for the SP polymers

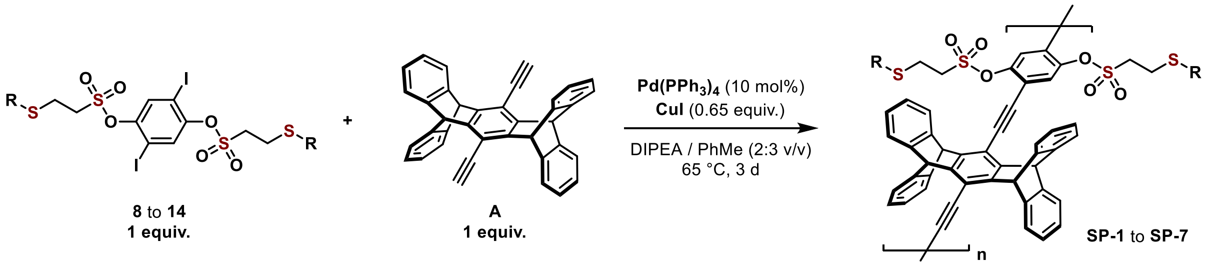


To a flame dried 20 mL vial, **Pd(PPh₃)₄** (10 mol%), **CuI** (0.65 equiv.), pentiptycene acetylene (0.2 mmol, 1 equiv.) and SuFEx product **8** to **14** (0.2 mmol, 1 equiv.) were added under argon atmosphere, followed by adding *N,N*-Diisopropylethylamine/toluene (2:3 v/v, 6 mL). Then reaction mixture was stirred at 65 °C for 3 days. Crude mixture was extracted three times with water and chloroform. The combined organic layers was washed with $NH_4Cl$ saturated water and dried with $Na_2SO_4$. After evaporation, the residue was reprecipitated in chloroform/methanol. The resulting polymer was filtered and dried in vacuo to afford desired polymers **SP-1** to **SP-7**.

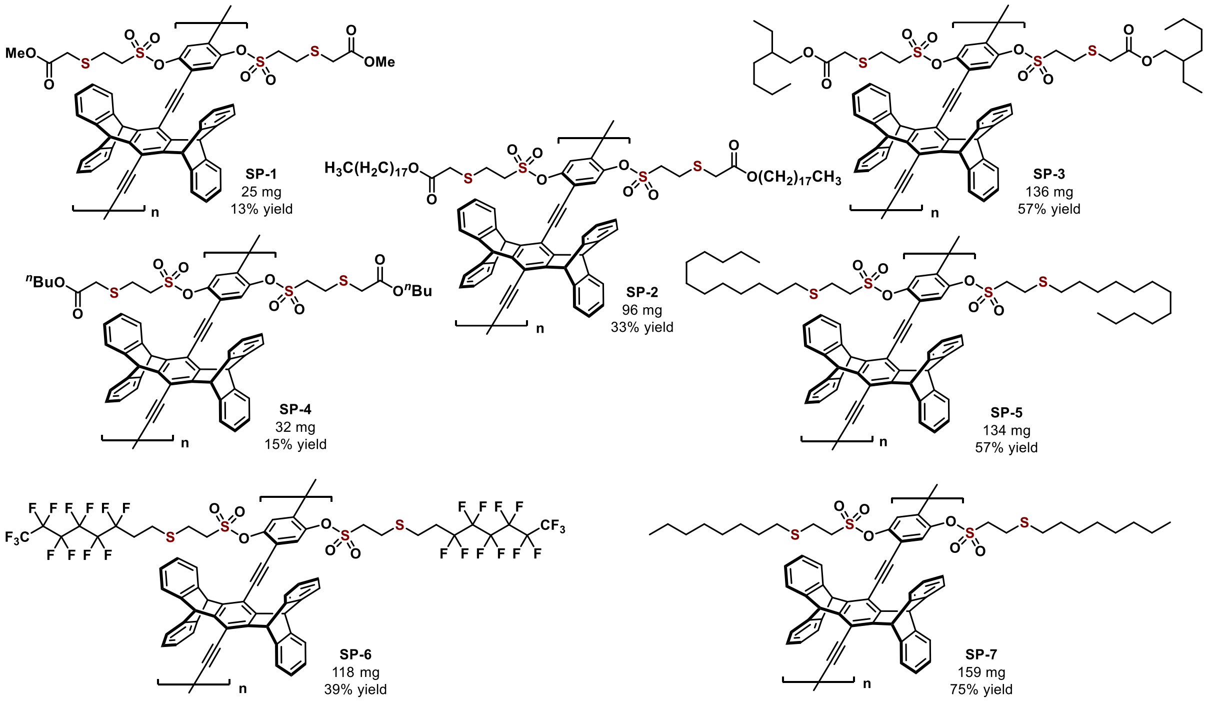

## 5. Characterization data for products

**Methyl 2-((2-(fluorosulfonyl)ethyl)thio)acetate (1)**

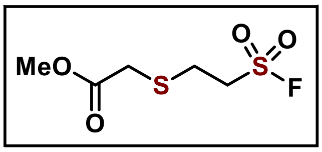


The compound was prepared by following general procedure for the thia-Michael addition to afford **1** as a yellow liquid (0.86 g, 66% yield); $R_f$ = 0.27 (20% ethyl acetate in hexane); $^1$H NMR (500MHz, $CDCl_3$) δ 3.74 (s, 3H), 3.73 – 3.70 (m, 2H), 3.29 (s, 2H), 3.13 – 3.10 (m, 2H); $^{13}$C NMR (125 MHz, $CDCl_3$) δ 170.4, 52.8, 50.8 (d, *J* (C-$SO_2$F) = 15.6 Hz), 33.7, 25.9.

**2-((3-(Heptadecyloxy)-2-oxopropyl)thio)ethane-1-sulfonyl fluoride (2)**

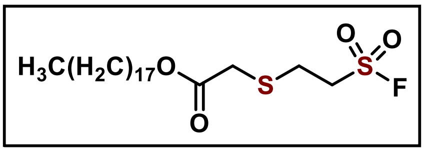


The compound was prepared by following general procedure for the thia-Michael addition to afford **2** as a white solid (2.7 g, 99% yield); $R_f$ = 0.42 (20% ethyl acetate in hexane); $^1$H NMR (500MHz, $CDCl_3$) δ 4.14 (t, *J* = 6.8 Hz, 2H), 3.75 – 3.71 (m, 2H), 3.29 (s, 2H), 3.16 – 3.13 (m, 2H), 1.68 – 1.62 (m, 2H), 1.34 – 1.25 (m, 30H), 0.87 (t, *J* = 6.9 Hz, 3H); $^{13}$C NMR (125 MHz, $CDCl_3$) δ 170.0, 66.2, 50.9 (d, *J* (C-$SO_2$F) = 15.8 Hz), 34.1, 32.1, 29.82, 29.79, 29.76, 29.7, 29.6, 29.5, 29.3, 28.6, 26.0, 25.9, 22.8, 14.2.

**2-Ethylhexyl 2-((2-(fluorosulfonyl)ethyl)thio)acetate (3)**

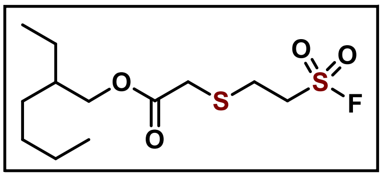


The compound was prepared by following general procedure for the thia-Michael addition to afford **3** as a yellow liquid (2.0 g, 99% yield); $R_f$ = 0.40 (20% ethyl acetate in hexane); $^1$H NMR (500MHz, $CDCl_3$) δ 4.09 – 4.02 (m, 2H), 3.74 – 3.70 (m, 2H), 3.28 (s, 2H), 3.13 (dd, *J* = 8.2, 7.7 Hz, 2H), 1.59 (dt, *J* = 11.9, 5.8 Hz, 1H), 1.38 – 1.32 (m, 2H), 1.27 – 1.24 (m, 6H), 0.88 (t, *J* = 7.4 Hz, 6H); $^{13}$C NMR (125 MHz, $CDCl_3$) δ 170.1, 68.3, 50.8 (d, *J* (C-$SO_2$F) = 15.6 Hz), 38.8, 33.9, 30.3, 28.9, 25.9, 23.7, 23.0, 14.0, 10.9.

**Butyl 2-((2-(fluorosulfonyl)ethyl)thio)acetate (4)**

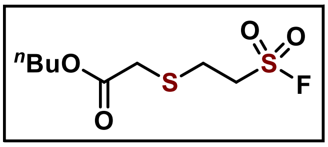


The compound was prepared by following general procedure for the thia-Michael addition to afford **4** as a yellow liquid (1.54 g, 99% yield); $R_f$ = 0.35 (20% ethyl acetate in hexane); $^1$H NMR (500MHz, $CDCl_3$) δ 4.15 (t, *J* = 6.7 Hz, 2H), 3.75 – 3.70 (m, 2H), 3.28 (s, 2H), 3.15 – 3.12 (m, 2H), 1.66 – 1.61 (m, 2H), 1.42 – 1.35 (m, 2H), 0.93 (t, *J* = 7.4 Hz, 3H); $^{13}$C NMR (125 MHz, $CDCl_3$) δ 170.0, 65.7, 50.7 (d, *J* (C-$SO_2$F) = 15.4 Hz), 33.8, 30.5, 25.8, 19.0, 13.6.

**2-(Dodecylthio)ethane-1-sulfonyl fluoride (5)**

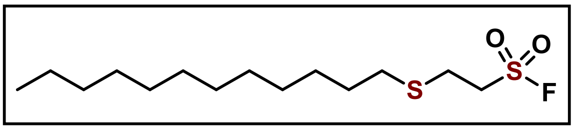


The compound was prepared by following general procedure for the thia-Michael addition to afford **5** as a yellow liquid (1.77 g, 94% yield); $R_f$ = 0.75 (20% ethyl acetate in hexane); $^1$H NMR (500MHz, $CDCl_3$) δ 3.62 – 3.58 (m, 2H), 3.01 – 2.97 (m, 2H), 2.59 – 2.56 (m, 2H), 1.63 – 1.57 (m, 2H), 1.39 – 1.35 (m, 2H), 1.30 – 1.26 (m, 16H), 0.88 (t, *J* = 6.9 Hz, 3H); $^{13}$C NMR (125 MHz, $CDCl_3$) δ 51.3 (d, *J* (C-$SO_2$F) = 15.1 Hz), 32.5, 32.0, 29.76, 29.75, 29.7, 29.6, 29.5, 29.4, 29.3, 28.9, 25.0, 22.8, 14.2.

**2-((3,3,4,4,5,5,6,6,7,7,8,8,8-Tridecafluorooctyl)thio)ethane-1-sulfonyl fluoride (6)**

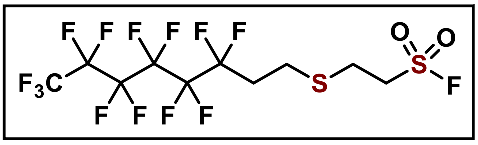


The compound was prepared by following general procedure for the thia-Michael addition to afford **6** as a yellow liquid (2.68 g, 91% yield); $R_f$ = 0.40 (20% ethyl acetate in hexane); $^1$H NMR (500MHz, $CDCl_3$) δ 3.65 – 3.61 (m, 2H), 3.08 – 3.05 (m, 2H), 2.84 (dd, *J* = 9.3, 6.9 Hz, 2H), 2.41 (ddd, *J* = 25.9, 17.6, 8.1 Hz, 2H); $^{13}$C NMR (125 MHz, $CDCl_3$) δ 119.5 (t, *J* = 31.8 Hz), 118.5 (t, *J* = 33.7 Hz), 117.5 (t, *J* = 31.9 Hz), 116.2 (t, *J* = 33.2 Hz), 115.5 (t, *J* = 31.7 Hz), 113.9 – 112.2 (m), 111.4 – 110.1 (m), 109.4 – 108.0 (m), 106.7 – 105.9 (m), 50.9 (d, *J* (C-$SO_2$F) = 16.1 Hz), 31.8 (t, *J* (C-$CF_2$) = 22.1 Hz), 25.4, 23.3.

**2-(Octylthio)ethane-1-sulfonyl fluoride (7)**

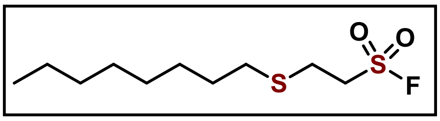


The compound was prepared by following general procedure for the thia-Michael addition to afford **7** as a yellow liquid (1.43 g, 93% yield); $R_f$ = 0.71 (20% ethyl acetate in hexane); $^1$H NMR (500MHz, $CDCl_3$) δ 3.62 – 3.56 (m, 2H), 3.00 – 2.97 (m, 2H), 2.58 – 2.55 (m, 2H), 1.62 – 1.56 (m, 2H), 1.39 – 1.35 (m, 2H), 1.31 – 1.27 (m, 8H), 0.88 (t, $J$ = 6.9 Hz, 3H); $^{13}$C NMR (125 MHz, $CDCl_3$) δ 51.3 (d, $J$ (C-$SO_2$F) = 15.1 Hz), 32.5, 31.9, 29.4, 29.3, 29.2, 28.8, 25.0, 22.7, 14.2.

**Dimethyl 2,2'-((((4-(hydrosulfonyloxy)-2,5-diiodophenoxy)sulfonyl)bis(ethane-2,1-diyl))bis(sulfanediyl))diacetate (8)**

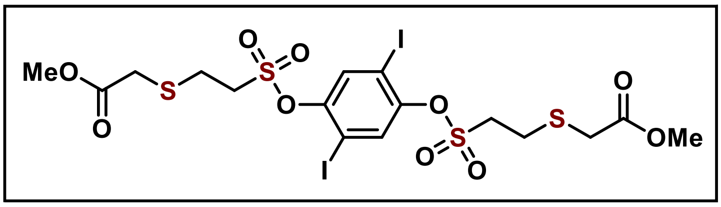


The compound was prepared by following general procedure for the SuFEx conjugation and purified by silica gel column chromatography (ethyl acetate/hexanes 1:49 to 1:19) afforded **8** as a pale yellow solid (0.16 g, 42% yield); $R_f$ = 0.15 (20% ethyl acetate in hexane); m.p. = 130–132 °C; $^1$H NMR (500MHz, $CDCl_3$) δ 7.86 (s, 2H), 3.78 – 3.75 (m, 10H), 3.33 (s, 4H), 3.29 – 3.26 (m, 4H); $^{13}$C NMR (125 MHz, $CDCl_3$) δ 170.4, 147.9, 133.1, 89.7, 52.9, 52.7, 33.9, 26.4; HRMS (ESI) m/z: $[M + Na]^+$ Calcd for $C_{16}H_{20}I_2O_{10}S_4Na$ 776.7926; Found 776.7922.

**Dioctadecyl 2,2'-((((4-(hydrosulfonyloxy)-2,5-diiodophenoxy)sulfonyl)bis(ethane-2,1-diyl))bis(sulfanediyl))diacetate (9)**

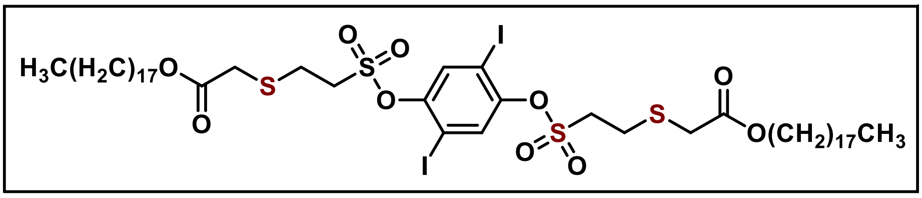


The compound was prepared by following general procedure for the SuFEx conjugation and purified by silica gel column chromatography (ethyl acetate/hexanes 1:49 to 1:19) afforded **9** as a yellow solid (0.13 g, 43% yield); $R_f$ = 0.43 (20% ethyl acetate in hexane); m.p. = 81–83 °C; $^1$H NMR (500MHz, $CDCl_3$) δ 7.86 (s, 2H), 4.15 (t, $J$ = 6.8 Hz, 4H), 3.78 – 3.75 (m, 4H), 3.31 (s, 4H), 3.30 – 3.26 (m,

4H), 1.68 – 1.63 (m, 4H), 1.36 – 1.25 (m, 60H), 0.88 (t, $J$ = 6.9 Hz, 6H); $^{13}$C NMR (125 MHz, $CDCl_3$) δ 170.1, 147.9, 133.1, 89.7, 66.2, 52.8, 34.2, 32.1, 29.85, 29.8, 29.73, 29.66, 29.5, 29.4, 28.7, 26.4, 26.0, 22.8, 14.3; HRMS (ESI) m/z: $[M + Na]^+$ Calcd for $C_{50}H_{88}I_2O_{10}S_4Na$ 1253.3247; Found 1253.3250.

**2-Ethylhexyl 2-((2-((4-(((2-((2-((2-ethylhexyl)oxy)ethyl)thio)ethyl)sulfonyl)oxy)-2,5-diiodophenoxy)sulfonyl)ethyl)thio)acetate (10)**

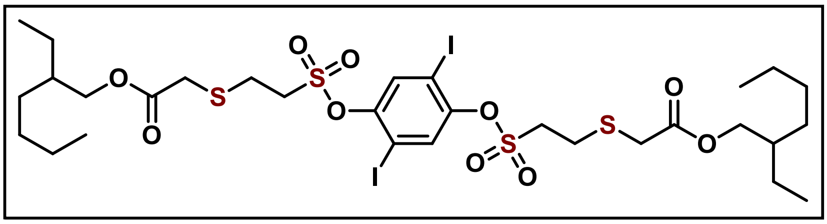

The compound was prepared by following general procedure for the SuFEx conjugation and purified by silica gel column chromatography (ethyl acetate/hexanes 1:49 to 1:19) afforded **10** as a white solid (0.34 g, 72% yield); $R_f$ = 0.30 (20% ethyl acetate in hexane); m.p. = 53–55 °C; $^1$H NMR (500MHz, $CDCl_3$) δ 7.85 (s, 2H), 4.11 – 4.04 (m, 4H), 3.78 – 3.75 (m, 4H), 3.32 (s, 4H), 3.29 – 3.26 (m, 4H), 1.60 (dt, $J$ = 12.2, 6.1 Hz, 2H), 1.40 – 1.33 (m, 4H), 1.33 – 1.24 (m, 12H), 0.91 – 0.88 (m, 12H); $^{13}$C NMR (125 MHz, $CDCl_3$) δ 170.2, 147.9, 133.1, 89.7, 68.4, 52.8, 38.8, 34.2, 30.4, 29.0, 26.4, 23.8, 23.1, 14.2, 11.1; HRMS (ESI) m/z: $[M + Na]^+$ Calcd for $C_{30}H_{48}I_2O_{10}S_4Na$ 973.0117; Found 973.0122.

**Dibutyl 2,2'-((((4-(hydrosulfonyloxy)-2,5-diiodophenoxy)sulfonyl)bis(ethane-2,1-diyl))bis(sulfanediyl))diacetate (11)**

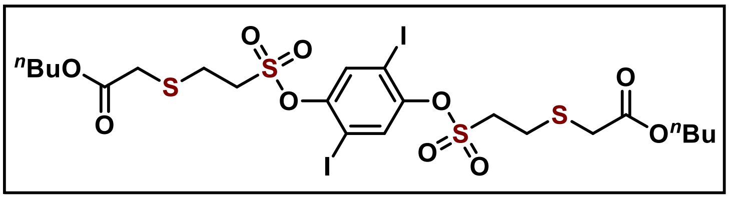

The compound was prepared by following general procedure for the SuFEx conjugation and purified by silica gel column chromatography (ethyl acetate/hexanes 1:49 to 1:19) afforded **11** as a white solid (0.25 g, 58% yield); $R_f$ = 0.18 (20% ethyl acetate in hexane); m.p. = 67–69 °C; $^1$H NMR (500MHz, $CDCl_3$) δ 7.85 (s, 2H), 4.16 (t, $J$ = 6.7 Hz, 4H), 3.78 – 3.75 (m, 4H), 3.31 (s, 4H), 3.31 – 3.26 (m, 4H), 1.67 – 1.61 (m, 4H), 1.43 – 1.35 (m, 4H), 0.93 (t, $J$ = 7.4 Hz, 6H); $^{13}$C NMR (125 MHz, $CDCl_3$) δ 170.0, 147.9, 133.1, 89.7, 65.9, 52.7, 34.2, 30.7, 26.4, 19.2, 13.8; HRMS (ESI) m/z: $[M + Na]^+$ Calcd for $C_{22}H_{32}I_2O_{10}S_4Na$ 860.8865;

Found 860.8859.

**2,5-Diiodo-1,4-phenylene bis(2-(dodecylthio)ethane-1-sulfonate) (12)**

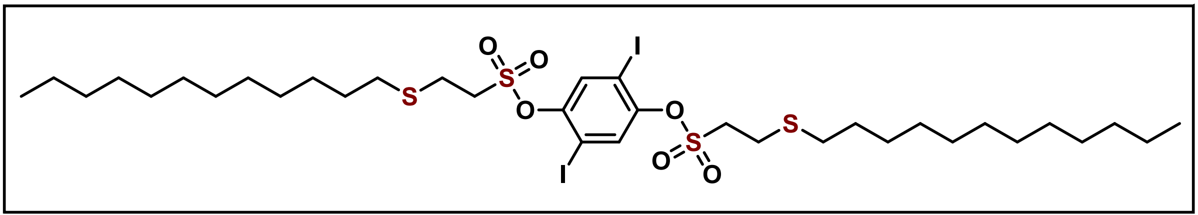

The compound was prepared by following general procedure for the SuFEx conjugation and purified by silica gel column chromatography (ethyl acetate/hexanes 1:49 to 1:19) afforded **12** as a white solid (0.26 g, 56% yield); $R_f$ = 0.71 (20% ethyl acetate in hexane); m.p. = 90–92 °C; $^1$H NMR (500MHz, $CDCl_3$) δ 7.85 (s, 2H), 3.68 – 3.65 (m, 4H), 3.14 – 3.11 (m, 4H), 2.60 (t, *J* = 7.4 Hz, 4H), 1.62 (dt, *J* = 15.0, 7.4 Hz, 4H), 1.40 – 1.36 (m, 4H), 1.31 – 1.26 (m, 32H), 0.88 (t, *J* = 6.9 Hz, 6H); $^{13}$C NMR (125 MHz, $CDCl_3$) δ 147.9, 133.1, 89.5, 53.2, 32.6, 32.1, 29.79, 29.78, 29.73, 29.66, 29.6, 29.5, 29.3, 28.9, 25.4, 22.8, 14.3; HRMS (ESI) m/z: $[M + H]^+$ Calcd for $C_{34}H_{61}I_2O_6S_4$ 947.1440; Found 947.1433.

**2,5-Diiodo-1,4-phenylene bis(2-((3,3,4,4,5,5,6,6,7,7,8,8,8-tridecafluorooctyl)thio)ethane-1-sulfonate) (13)**

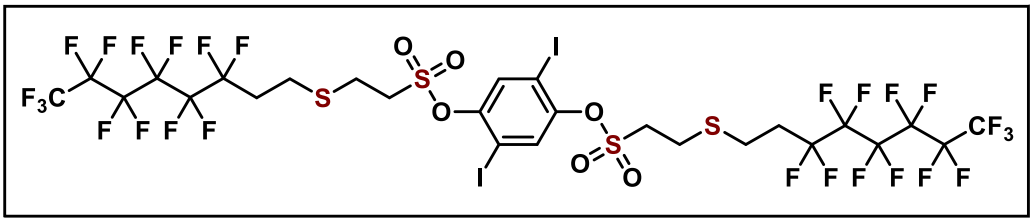

The compound was prepared by following general procedure for the SuFEx conjugation and purified by silica gel column chromatography (ethyl acetate/hexanes 1:49 to 1:19) afforded **13** as a pale yellow solid (0.3 g, 46% yield); $R_f$ = 0.42 (20% ethyl acetate in hexane); m.p. = 130–132 °C; $^1$H NMR (500MHz, $CDCl_3$) δ 7.86 (s, 2H), 3.71 – 3.68 (m, 4H), 3.21 – 3.18 (m, 4H), 2.87 – 2.84 (m, 4H), 2.43 (ddd, *J* = 25.8, 17.6, 8.3 Hz, 4H); $^{13}$C NMR (125 MHz, $CDCl_3$) δ 147.9, 133.2, 118.5 – 115.9 (m), 111.5 – 110.4 (m), 109.0 – 108.4 (m), 89.5, 52.8, 31.9 (t, *J* (C-$CF_2$) = 22.2 Hz), 25.8, 23.3; HRMS (ESI) m/z: $[M - H]^+$ Calcd for $C_{26}H_{17}F_{26}I_2O_6S_4$ 1300.7582; Found 1300.7574.

**2,5-Diiodo-1,4-phenylene bis(2-(octylthio)ethane-1-sulfonate) (14)**

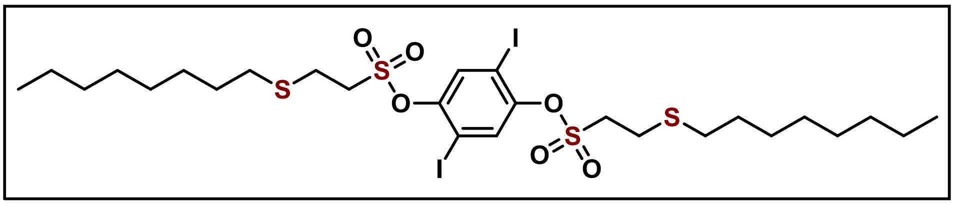

The compound was prepared by following general procedure for the SuFEx conjugation and purified by silica gel column chromatography (ethyl acetate/hexanes 1:49 to 1:19) afforded **14** as a white solid (0.26 g, 62% yield); $R_f$ = 0.65 (20% ethyl acetate in hexane); m.p. = 87–89 °C; $^1$H NMR (500MHz, $CDCl_3$) δ 7.85 (s, 2H), 3.68 – 3.65 (m, 4H), 3.14 – 3.11 (m, 4H), 2.61 – 2.58 (m, 4H), 1.62 (dt, *J* = 15.0, 7.4 Hz, 4H), 1.39 (dt, *J* = 14.4, 7.0 Hz, 4H), 1.32 – 1.27 (m, 16H), 0.88 (t, *J* = 6.9 Hz, 6H); $^{13}$C NMR (125 MHz, $CDCl_3$) δ 147.9, 133.1, 89.5, 53.2, 32.6, 31.9, 29.6, 29.31, 29.29, 28.9, 25.4, 22.8, 14.2; HRMS (ESI) m/z: $[M + H]^+$ Calcd for $C_{26}H_{45}I_2O_6S_4$ 835.0188; Found 835.0196.

**SP-1**

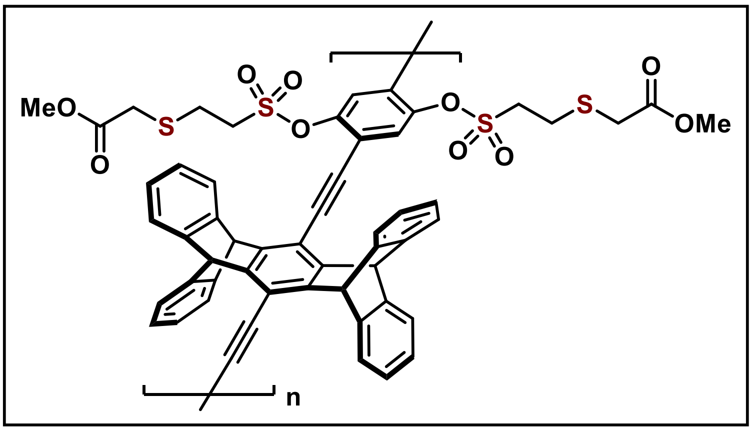

The compound was prepared by following Sonogashira polymerization for the SP polymers and purified by reprecipitation afforded **SP-1** as a yellow solid (25 mg, 13% yield); $^1$H NMR (500MHz, $CDCl_3$) δ 7.70 (br, 10H), 7.00 (br, 8H), 5.87 (br, 4H), 3.64 (br, 6H), 0.85 (br, 12H); $M_n$ = 3803, PDI = 1.3; $\lambda_{max}^{abs}$ = 350 nm, $\lambda_{max}^{fl}$ = 419 nm; quantum yield, $\phi_{fl}$ = 39%.

**SP-2**

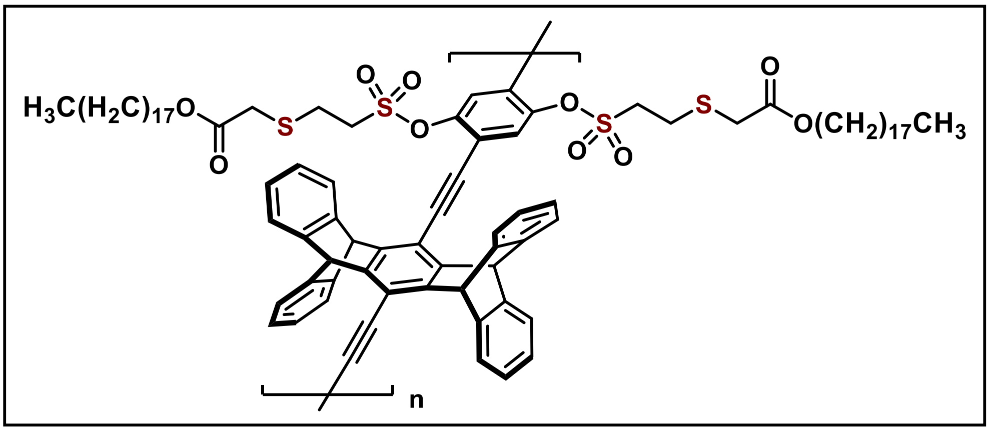


The compound was prepared by following Sonogashira polymerization for the SP polymers and purified by reprecipitation afforded **SP-2** as a yellow solid (96 mg, 33% yield); $^{1}$H NMR (500MHz, $CDCl_3$) δ 7.78 (br, 10H), 7.03 (br, 8H), 5.89 (br, 4H), 4.05 (br, 4H), 3.15 (br, 8H), 1.00 (br, 42H); $M_n$ = 5167, PDI = 1.4; $\lambda_{max}^{abs}$ = 356 nm, $\lambda_{max}^{fl}$ = 420 nm; quantum yield, $\phi_{fl}$ = 18%.

**SP-3**

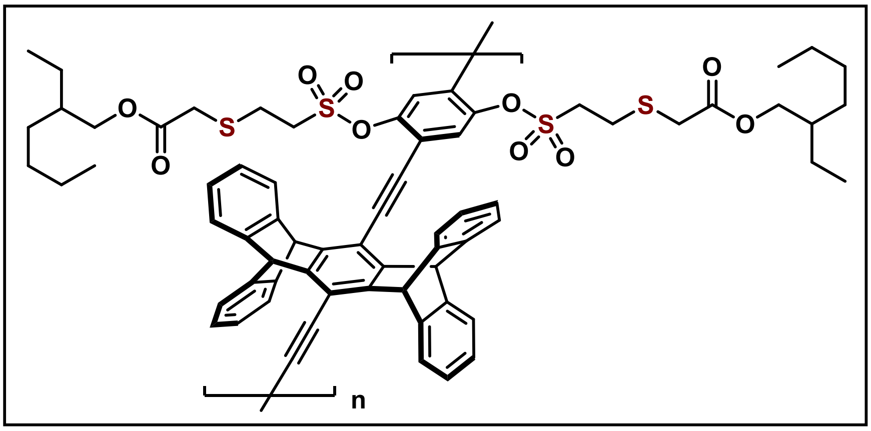


The compound was prepared by following Sonogashira polymerization for the SP polymers and purified by reprecipitation afforded **SP-3** as a yellow solid (136 mg, 57% yield); $^{1}$H NMR (500MHz, $CDCl_3$) δ 8.16 (br, 2H), 7.46 (br, 8H), 7.10 (br, 8H), 5.87 (br, 4H), 3.49 (br, 12H), 0.94 (br, 30H); $M_n$ = 13622, PDI = 2.1; $\lambda_{max}^{abs}$ = 357 nm, $\lambda_{max}^{fl}$ = 418 nm; quantum yield, $\phi_{fl}$ = 31%.

**SP-4**

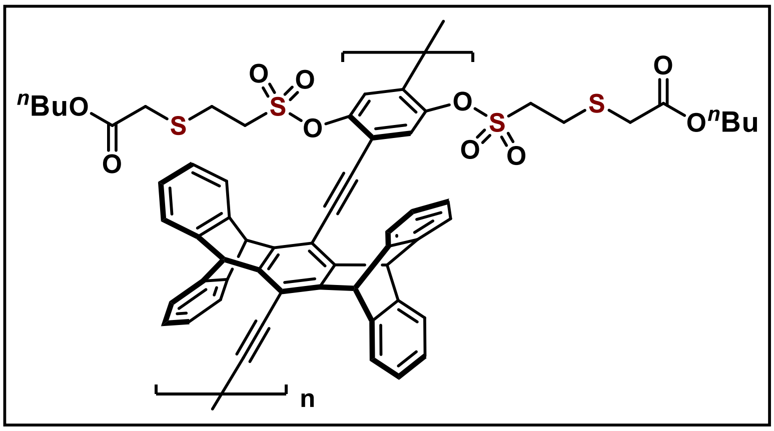


The compound was prepared by following Sonogashira polymerization for the SP polymers and purified by reprecipitation afforded **SP-4** as a yellow solid (32 mg, 15% yield); $^{1}$H NMR (500MHz, $CDCl_3$) δ 7.79 (br, 10H), 7.06 (br, 8H), 5.90 (br, 4H), 4.06 (br, 4H), 3.26 (br, 8H), 1.25 (br, 18H); $M_n$ = 5719, PDI = 1.4; $\lambda_{max}^{abs}$ = 355 nm, $\lambda_{max}^{fl}$ = 417 nm; quantum yield, $\phi_{fl}$ = 25%.

**SP-5**

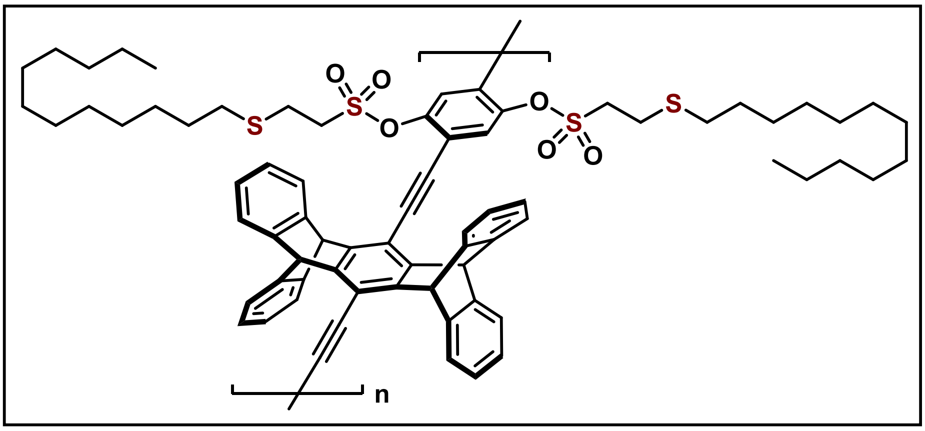


The compound was prepared by following Sonogashira polymerization for the SP polymers and purified by reprecipitation afforded **SP-5** as a yellow solid (134 mg, 57% yield); $^{1}$H NMR (500MHz, $CDCl_3$) δ 8.22 (br, 2H), 7.53 (br, 8H), 7.07 (br, 8H), 6.00 (br, 4H), 3.69 (br, 4H), 2.83 (br, 4H), 1.13 (br, 50H); $M_n$ = 16967, PDI = 1.6; $\lambda_{max}^{abs}$ = 357 nm, $\lambda_{max}^{fl}$ = 417 nm; quantum yield, $\phi_{fl}$ = 67%.

**SP-6**

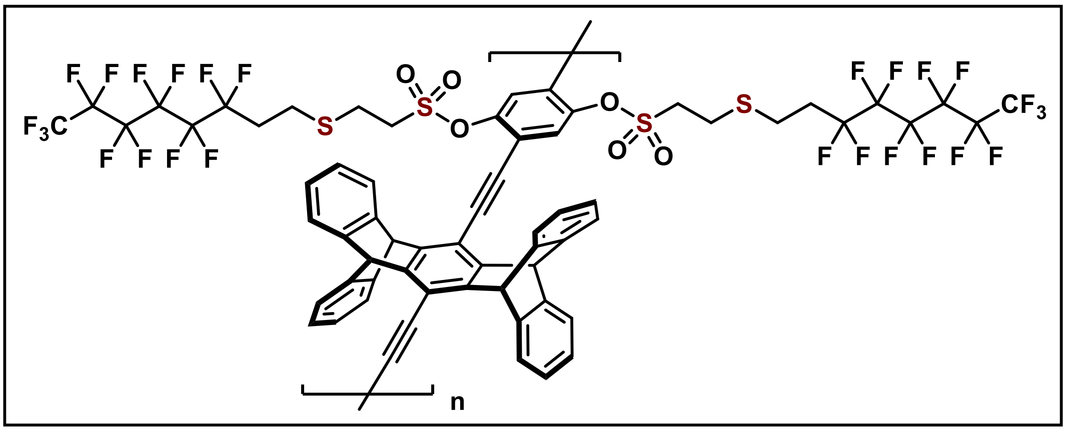


The compound was prepared by following Sonogashira polymerization for the SP polymers and purified by reprecipitation afforded **SP-6** as a yellow solid (118 mg, 39% yield); $^{1}$H NMR (500MHz, $CDCl_3$) δ 7.79 (br, 10H), 7.08 (br, 8H), 5.96 (br, 4H), 3.16 (br, 4H), 1.55 (br, 4H), 1.18 (br, 4H), 0.79 (br, 4H); $M_n$ = 12824, PDI = 1.8; $\lambda_{max}^{abs}$ = 355 nm, $\lambda_{max}^{fl}$ = 424 nm; quantum yield, $\phi_{fl}$ = 31%.

**SP-7**

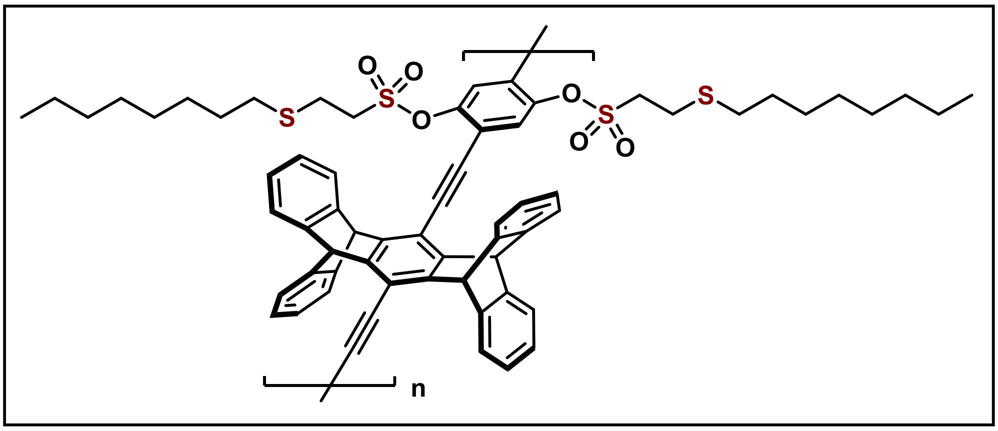


The compound was prepared by following Sonogashira polymerization for the SP polymers and purified by reprecipitation afforded **SP-7** as a yellow solid (159 mg, 75% yield); $^{1}$H NMR (500MHz, $CDCl_3$) δ 8.19 (br, 2H), 7.49 (br, 8H), 7.07 (br, 8H), 5.97 (br, 4H), 3.64 (br, 4H), 2.94 (br, 4H), 1.26 (br, 34H); $M_n$ = 17550, PDI = 1.7; $\lambda_{max}^{abs}$ = 358 nm, $\lambda_{max}^{fl}$ = 420 nm; quantum yield, $\phi_{fl}$ = 74%.

## 6. Analytical data of polymers

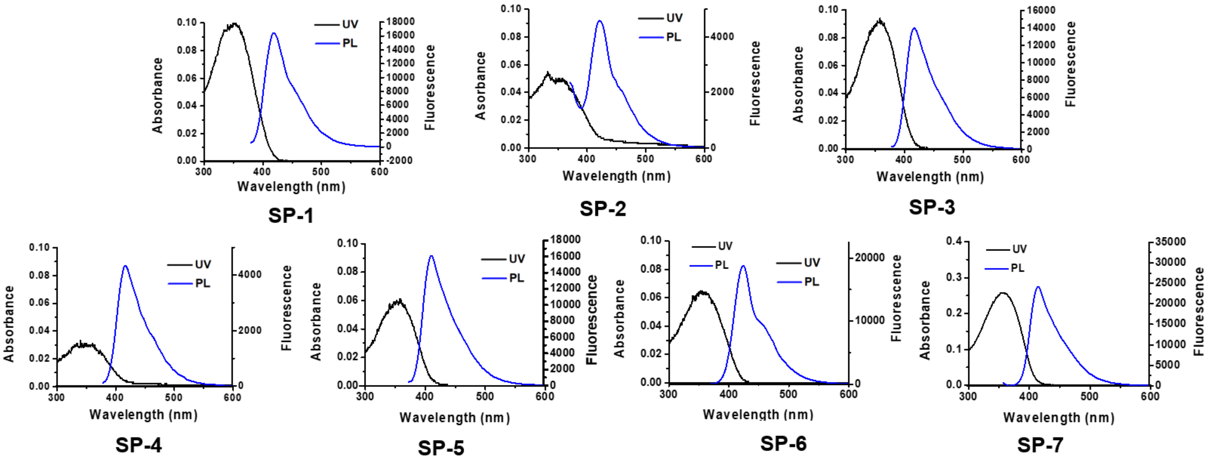


**Figure S1.** UV (absorption) and PL (emission) data : solution in $CHCl_3$ (0.5 mg/mL)

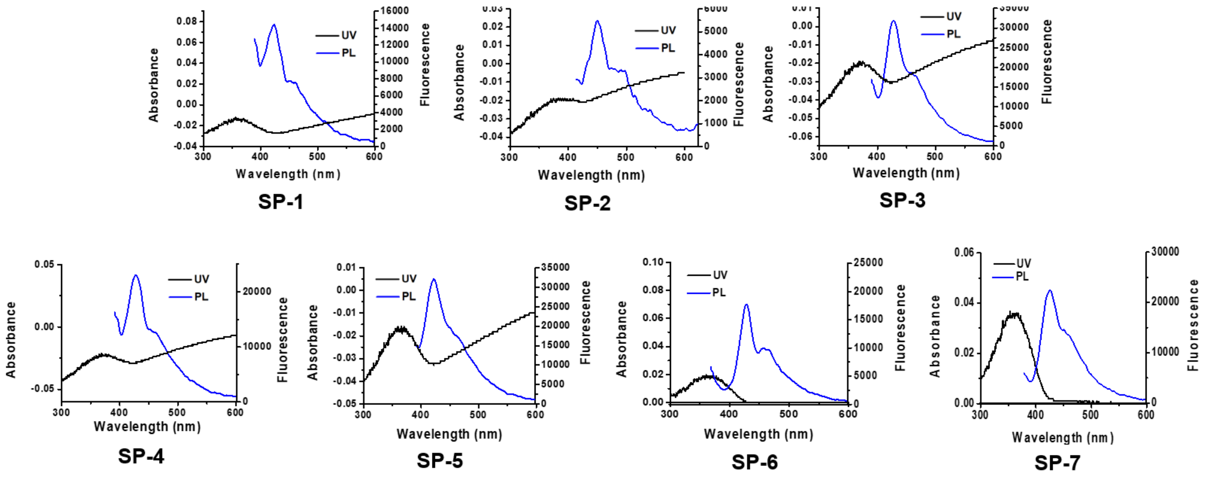


**Figure S2.** UV (absorption) and PL (emission) data : spin-cast thin film

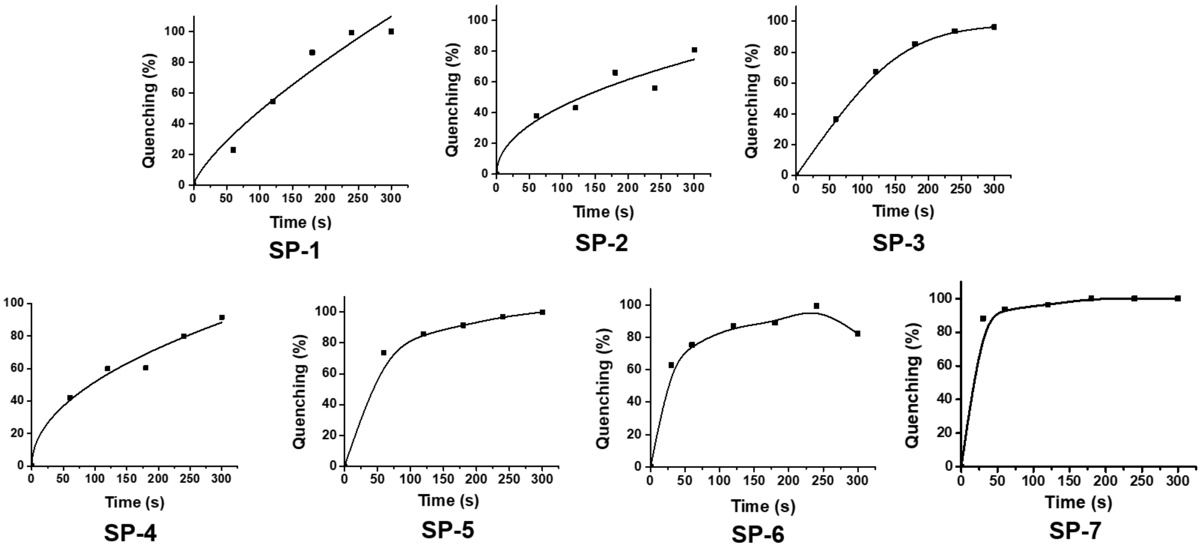


**Figure S3.** 2,4-DNT fluorescence quenching property of polymers

**Table S1.** Fluorescence quenching over time

| | Quenching (%) | | | | | | |
|---|---|---|---|---|---|---|---|
| Tims (s) | 0 | 30 | 60 | 120 | 180 | 240 | 300 |
| **P-1** | 0 | 34.2 | 61.3 | - | 69.5 | - | 67.7 |
| **SP-1** | 0 | - | 22.9 | 54.3 | 86.3 | 99.4 | >99 |
| **SP-2** | 0 | - | 37.9 | 43.2 | 66.1 | 56.0 | 80.9 |
| **SP-3** | 0 | - | 36.5 | 67.2 | 85.4 | 93.7 | 96.4 |
| **SP-4** | 0 | - | 42.2 | 59.8 | 60.3 | 79.9 | 91.4 |
| **SP-5** | 0 | - | 73.7 | 85.9 | 91.4 | 97.0 | >99 |
| **SP-6** | 0 | 62.9 | 75.4 | 86.9 | 88.9 | 99.3 | 82.3 |
| **SP-7** | 0 | 88.2 | 93.6 | 96.3 | >99 | >99 | >99 |

## 7. Photoluminescence measurement

| | PL intensity / counts | | Lifetime / ns | |
|---|---|---|---|---|
| **[Q] / mM** | **P-1** | **SP-7** | **P-1** | **SP-7** |
| 0 | 10221.7 | 5973.7 | 0.450 | 0.209 |
| 5 | 8609.9 | 2023.1 | 0.432 | 0.169 |
| 10 | 6875.0 | 1253.1 | 0.407 | 0.148 |
| 15 | 5744.3 | 1048.4 | 0.390 | 0.136 |
| 20 | 4989.5 | 735.9 | 0.365 | 0.120 |
| 25 | 4020.0 | 611.6 | 0.347 | 0.098 |
| 30 | 3561.6 | 503.1 | 0.333 | 0.086 |

**Table S2. Summary of photoluminescence (PL) intensities and fluorescence lifetimes of polymers P-1 and SP-7 obtained from steady-state and time-resolved PL measurements at different concentrations of the quencher 2,4-DNT.**

Table S2 presents the photoluminescence (PL) intensities and fluorescence lifetimes of polymers P-1 and SP-7 at various concentrations of the quencher 2,4-DNT, obtained from steady-state and time-resolved PL measurements. The data in this table were used to plot Figures 5A and 5B, which show the changes in PL intensity and lifetime, respectively, at a constant polymer concentration of 10 μM. These values were subsequently utilized for the Stern–Volmer analysis shown in Figure 5C.[6]

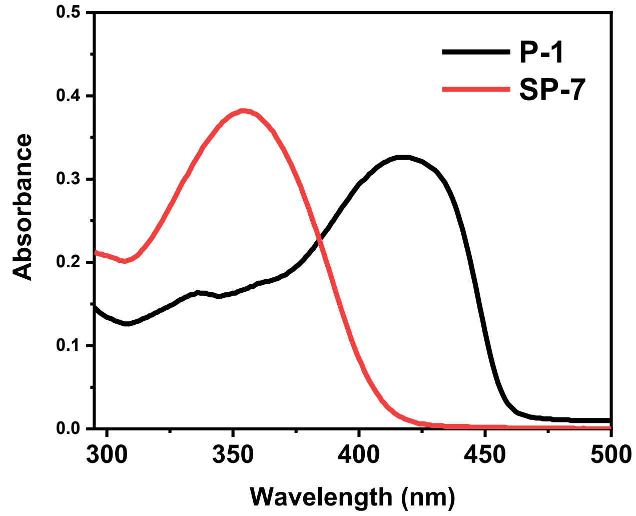


**Figure S4. UV–Vis absorption spectrum of polymer solution.**

The UV–Vis absorption spectra of P-1 (black) and SP-7 (red) polymer solutions were measured in chloroform at a concentration of 10 μM.

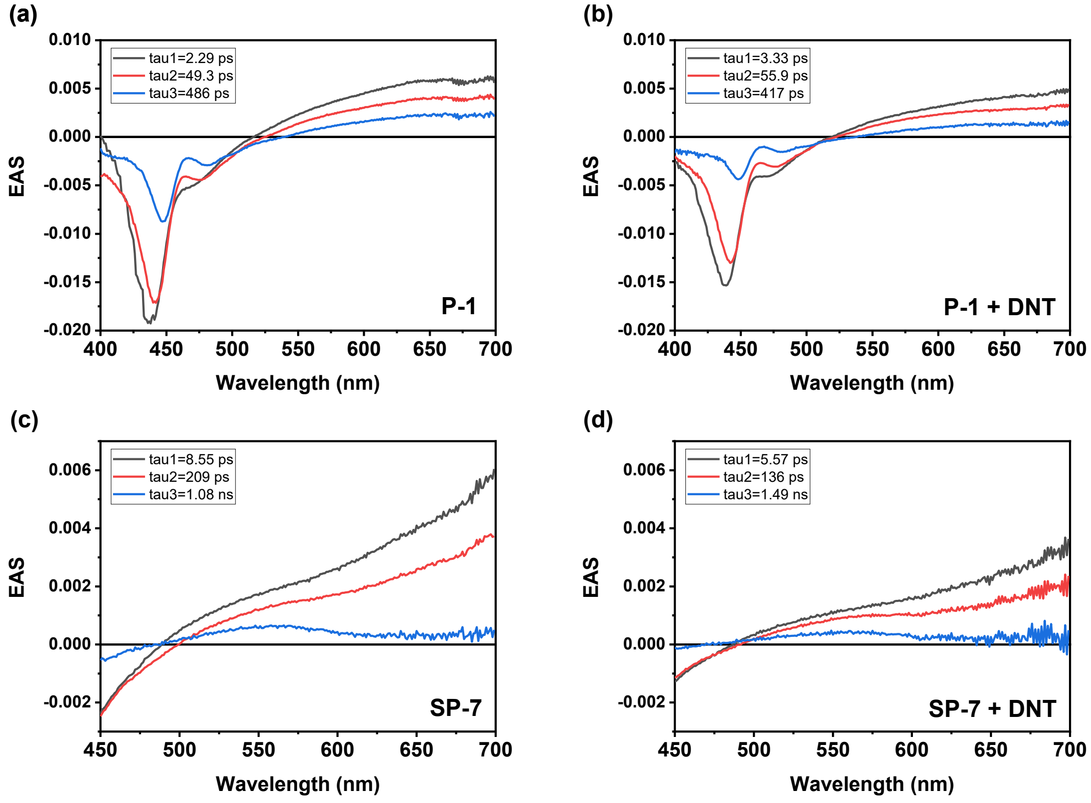


**Figure S5. Evolution-associated spectra (EAS) obtained from global fitting of the transient absorption data: (a) Polymer P-1, (b) Polymer P-1 with quencher DNT, (c) Polymer SP-7, and (d) Polymer SP-7 with quencher DNT.[7]**

P-1 shows three time constants of approximately 2 ps, 49 ps, and 486 ps were obtained. The first and second components (2 and 49 ps) can be assigned to vibrational/structural relaxations inducing the spectral redshifts for the SE signals while the third time component (486 ps) is the $S_1$ state lifetime which closely matches the fluorescence lifetime of P-1. SP-7 also shows three time constants of 9 ps, 209 ps, and 1 ns where the first component of 9 ps is assigned to structural relaxation which also governs spectral redshifts of the SE signals, and the time component of 209 ps is assigned to the $S_1$ state lifetime which matches the fluorescence lifetime. The third component in SP-7 would be a triplet state lifetime because the SE signals are almost negligible. Examining the EAS spectra of the two polymers with DNT added, we find that the amplitude of the fastest component is lower compared to when DNT is absent. The decrease in initial signal intensity indicates that static quenching occurs, where the polymer and quencher form a complex in the ground state before rapid dynamic processes occur, reducing the number of emissive molecules capable of transition.[8] A decrease in the time constants assigned to fluorescence decay was also observed: from 486 ps to 417 ps in P-1, and from 209 ps to 136 ps in SP-7, confirming that this occurs due to the influence of dynamic quenching. Comparing the 136 ps spectrum of SP-7 with added DNT, corresponding to fluorescence decay, to the 209 ps spectrum of SP-7, we notice a slightly increased signal around 560 nm. This result confirms that fluorescence quenching occurs via an electron transfer mechanism.[9]

## 8. DFT calculations

### A. HOMO and LUMO structures of monomeric analogues

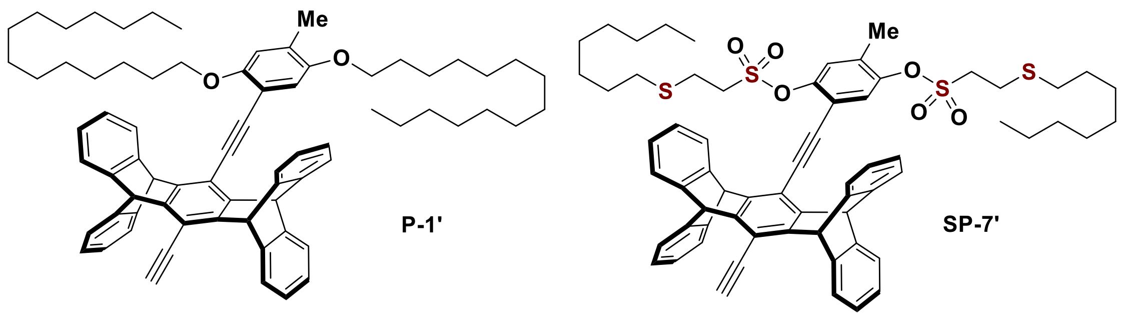


### B. TD-DFT data of P-1', SP-7'

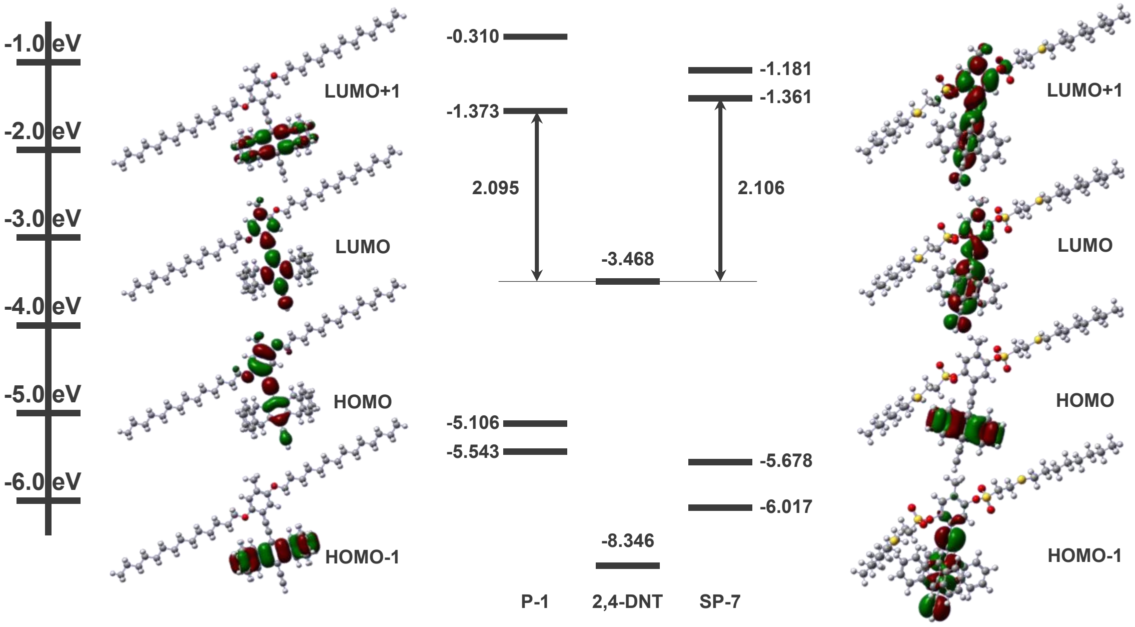


### C. Calculated HOMO and LUMO energies

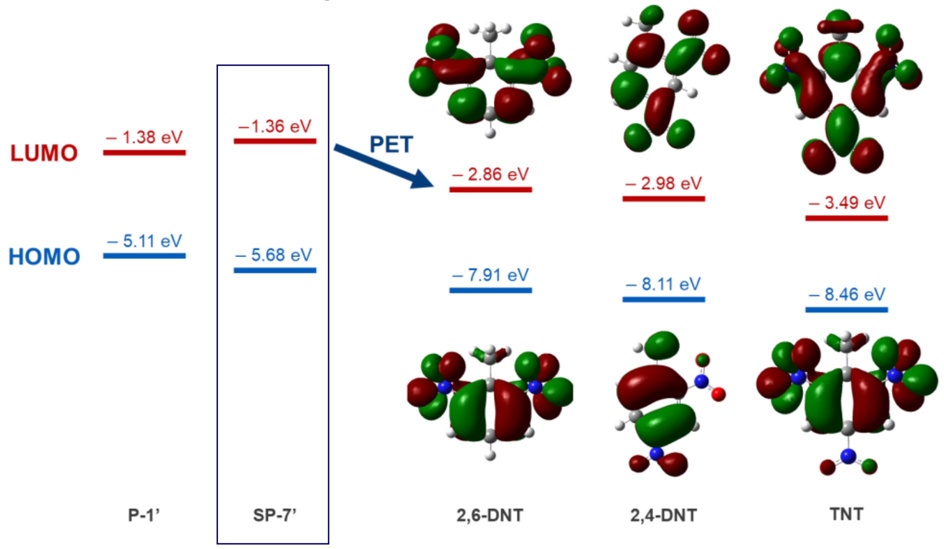

**Figure S6. DFT study for the fluorescence quenching results of SP series polymers using 2,4-DNT and TNT vapors.**

We simplified the end groups of SP-7 to hydrogen and methyl groups, designated as SP-7', and calculated the structures and electron distributions of both the Lowest Unoccupied Molecular Orbital (LUMO) and the Highest Occupied Molecular Orbital (HOMO). The results delivered values of –1.36 eV for LUMO and –5.68 eV for HOMO. In comparison, the analogous structure of P-1' demonstrated LUMO and HOMO values of –1.38 eV and –5.11 eV, respectively, indicating relatively lower values for SP-7'. Considering the LUMO values for DNTs and TNT, which range from –2.86 eV to –3.49 eV, the structure of SP-7 can be explained to more effectively undergo photoinduced electron transfer (PET).

| No. | Wavelength (nm) | Osc. Strength | Major contributions |
|---|---|---|---|
| 1 | 6427.4 | 0.0021 | HOMO(B)->LUMO(B) (99%) |
| 2 | 2200.6 | 0.0003 | H-1(B)->LUMO(B) (100%) |
| 3 | 1222.1 | 0.0179 | H-2(B)->LUMO(B) (94%) |
| 4 | 1213.2 | 0.002 | H-3(B)->LUMO(B) (94%) |
| 5 | 1192.7 | 0 | H-4(B)->LUMO(B) (98%) |
| 6 | 1142.5 | 0.1708 | H-5(B)->LUMO(B) (93%) |
| 7 | 1138.0 | 0 | H-6(B)->LUMO(B) (99%) |
| 8 | 1088.8 | 0.0007 | H-7(B)->LUMO(B) (99%) |
| 9 | 1022.9 | 0.0026 | H-8(B)->LUMO(B) (100%) |
| 10 | 857.8 | 0.0014 | H-11(B)->LUMO(B) (98%) |
| 11 | 803.8 | 0 | H-9(B)->LUMO(B) (100%) |
| 12 | 780.2 | 0 | H-10(B)->LUMO(B) (100%) |
| 13 | 663.7 | 0.0803 | H-18(B)->LUMO(B) (90%) |
| 14 | 578.2 | 0 | H-12(B)->LUMO(B) (100%) |
| 15 | 568.3 | 0 | H-13(B)->LUMO(B) (100%) |
| 16 | 557.2 | 0 | H-30(B)->LUMO(B) (57%), H-24(B)->LUMO(B) (22%), H-14(B)->LUMO(B) (19%) |
| 17 | 556.6 | 0 | H-30(B)->LUMO(B) (12%), H-14(B)->LUMO(B) (81%) |
| 18 | 547.1 | 0 | H-24(B)->LUMO(B) (20%), H-15(B)->LUMO(B) (73%) |
| 19 | 546.6 | 0 | H-30(B)->LUMO(B) (21%), H-24(B)->LUMO(B) (50%), H-15(B)->LUMO(B) (26%) |

| | | | |
|---|---|---|---|
| 20 | 540.8 | 0 | H-16(B)->LUMO(B) (99%) |

**Table S3. Time-dependent density functional theory (TD-DFT) calculated electronic transitions for the P-1 radical cation using the uB3LYP/6-31G(d) basis set.** Calculations were performed with 20 excited states.

| No. | Wavelength (nm) | Osc. Strength | Major contributions |
|---|---|---|---|
| 1 | 139308.1 | 0.0001 | HOMO(B)->LUMO(B) (178%) |
| 2 | 13579.9 | 0 | H-1(B)->LUMO(B) (100%) |
| 3 | 5856.6 | 0.0005 | H-2(B)->LUMO(B) (89%) |
| 4 | 1575.8 | 0.18 | H-3(B)->LUMO(B) (88%), H-2(B)->LUMO(B) (10%) |
| 5 | 1323.2 | 0.021 | H-6(B)->LUMO(B) (24%), H-4(B)->LUMO(B) (65%) |
| 6 | 1187.1 | 0.0001 | H-8(B)->LUMO(B) (82%), H-7(B)->LUMO(B) (12%) |
| 7 | 1175.2 | 0.0009 | H-11(B)->LUMO(B) (30%), H-8(B)->LUMO(B) (11%), H-7(B)->LUMO(B) (44%) |
| 8 | 1150.9 | 0.0006 | H-11(B)->LUMO(B) (33%), H-7(B)->LUMO(B) (43%), H-6(B)->LUMO(B) (15%) |
| 9 | 1076.7 | 0.0781 | H-5(B)->LUMO(B) (88%) |
| 10 | 1068.3 | 0.0103 | H-13(B)->LUMO(B) (85%), H-5(B)->LUMO(B) (10%) |
| 11 | 1006.5 | 0.0031 | H-11(B)->LUMO(B) (29%), H-6(B)->LUMO(B) (50%), H-4(B)->LUMO(B) (19%) |
| 12 | 959.3 | 0 | H-10(B)->LUMO(B) (78%), H-9(B)->LUMO(B) (17%) |
| 13 | 906.8 | 0.002 | H-14(B)->LUMO(B) (99%) |
| 14 | 777.5 | 0 | H-10(B)->LUMO(B) (17%), H-9(B)->LUMO(B) (82%) |
| 15 | 751.4 | 0 | H-12(B)->LUMO(B) (100%) |
| 16 | 656.5 | 0.0029 | H-23(B)->LUMO(B) (15%), H-20(B)->LUMO(B) (83%) |
| 17 | 618.2 | 0 | H-15(B)->LUMO(B) (100%) |
| 18 | 603.9 | 0 | H-16(B)->LUMO(B) (100%) |
| 19 | 599.1 | 0.0001 | H-24(B)->LUMO(B) (100%) |
| 20 | 583.8 | 0 | H-17(B)->LUMO(B) (100%) |

**Table S4. Time-dependent density functional theory (TD-DFT) calculated electronic transitions for the SP-7 radical cation using the uB3LYP/6-31G(d) basis set.** Calculations were performed with 20 excited states.

# 9. NMR spectra

$^{1}$H NMR (500 MHz, $CDCl_3$) spectrum of **1**

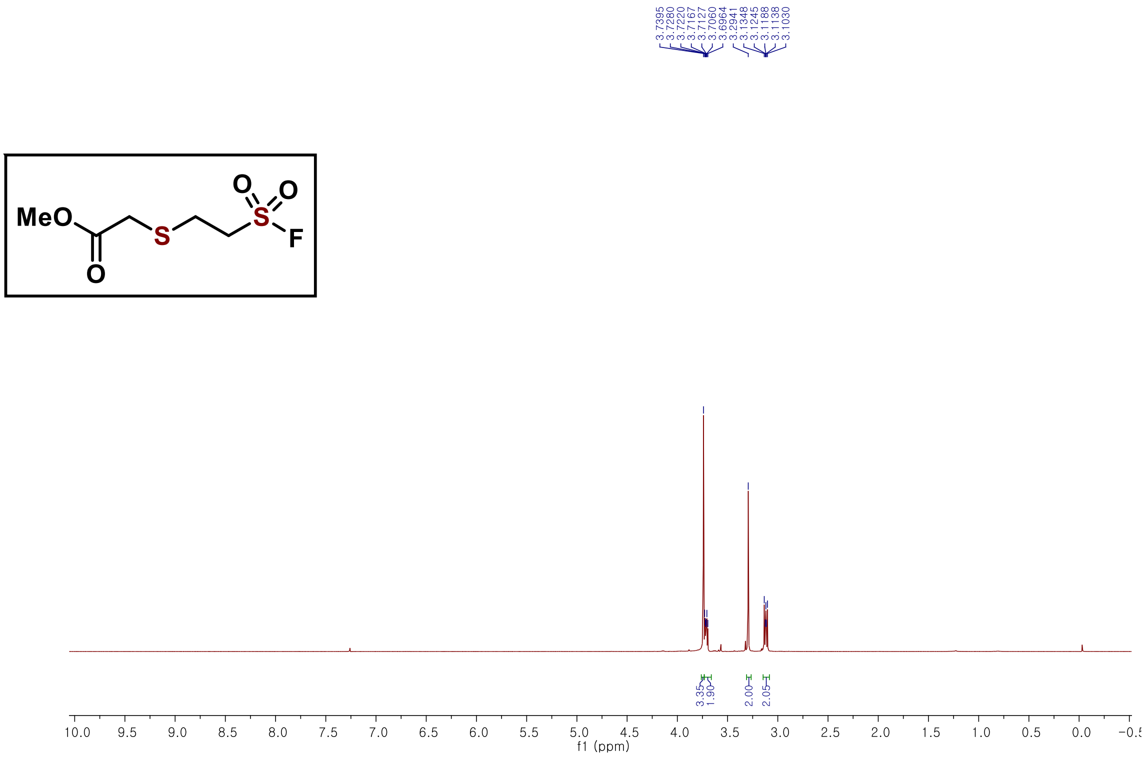


$^{13}$C NMR (125 MHz, $CDCl_3$) spectrum of **2**

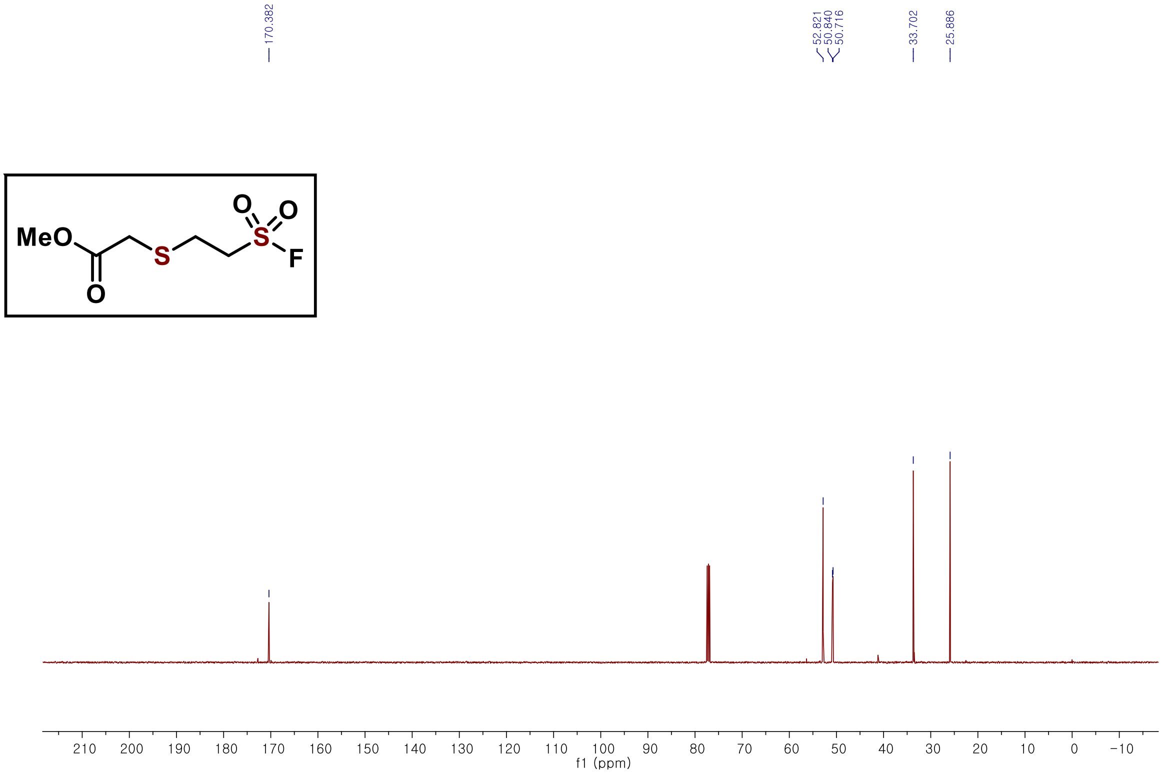

$^{1}H$ NMR (500 MHz, $CDCl_3$) spectrum of **2**

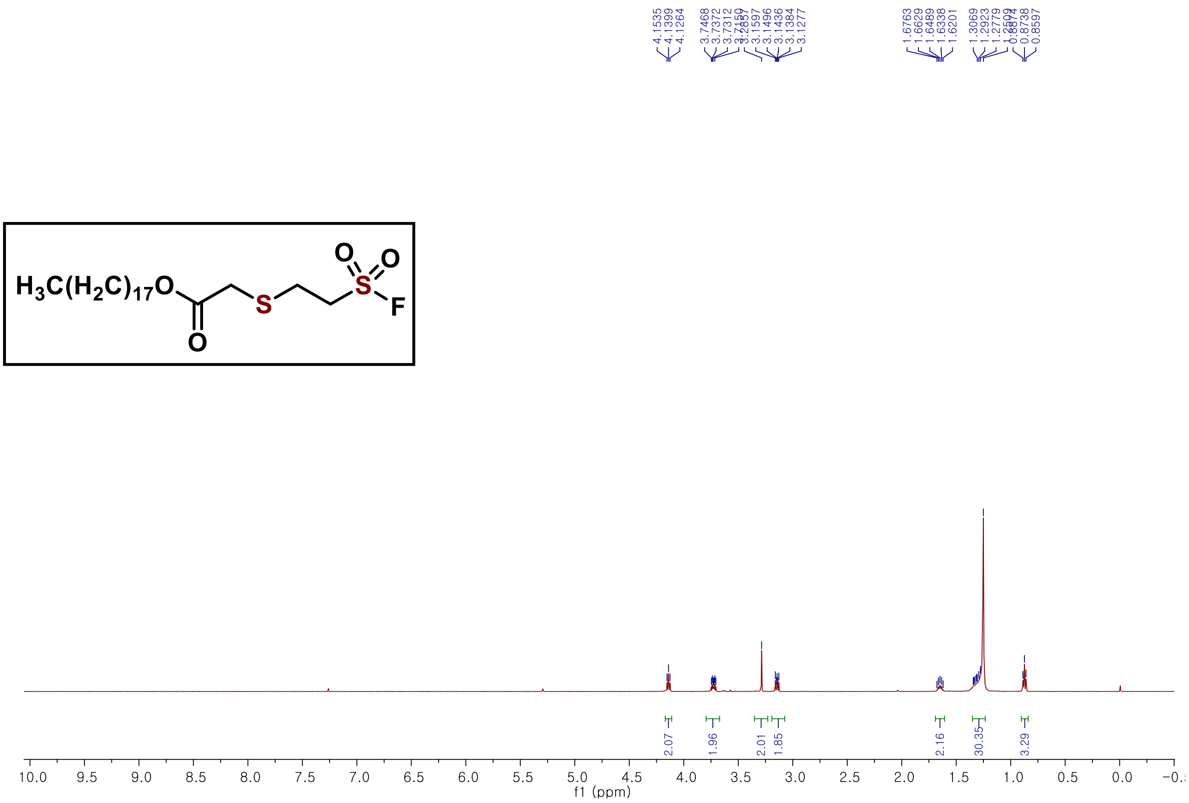


$^{13}C$ NMR (125 MHz, $CDCl_3$) spectrum of **2**

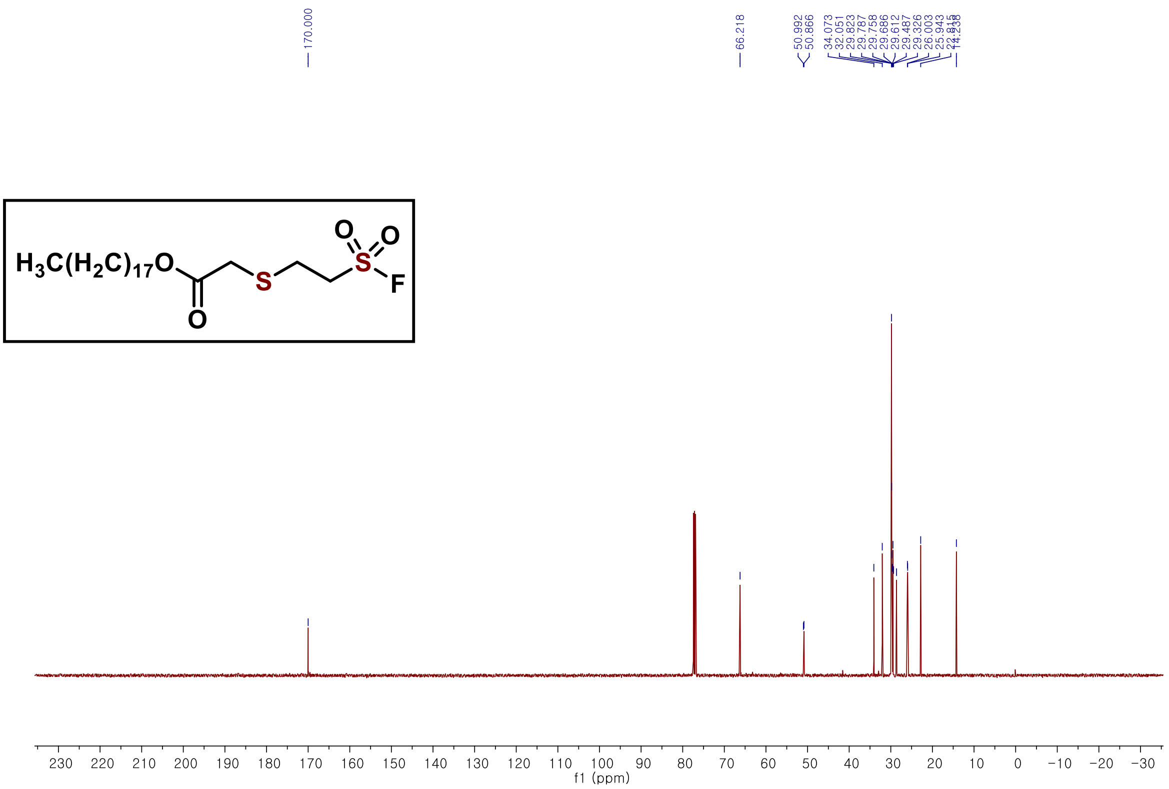

$^{1}$H NMR (500 MHz, $CDCl_3$) spectrum of **3**

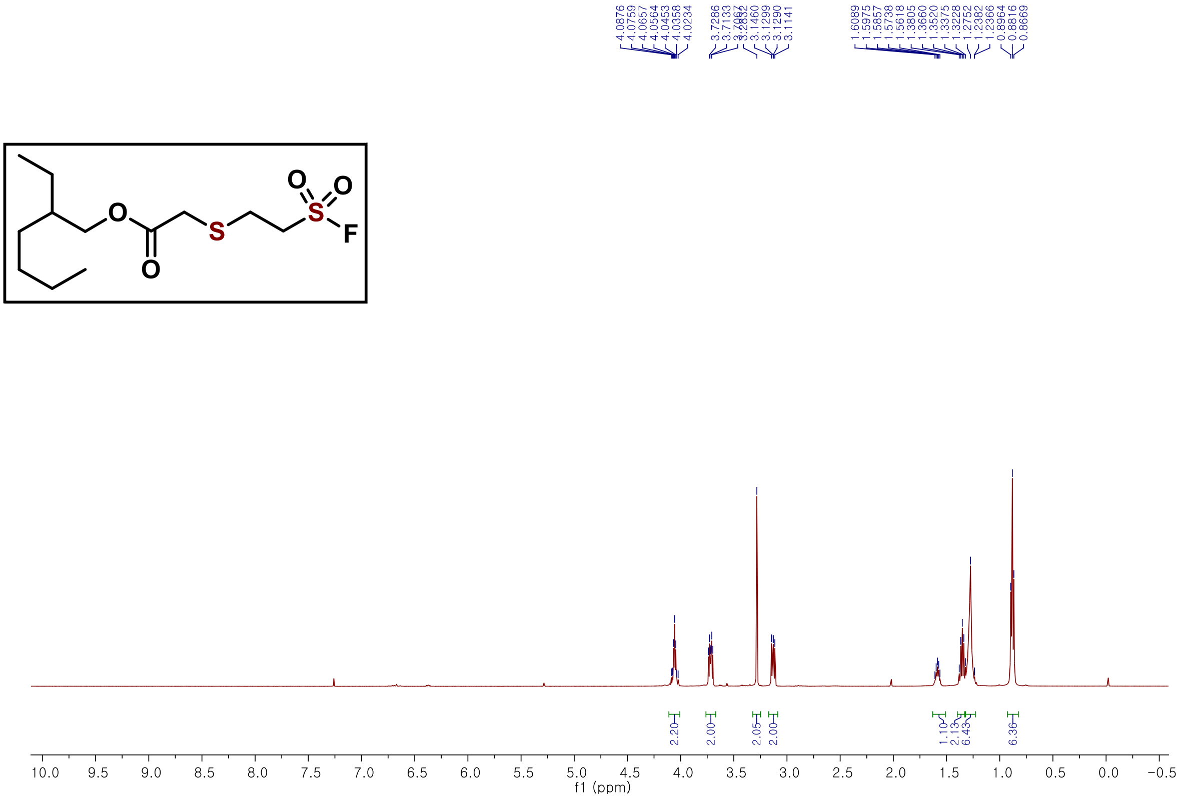


$^{13}$C NMR (125 MHz, $CDCl_3$) spectrum of **3**

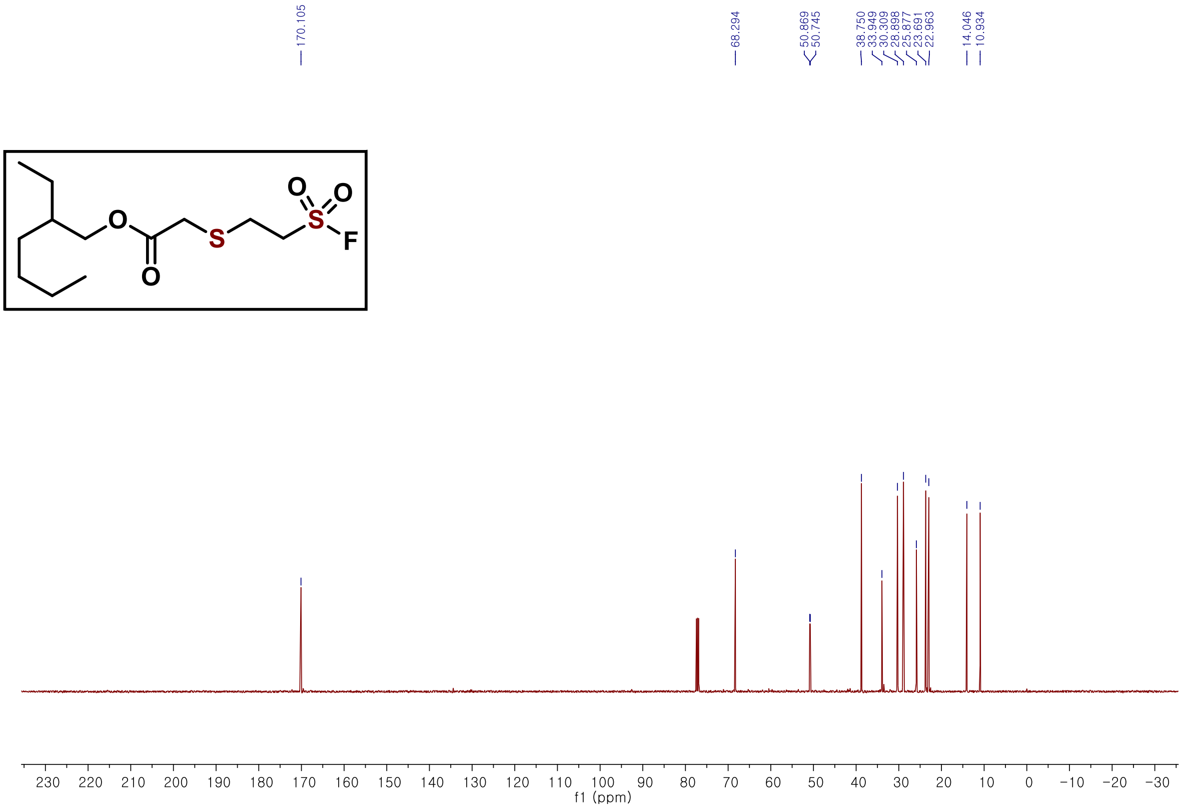

$^{1}H$ NMR (500 MHz, $CDCl_3$) spectrum of **4**

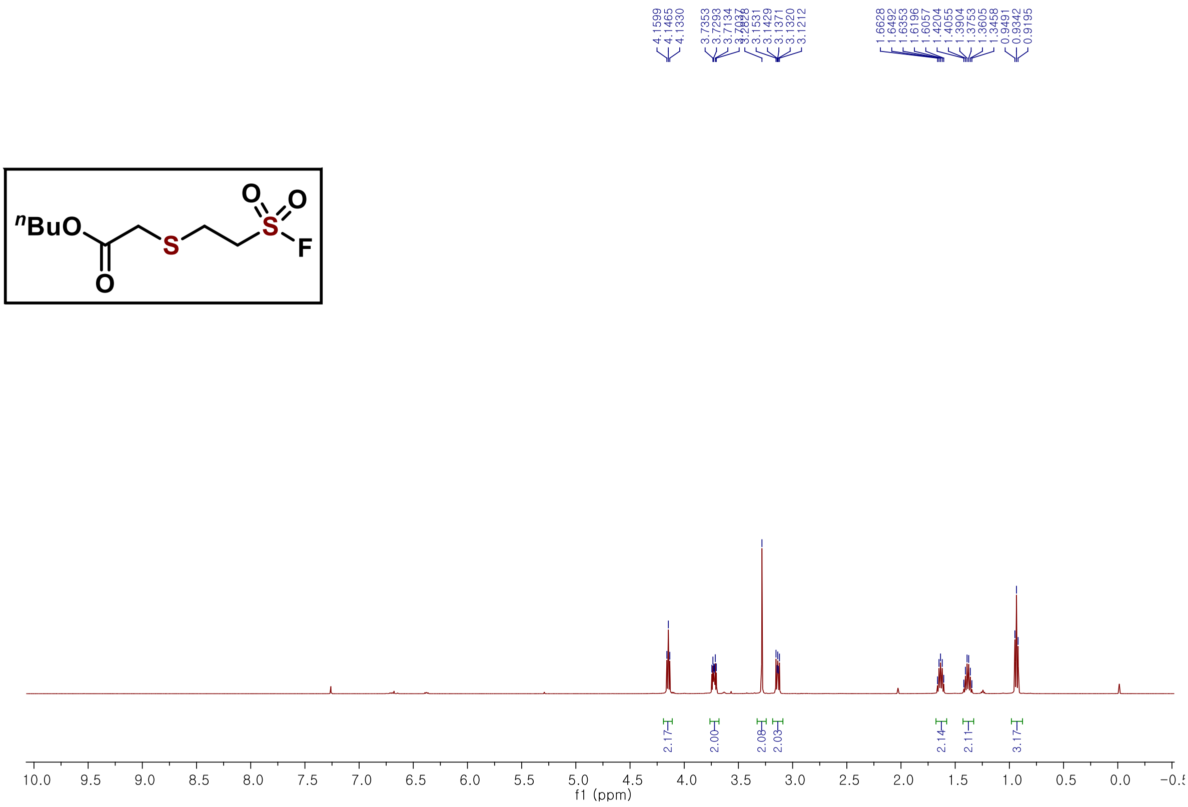


$^{13}C$ NMR (125 MHz, $CDCl_3$) spectrum of **4**

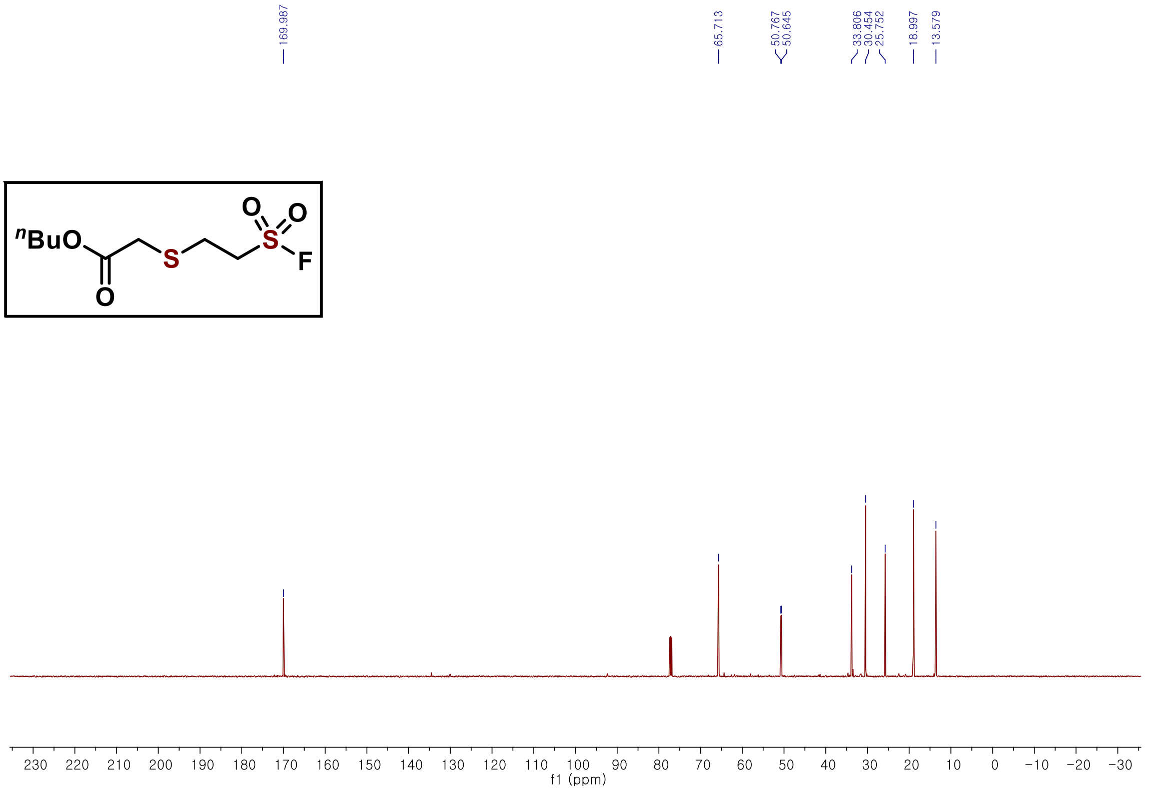

$^{1}H$ NMR (500 MHz, $CDCl_3$) spectrum of **5**

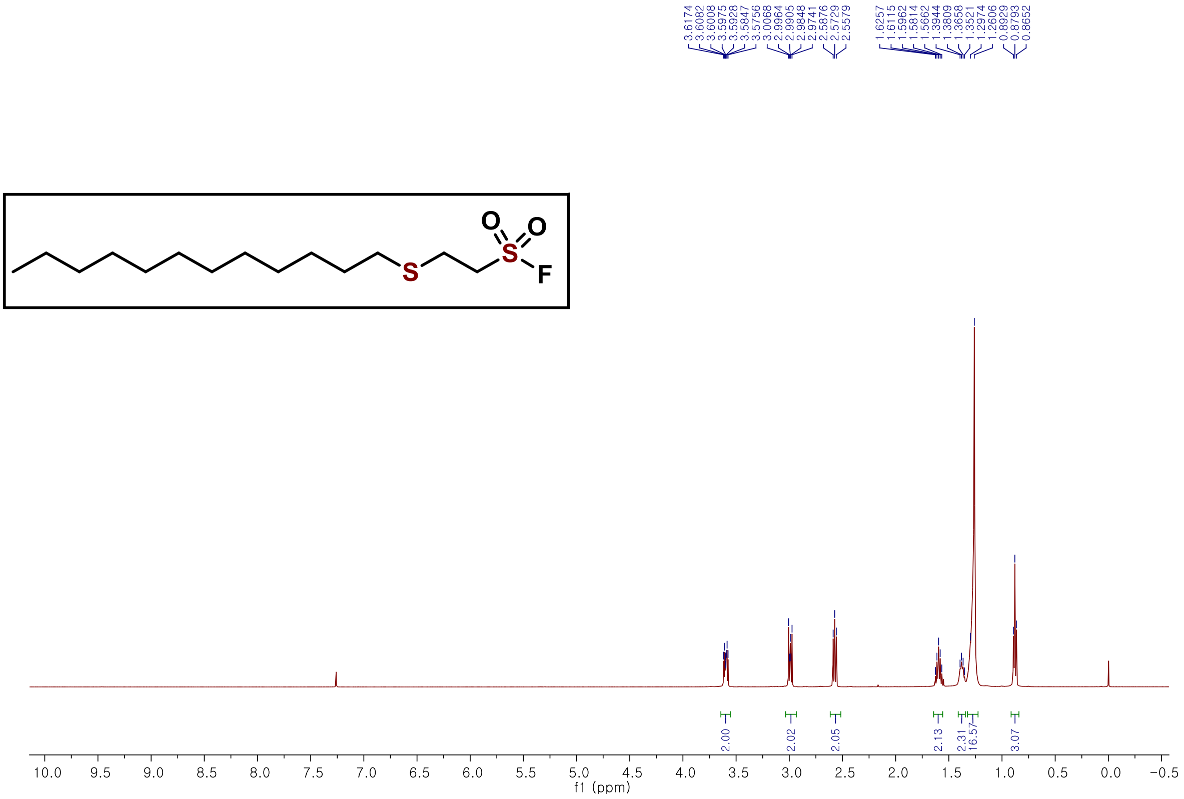


$^{13}C$ NMR (125 MHz, $CDCl_3$) spectrum of **5**

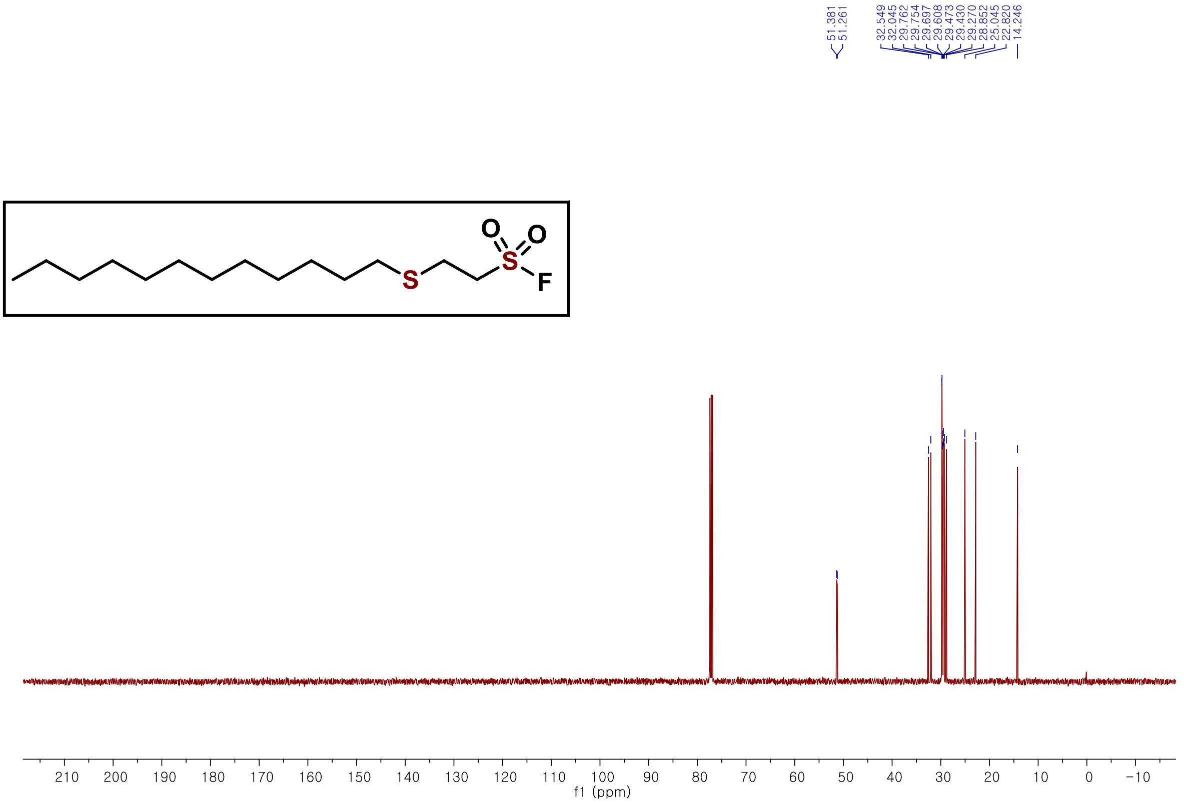

$^{1}$H NMR (500 MHz, CDCl3) spectrum of **6**

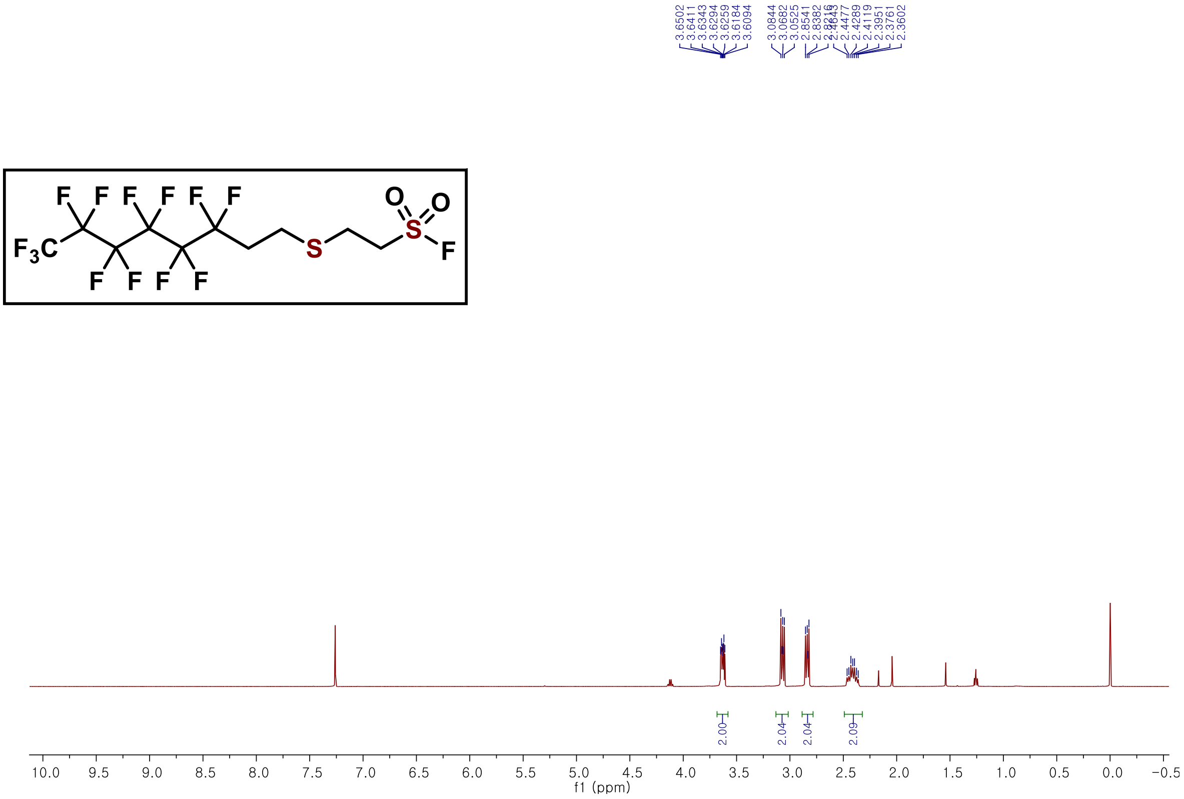


$^{13}$C NMR (125 MHz, CDCl3) spectrum of **6**

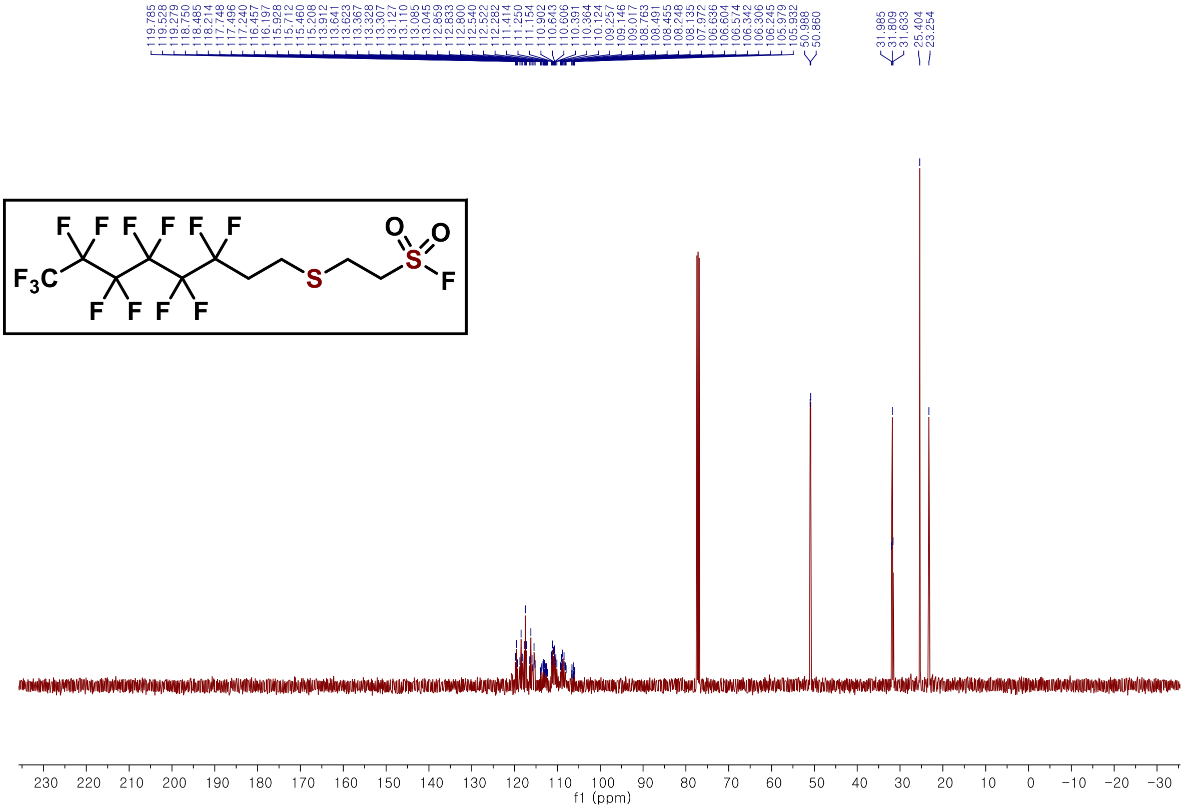

$^{1}H$ NMR (500 MHz, $CDCl_3$) spectrum of **7**

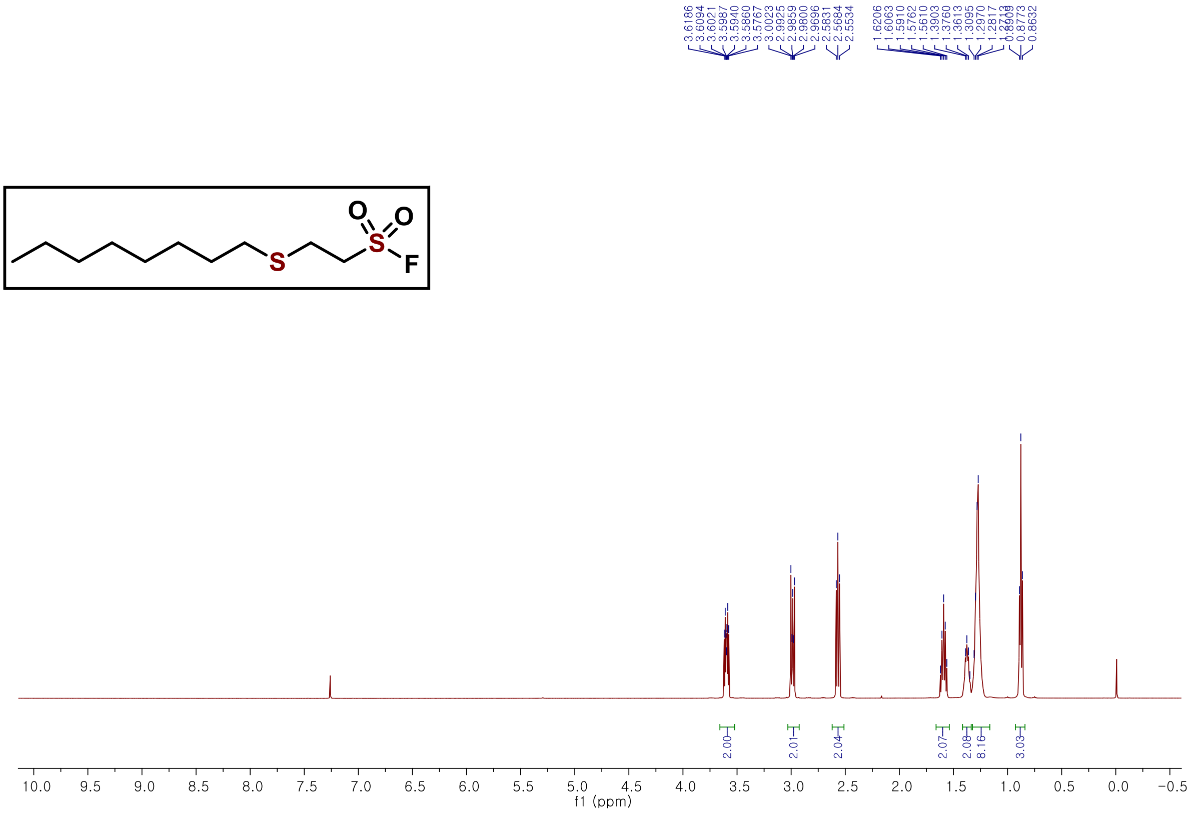


$^{13}C$ NMR (125 MHz, $CDCl_3$) spectrum of **7**

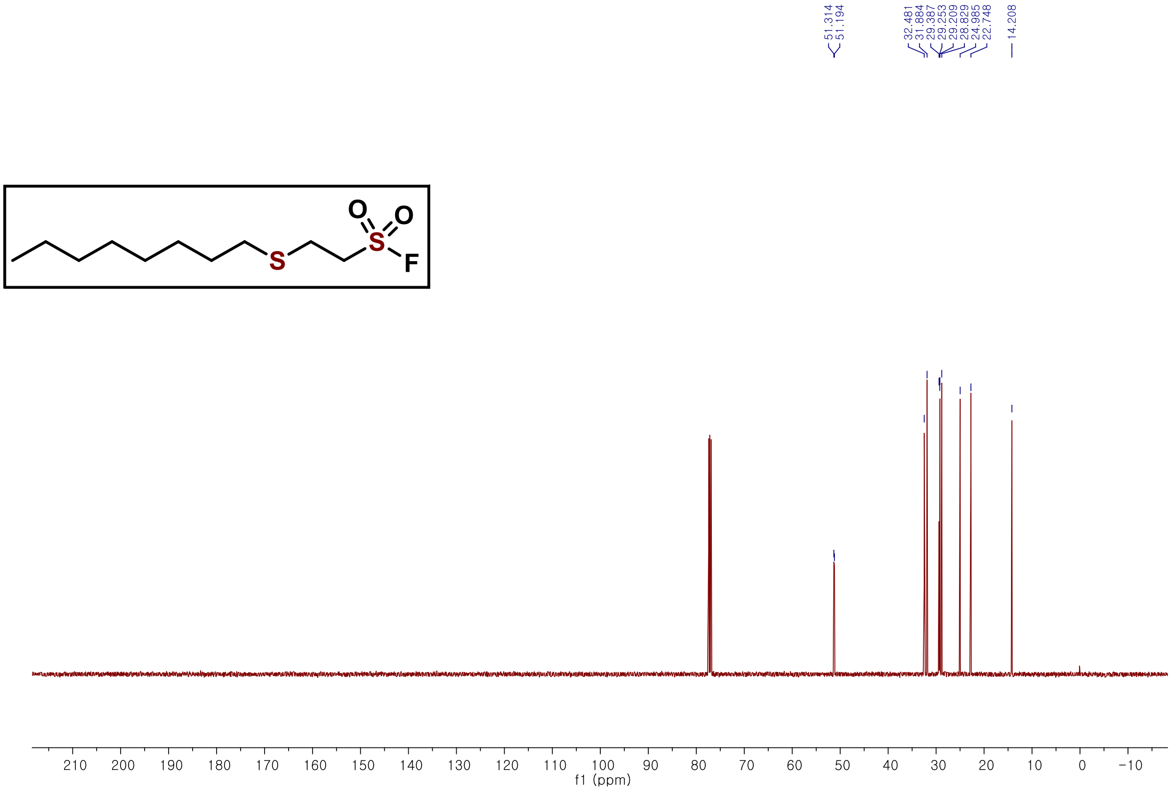

$^{1}$H NMR (500 MHz, $CDCl_3$) spectrum of **8**

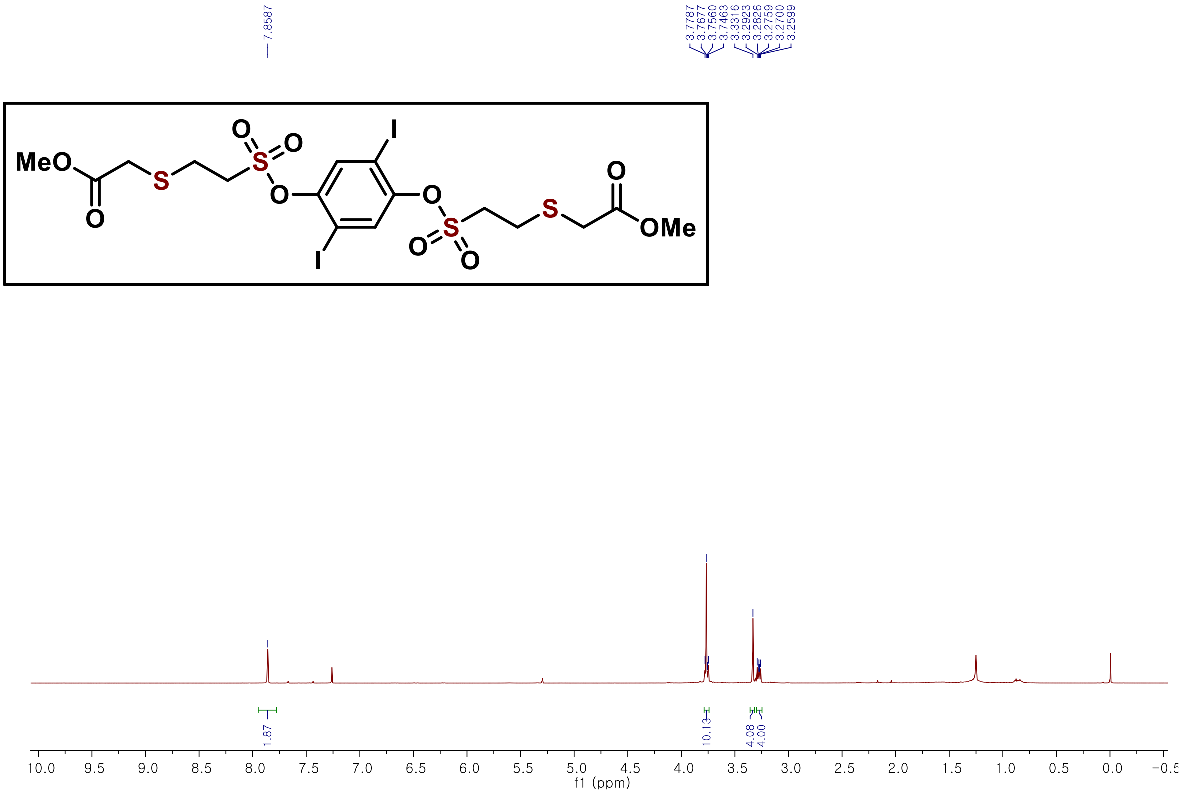


$^{13}$C NMR (125 MHz, $CDCl_3$) spectrum of **8**

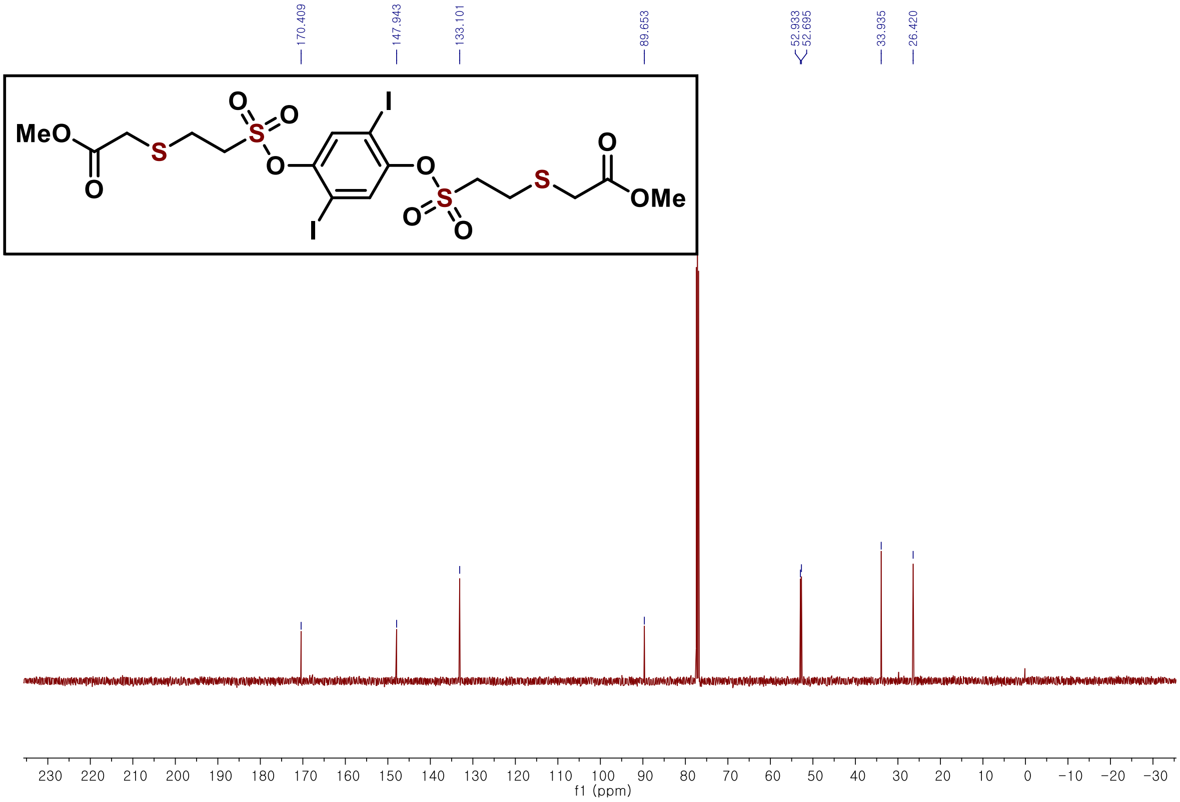

$^{1}H$ NMR (500 MHz, $CDCl_3$) spectrum of **9**

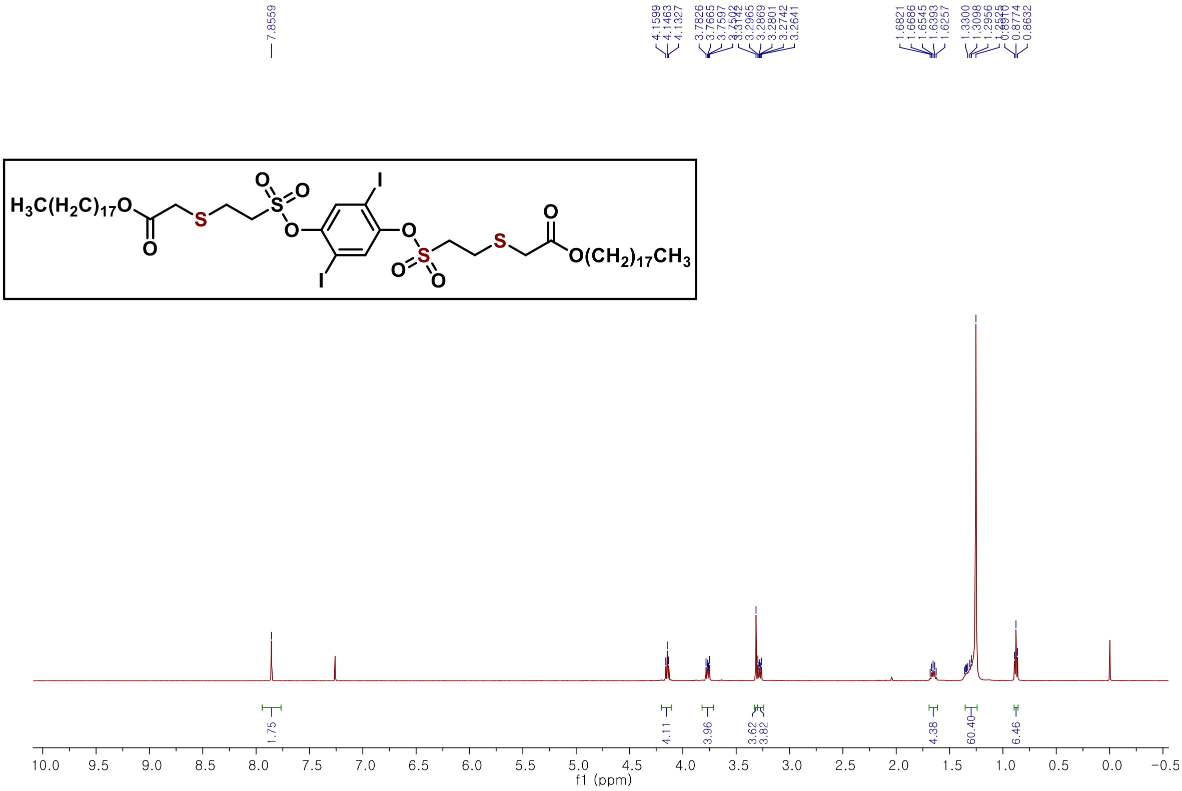

$^{13}C$ NMR (125 MHz, $CDCl_3$) spectrum of **9**

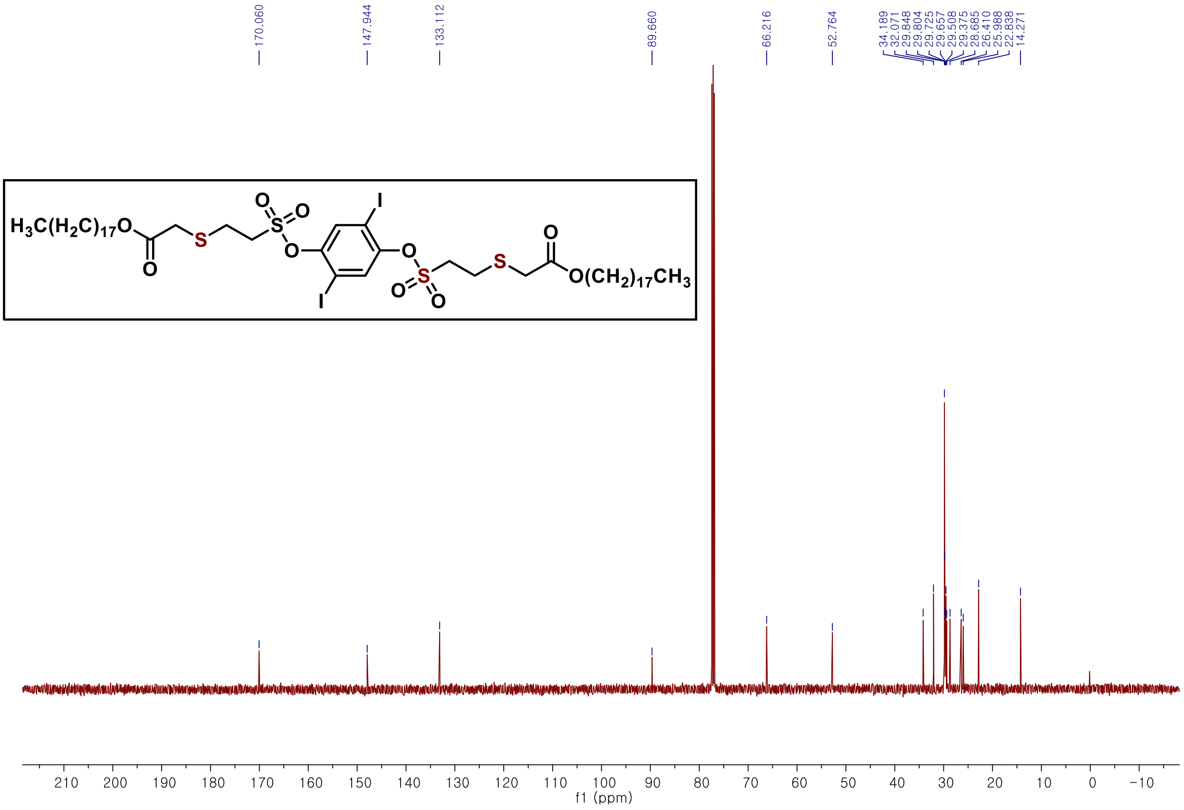

## $^{1}$H NMR (500 MHz, $CDCl_3$) spectrum of **10**

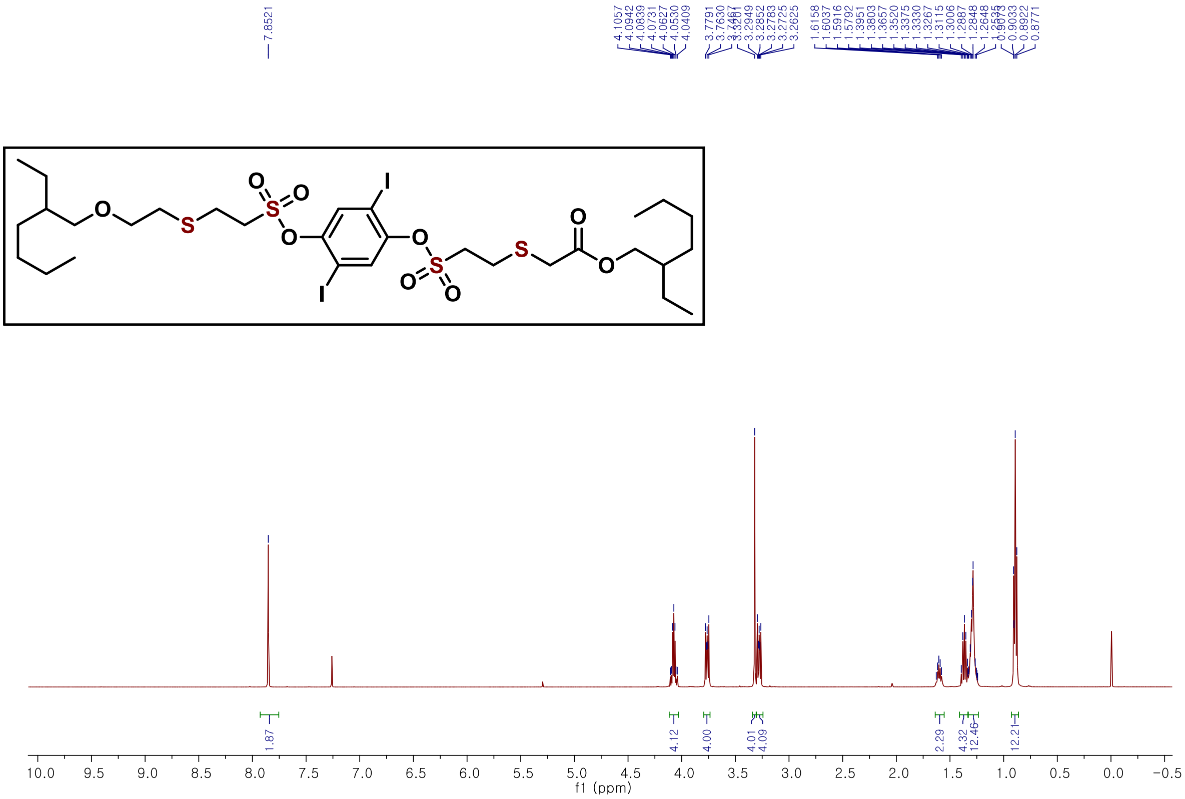


## $^{13}$C NMR (125 MHz, $CDCl_3$) spectrum of **10**

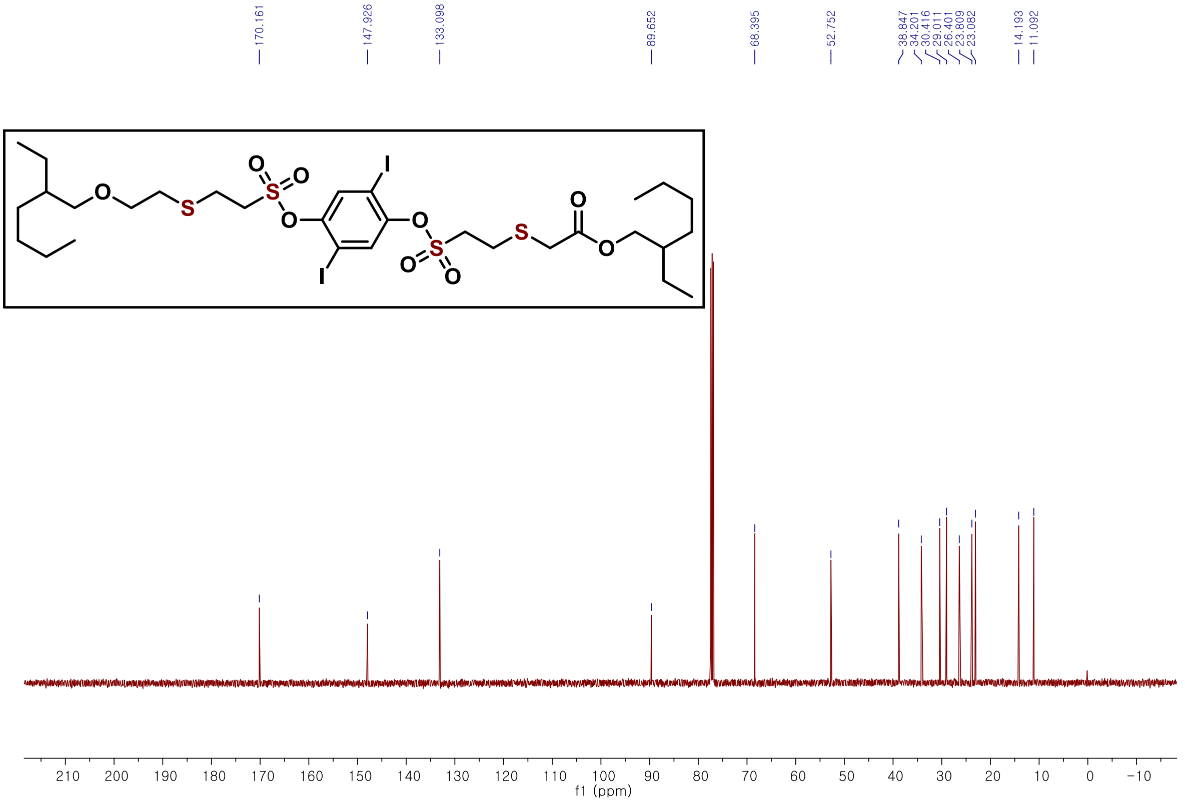

## $^{1}H$ NMR (500 MHz, $CDCl_3$) spectrum of **11**

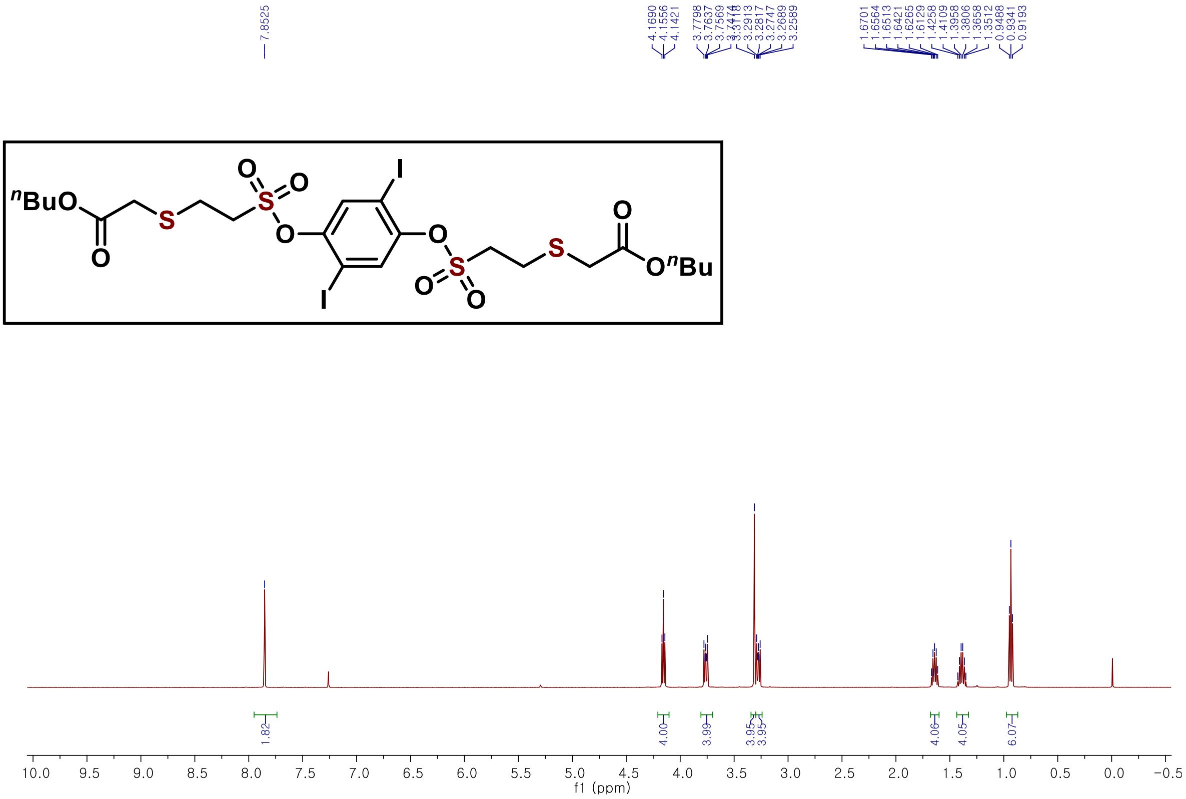


## $^{13}C$ NMR (125 MHz, $CDCl_3$) spectrum of **11**

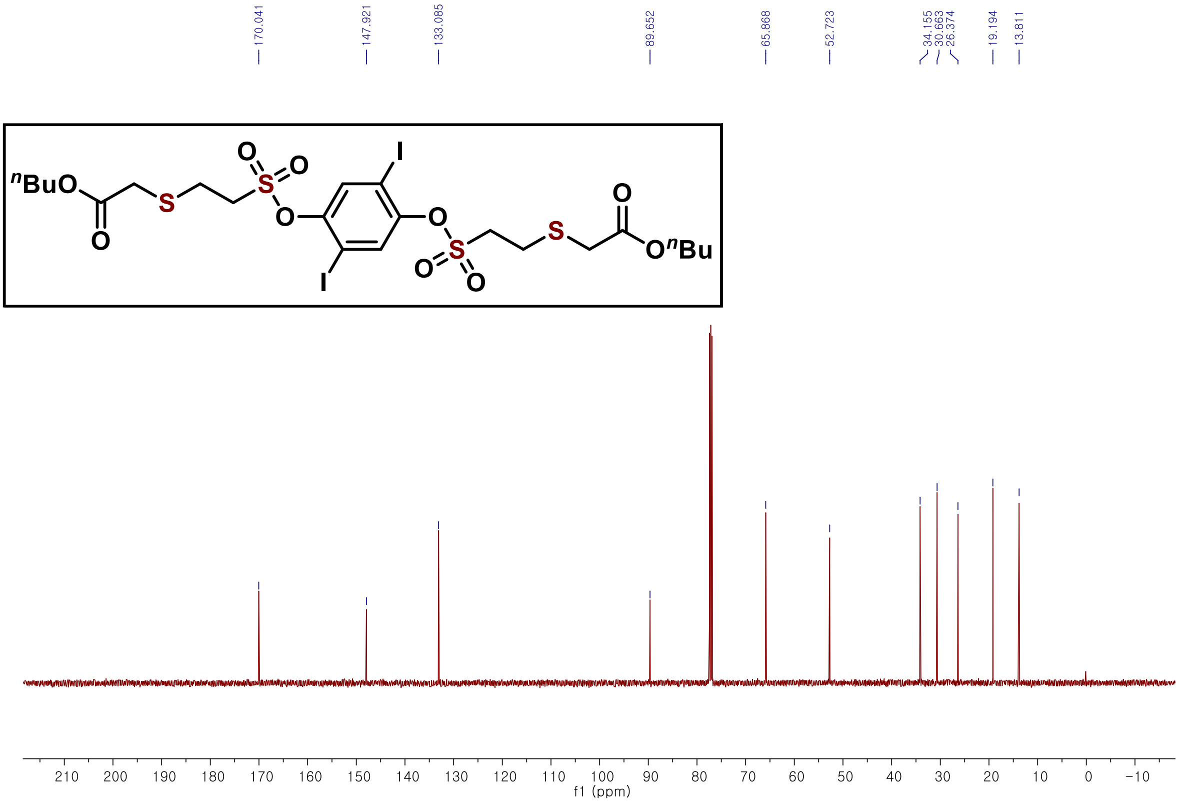

## $^{1}$H NMR (500 MHz, CDCl$_3$) spectrum of **12**

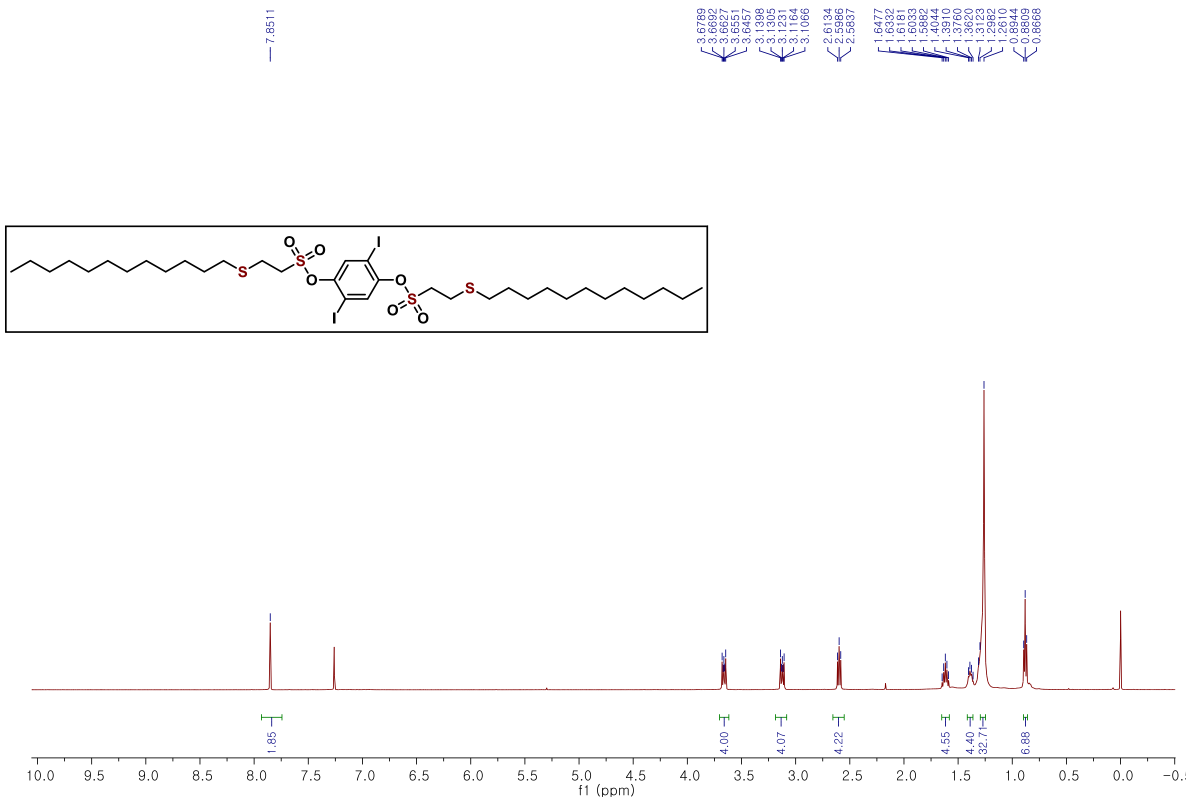


## $^{13}$C NMR (125 MHz, CDCl$_3$) spectrum of **12**

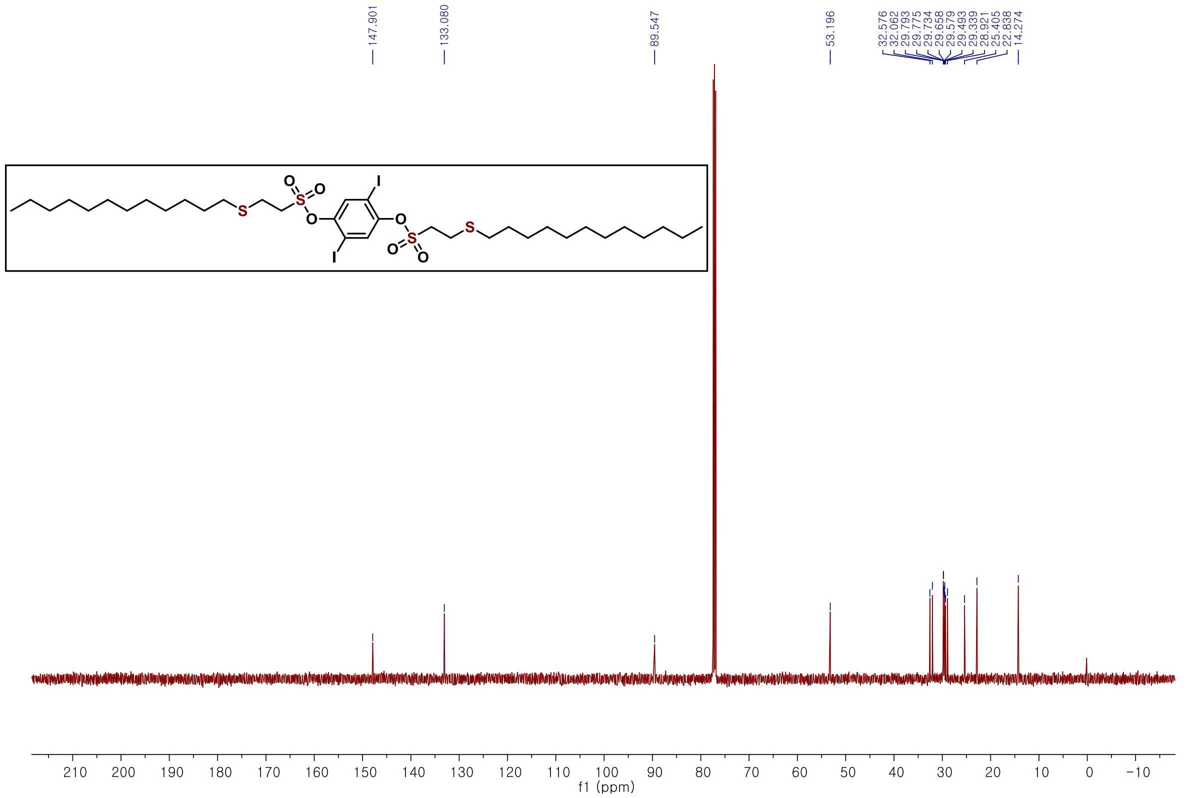

## $^{1}$H NMR (500 MHz, $CDCl_3$) spectrum of **13**

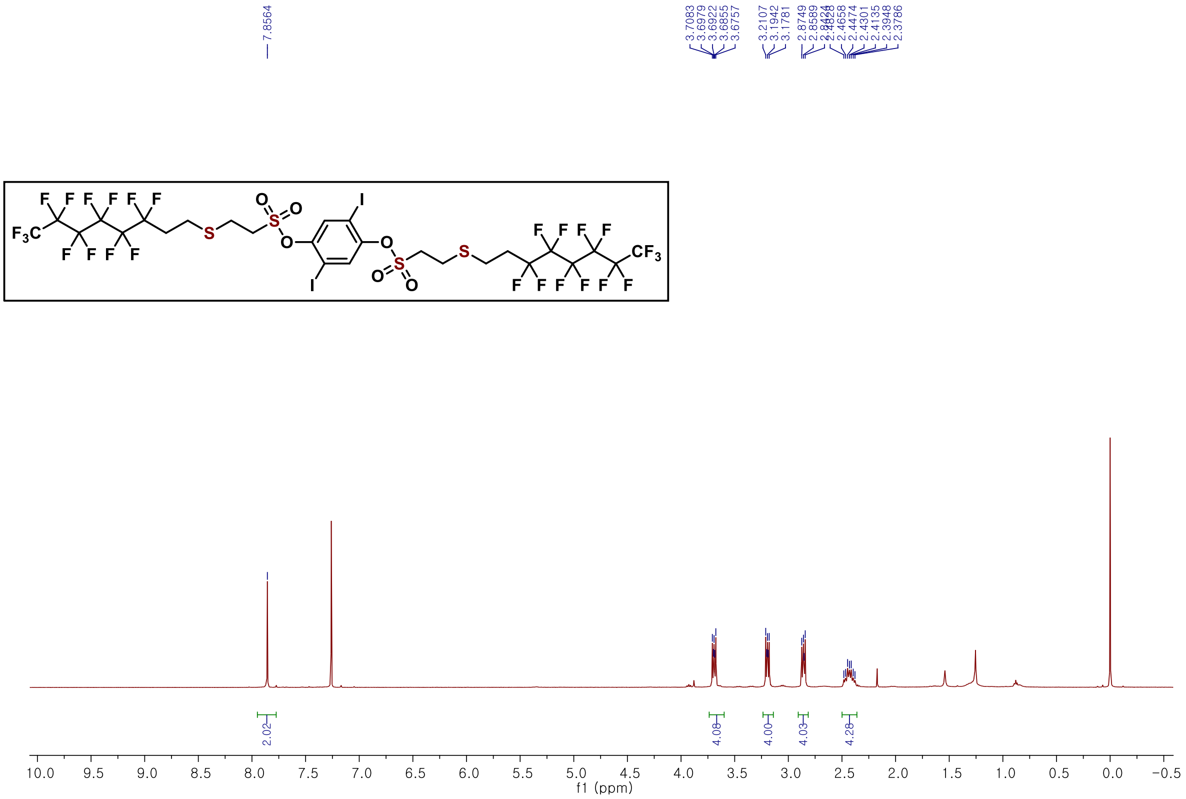


## $^{13}$C NMR (125 MHz, $CDCl_3$) spectrum of **13**

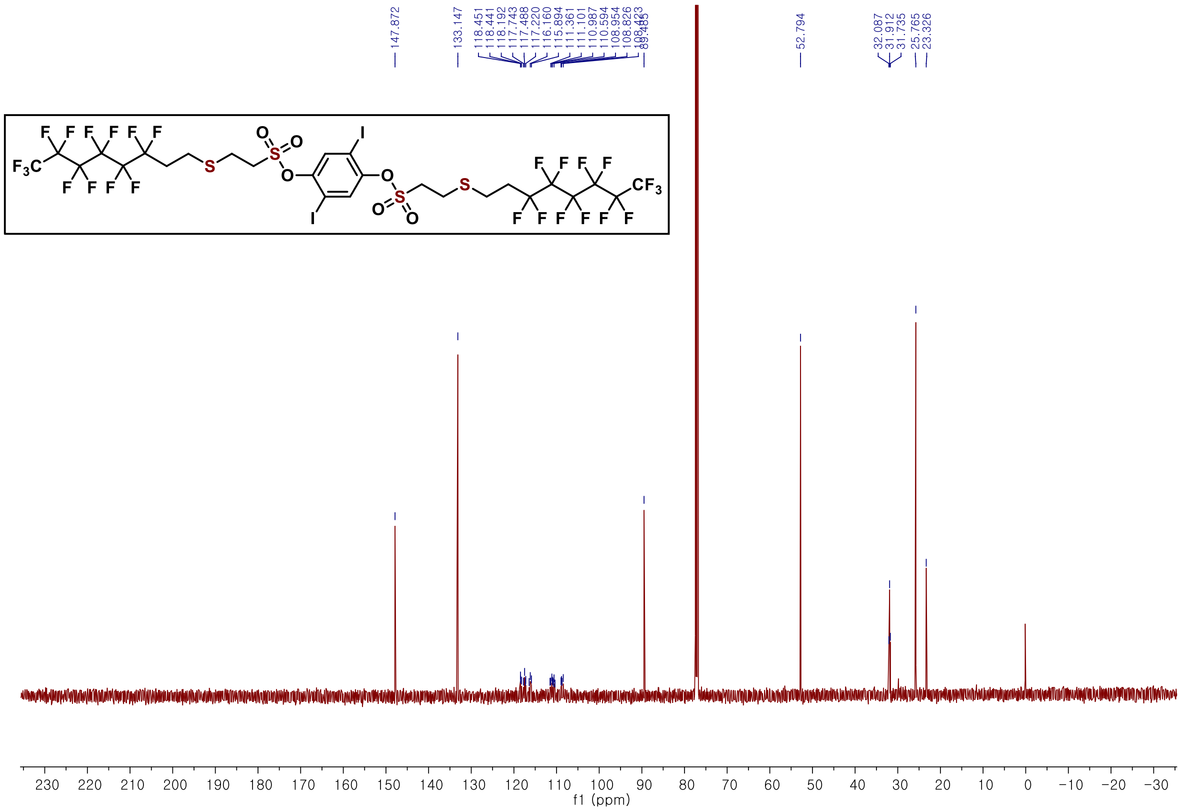

## $^{1}$H NMR (500 MHz, $CDCl_3$) spectrum of **14**

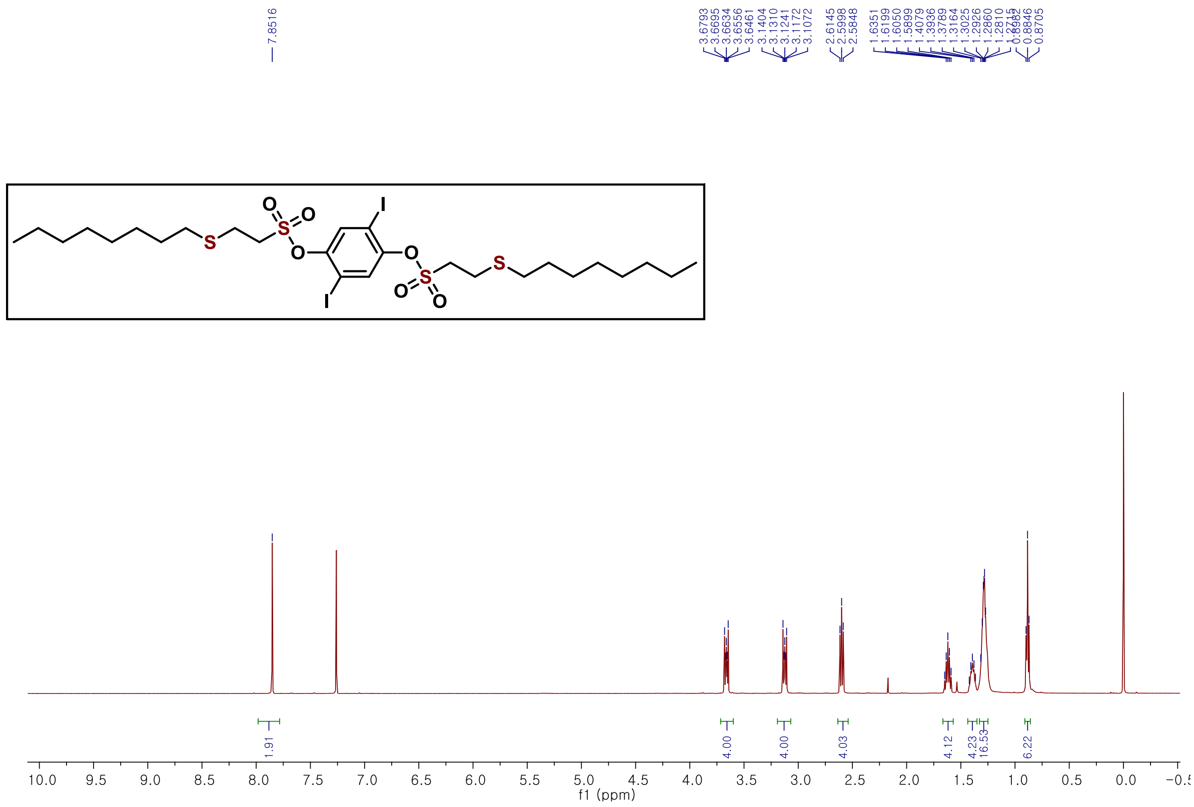


## $^{13}$C NMR (125 MHz, $CDCl_3$) spectrum of **14**

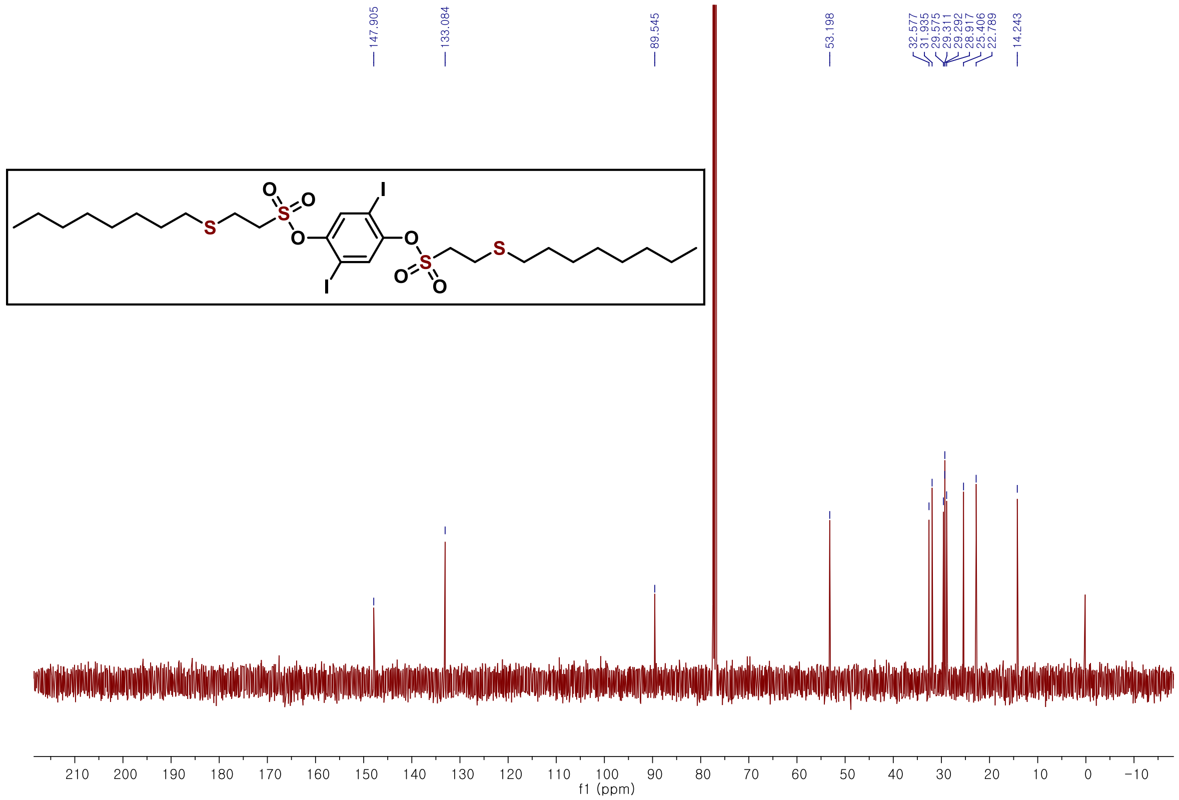

## $^1$H NMR (500 MHz, $CDCl_3$) spectrum of **SP-1**

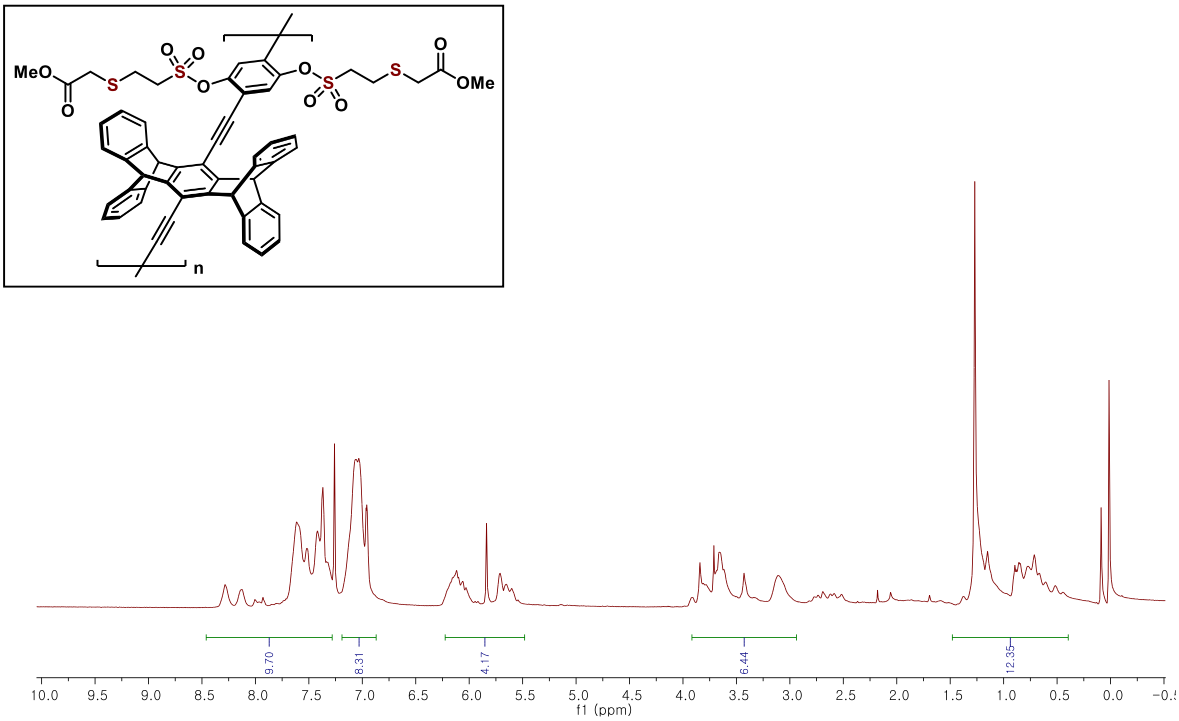


## $^1$H NMR (500 MHz, $CDCl_3$) spectrum of **SP-2**

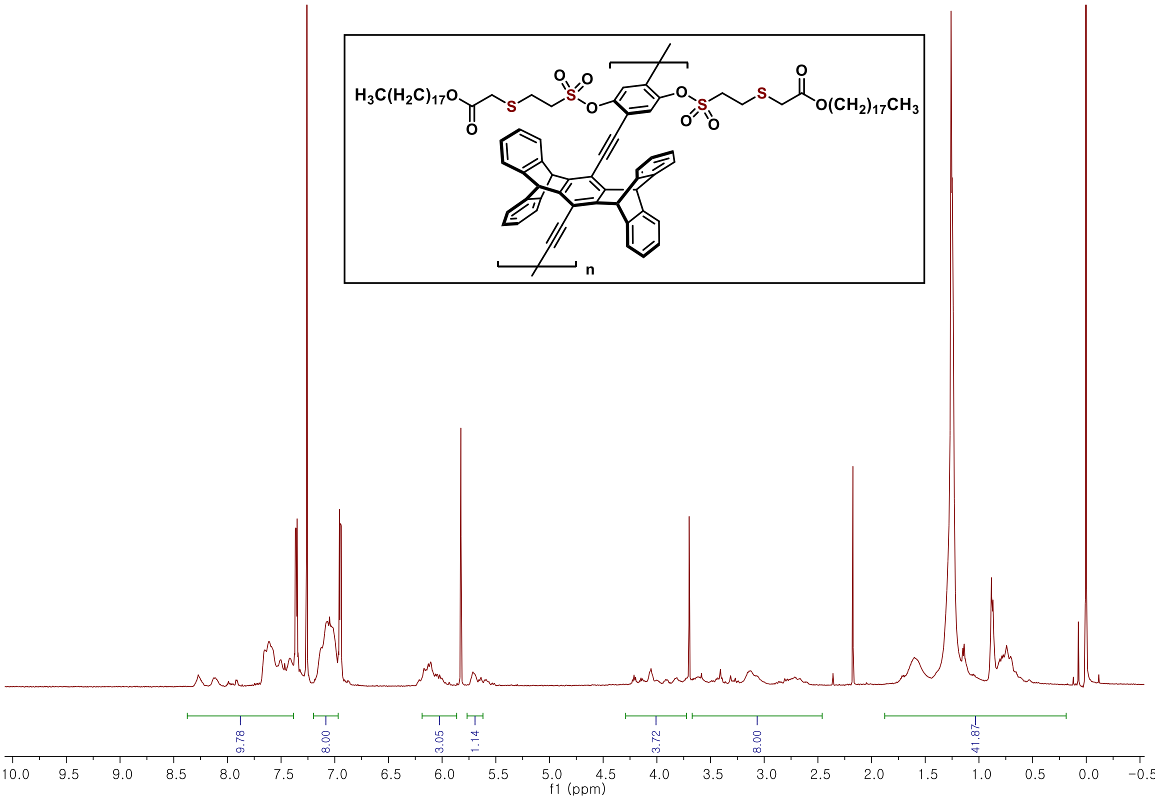

$^{1}$H NMR (500 MHz, $CDCl_3$) spectrum of **SP-3**

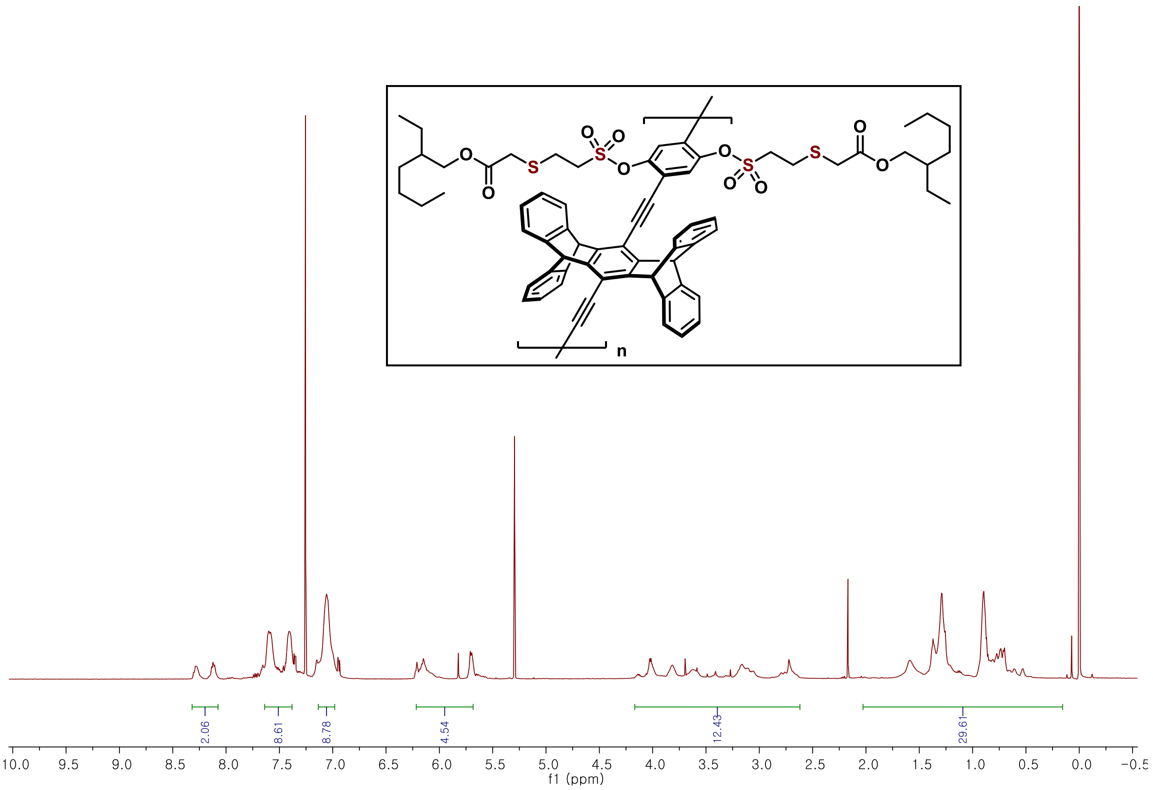


$^{1}$H NMR (500 MHz, $CDCl_3$) spectrum of **SP-4**

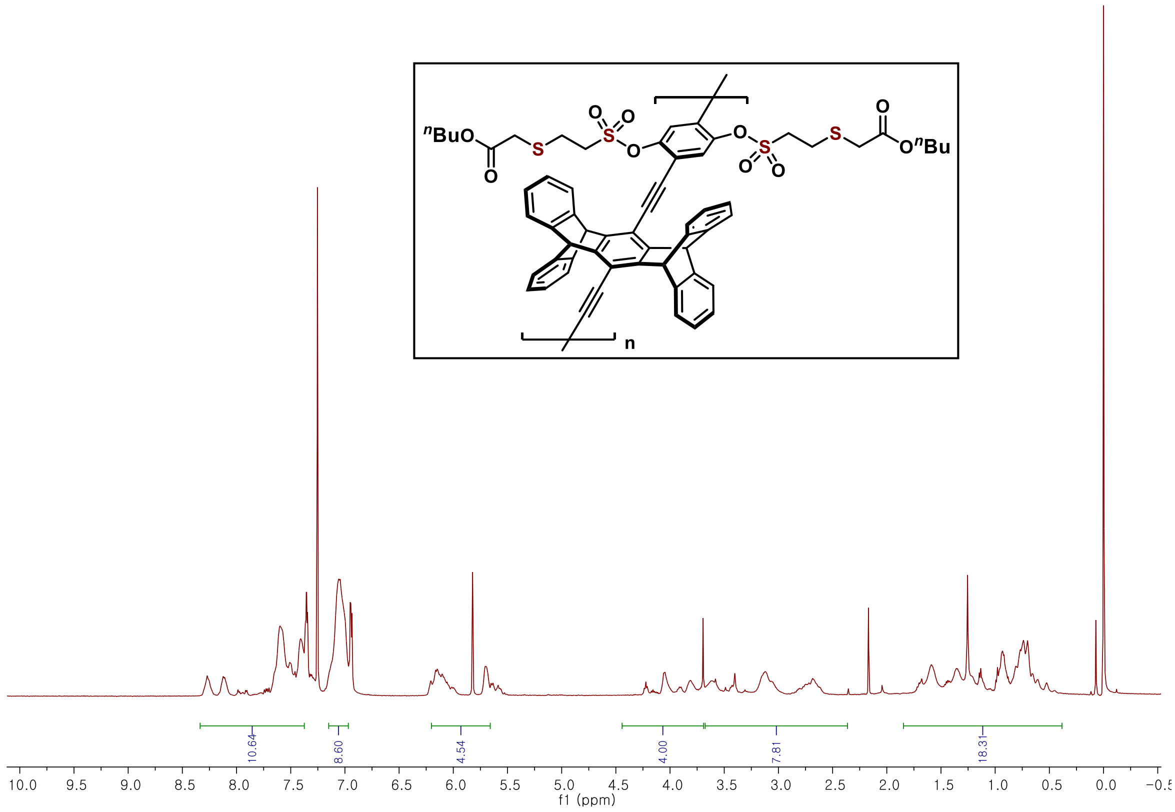

$^{1}H$ NMR (500 MHz, $CDCl_3$) spectrum of **SP-5**

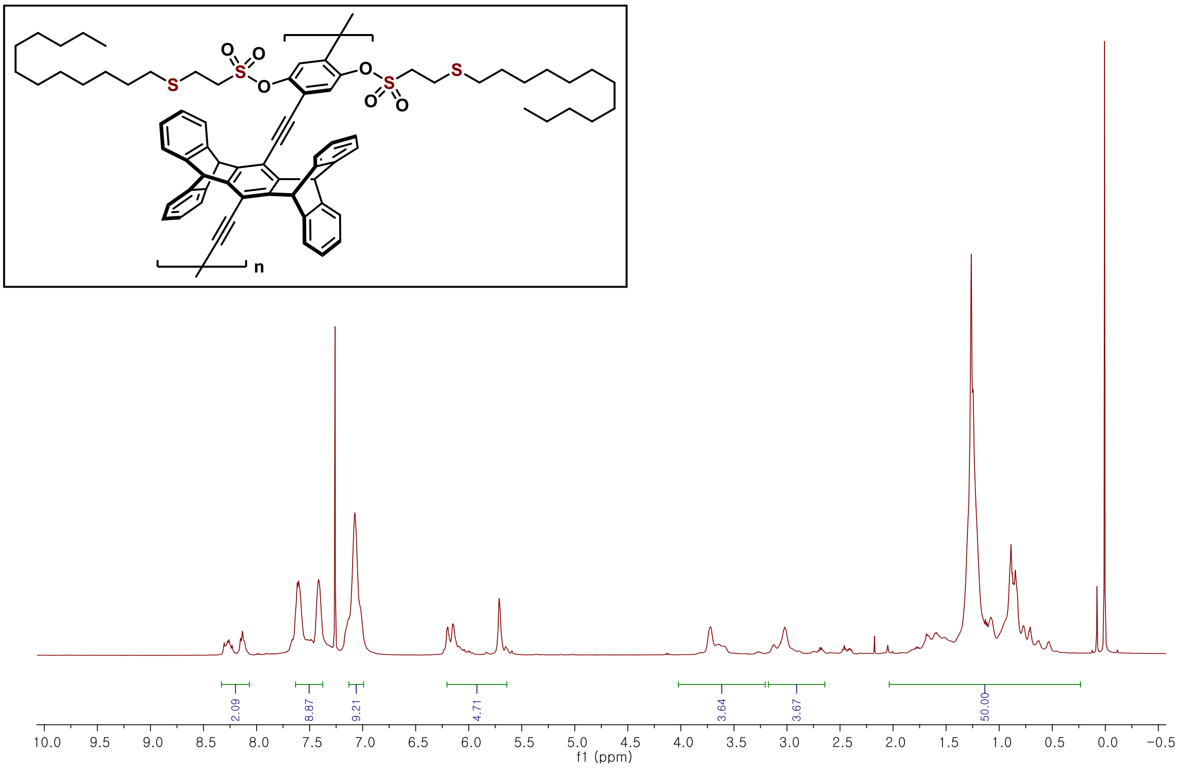


$^{1}H$ NMR (500 MHz, $CDCl_3$) spectrum of **SP-6**

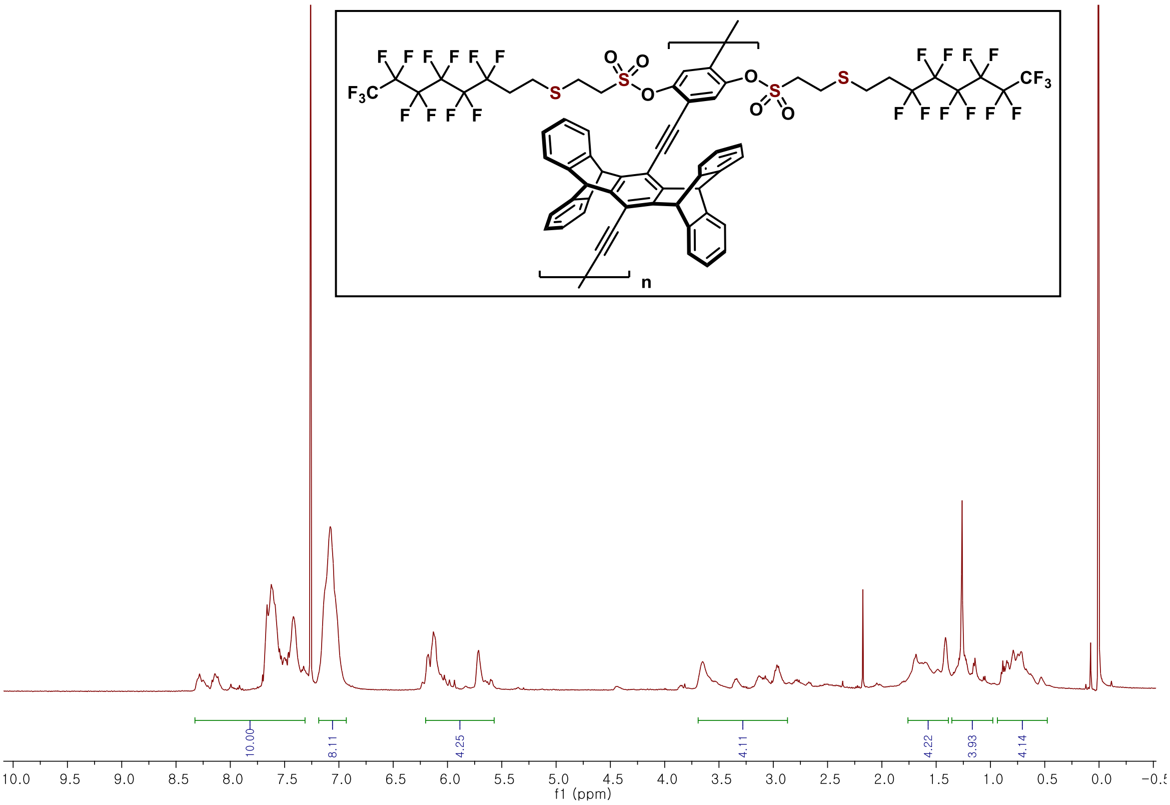

$^1$H NMR (500 MHz, CDCl$_3$) spectrum of **SP-7**

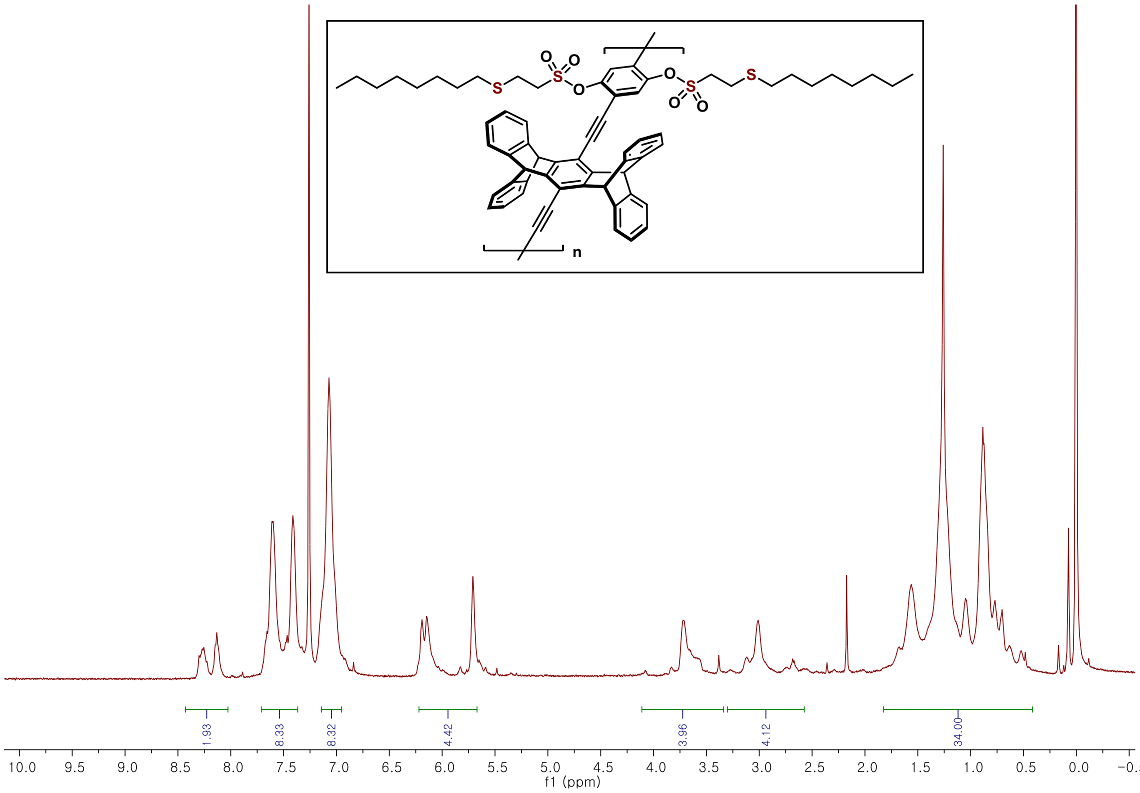

## 10. HR-MS data

HRMS spectrum of **8**

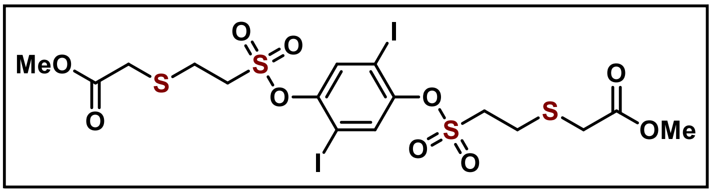


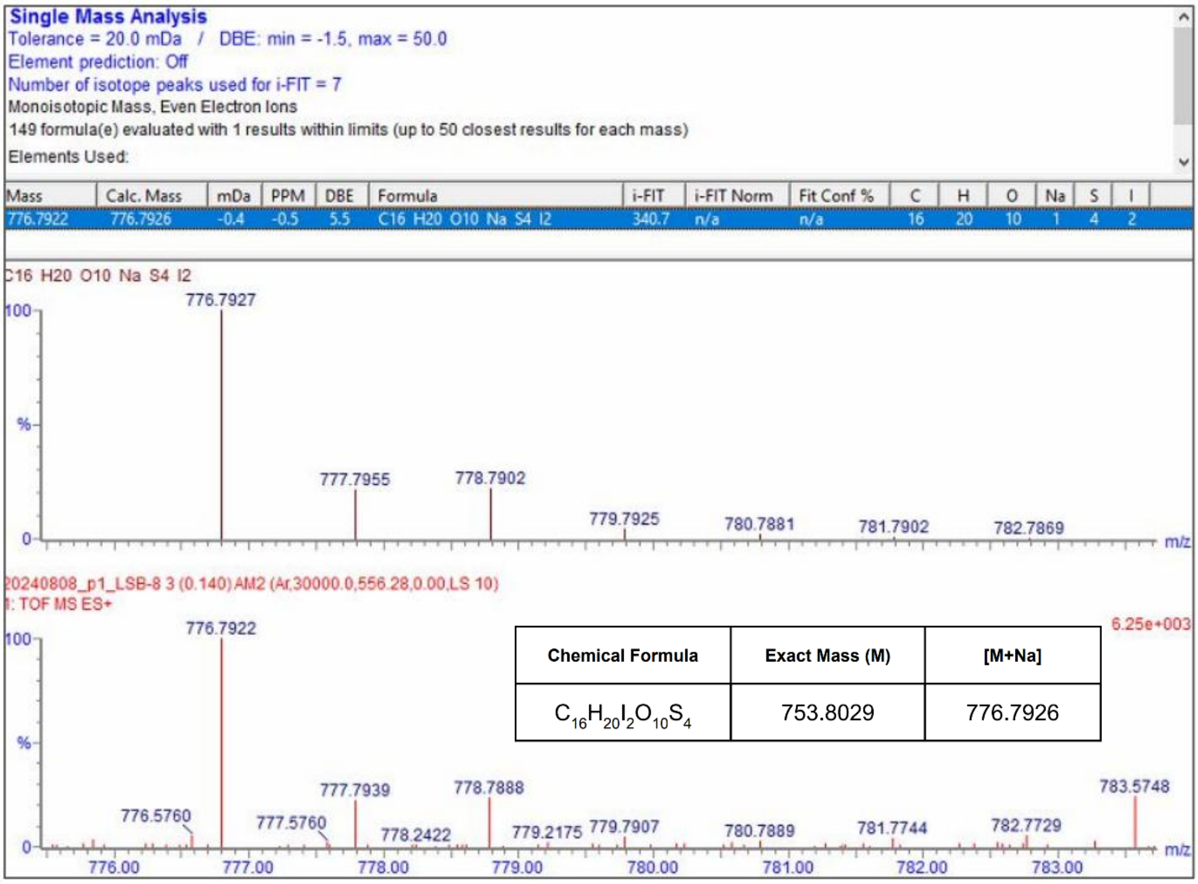

HRMS spectrum of **9**

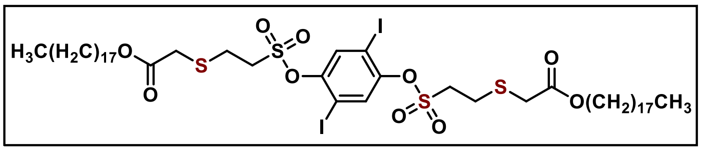


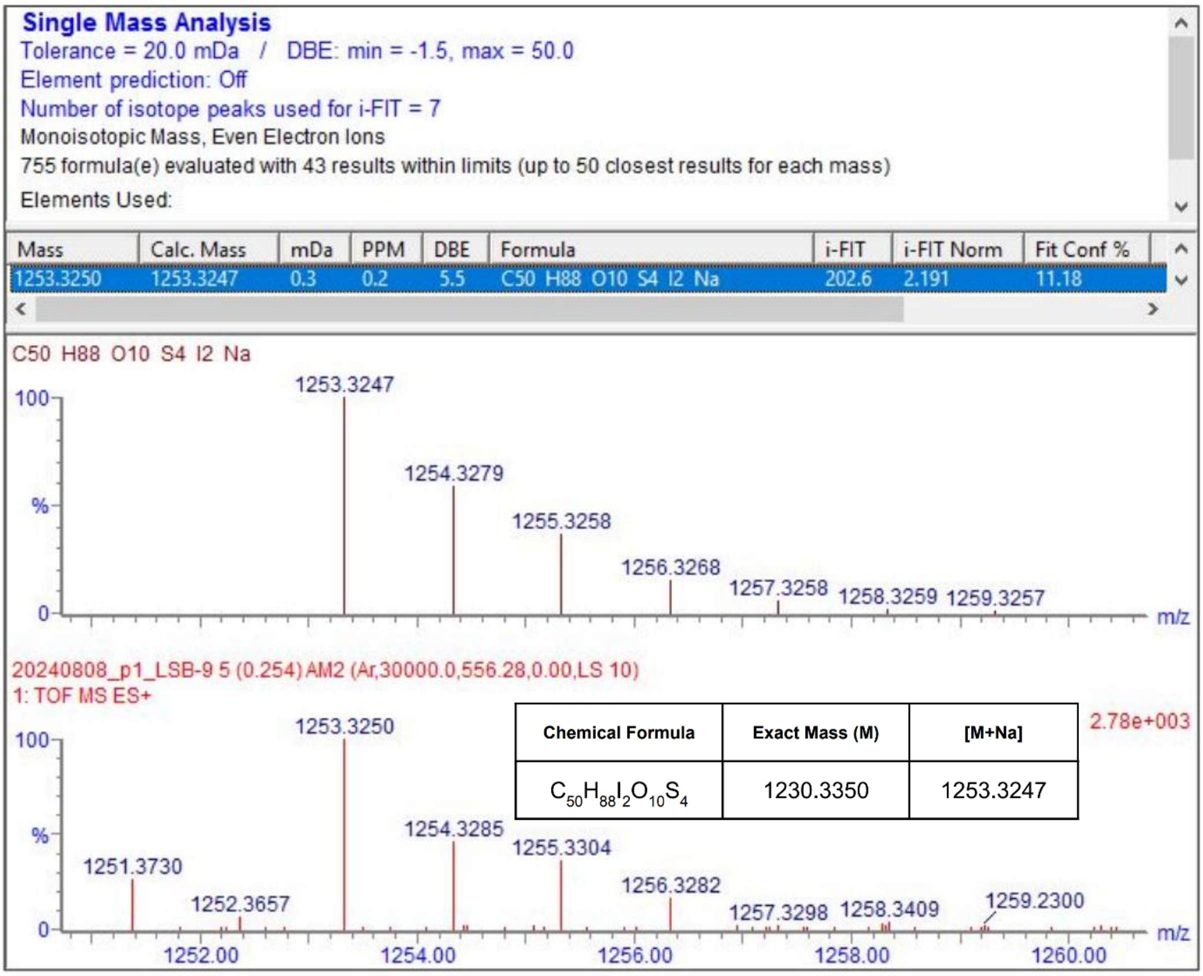


| Chemical Formula | Exact Mass (M) | [M+Na] |
|---|---|---|
| $C_{50}H_{88}I_2O_{10}S_4$ | 1230.3350 | 1253.3247 |

HRMS spectrum of **10**

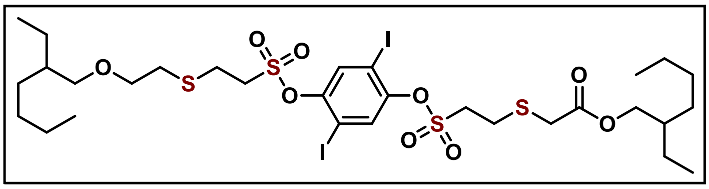


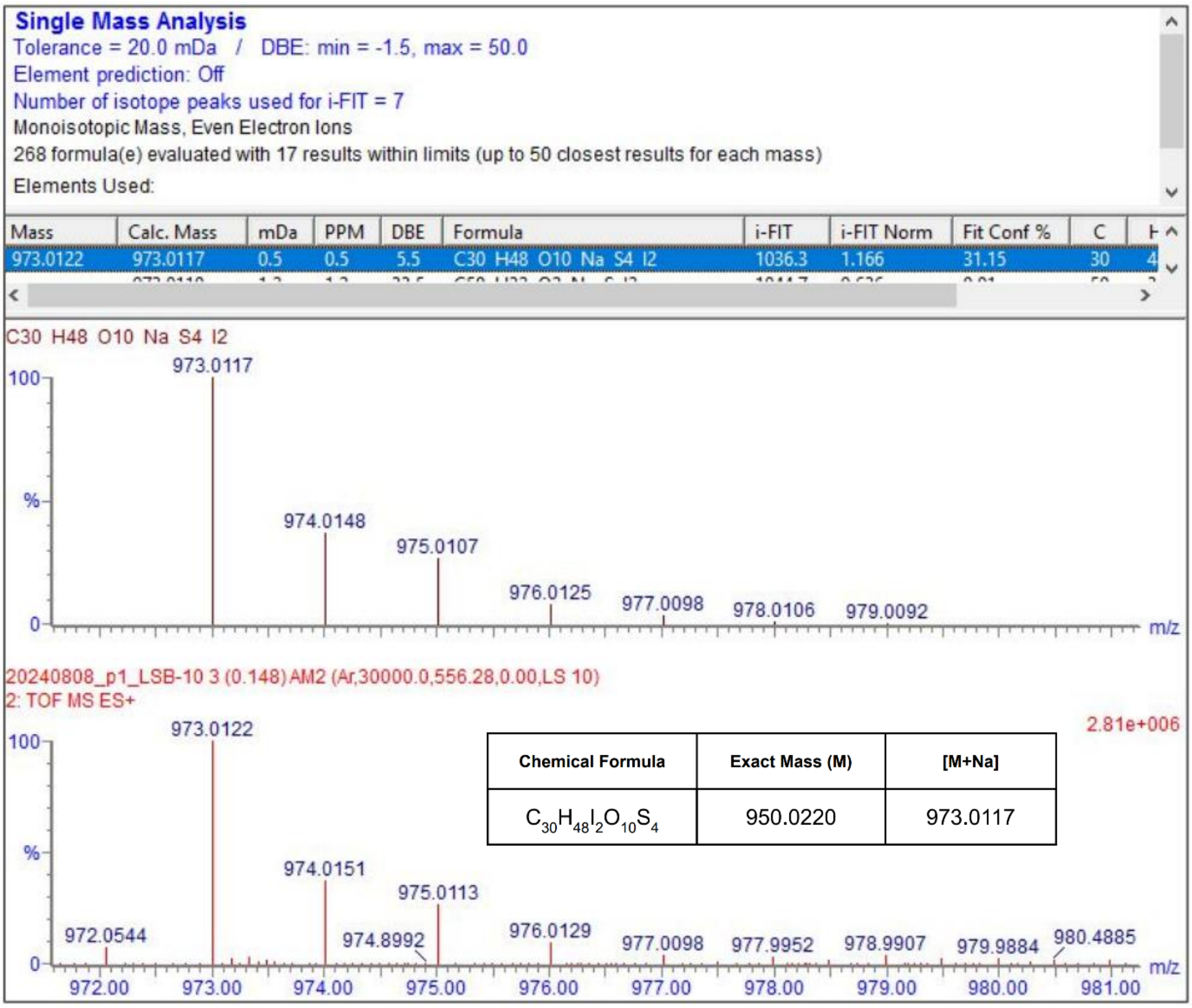

HRMS spectrum of **11**

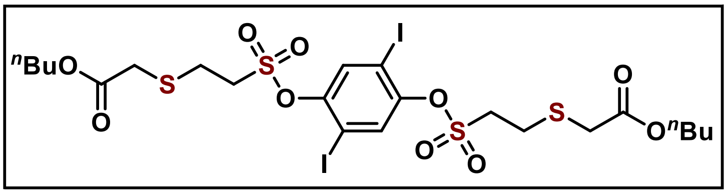

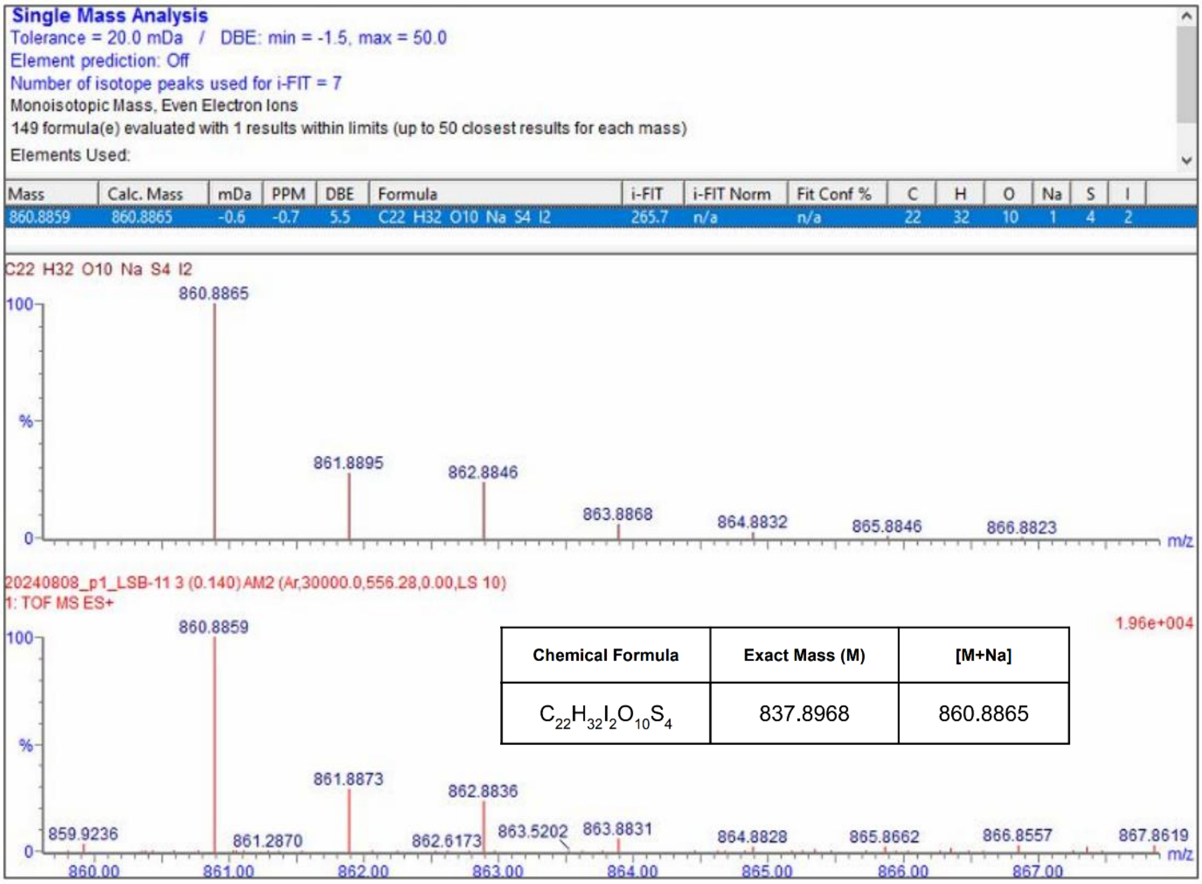


| Chemical Formula | Exact Mass (M) | [M+Na] |
|---|---|---|
| $C_{22}H_{32}I_2O_{10}S_4$ | 837.8968 | 860.8865 |

HRMS spectrum of **12**

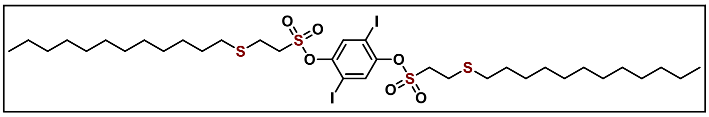

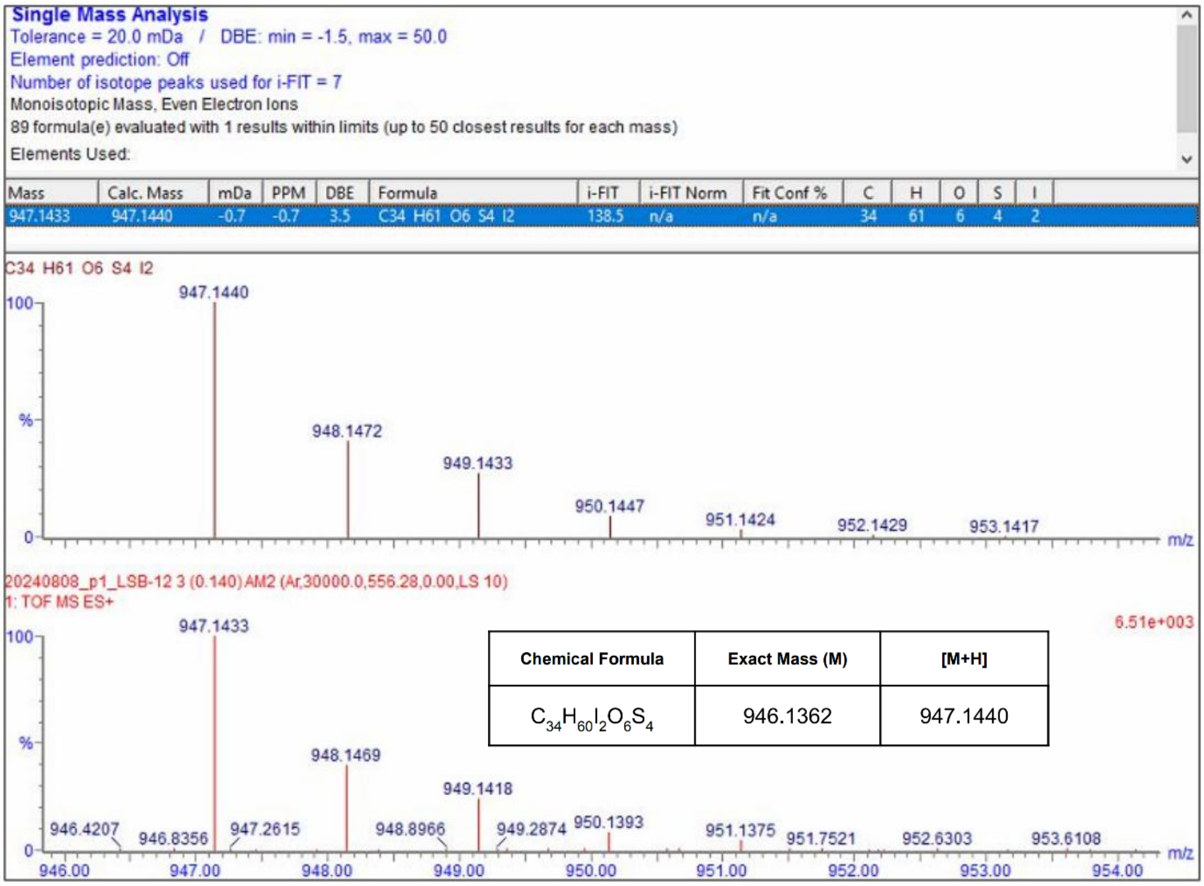

HRMS spectrum of **13**

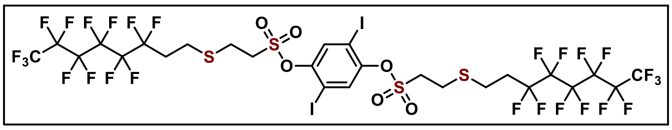

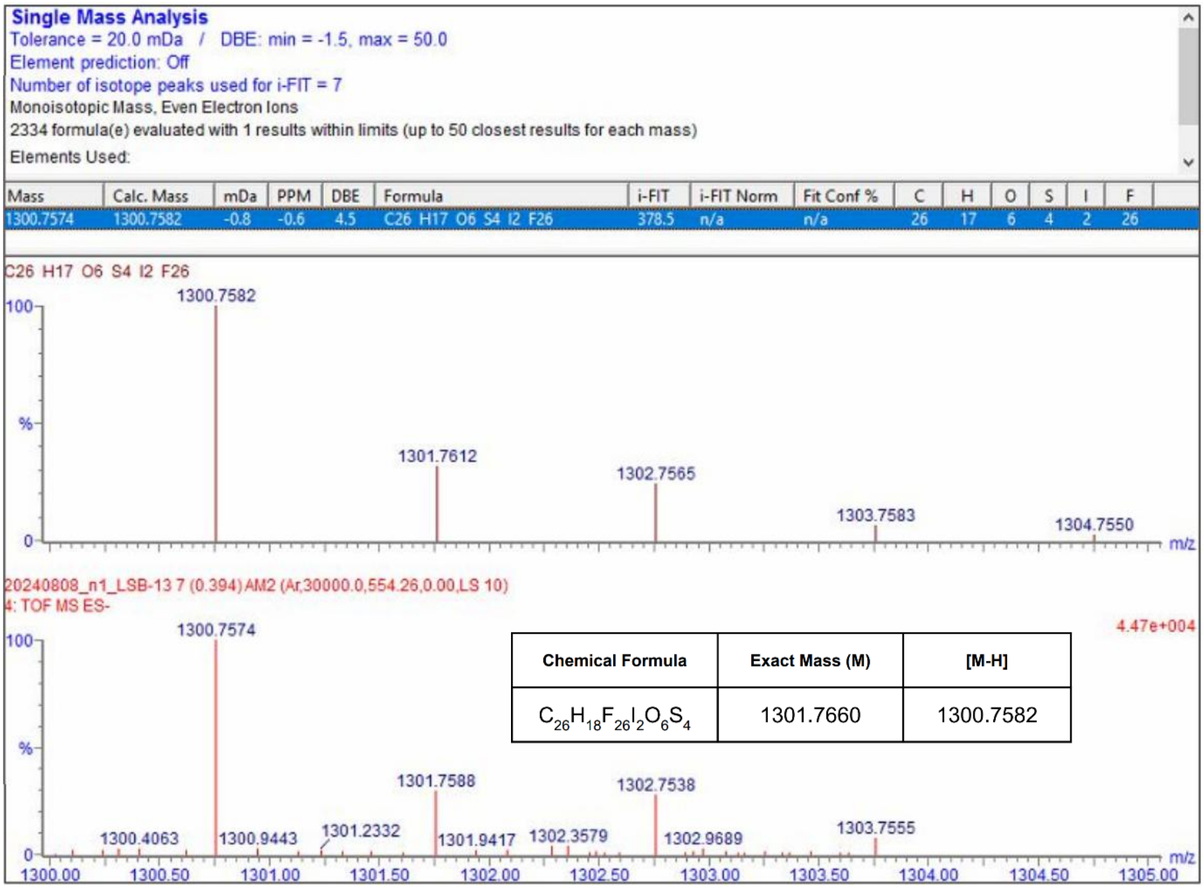


| Chemical Formula | Exact Mass (M) | [M-H] |
|---|---|---|
| $C_{26}H_{18}F_{26}I_2O_6S_4$ | 1301.7660 | 1300.7582 |

HRMS spectrum of **14**

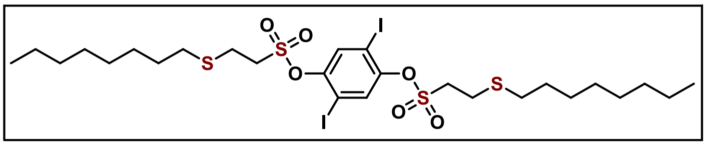


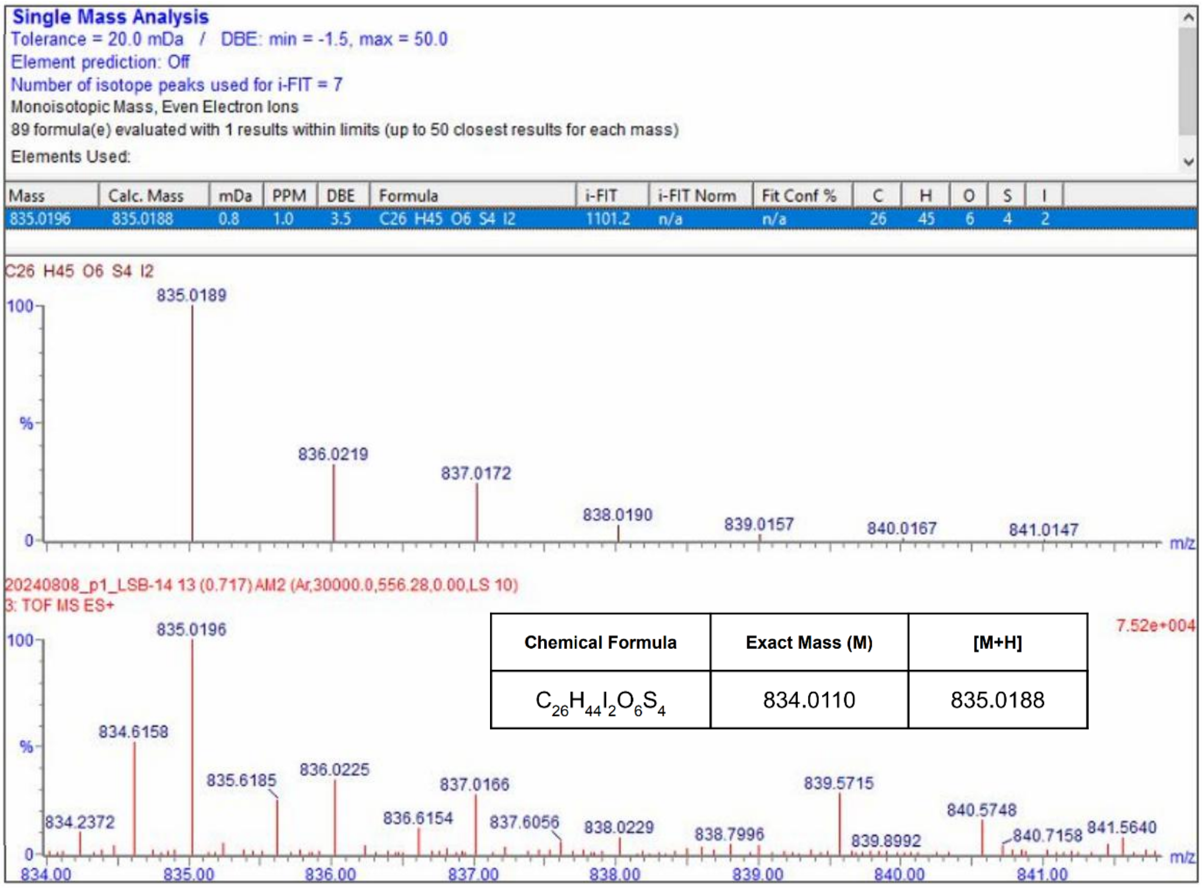

## 11. Reference


[1] S. A. Boer, P.-X. Wang, M. J. MacLachlan, N. G. White, Open Pentiptycene Networks Assembled through Charge-Assisted Hydrogen Bonds**,** *Cryst. Growth Des.* **2019**, *19*, 4829–4835.

[2] S. Ø. Scottwell, J. E. Barnsley, C. J. McAdam, K. C. Gordon, J. D. Crowley, A ferrocene based switchable molecular folding ruler**,** *Chem. Commun.* **2017**, *53*, 7628–7631.

[3] L. Liu, H. Zhiwei, W. Chunlin, C. Xuegang, L. Xianyi, L. Jie, Zn(II)-containing metallo-supramolecular polymers: synthesis, photophysical and electrochemical properties, J. *Macromol. Sci., Part A.* **2019**, *56*, 918–925.

[4] S. Saem, D. Fong, A. Adronov, J. Moran-Mirabal, Stretchable and Resilient Conductive Films on Polydimethylsiloxane from Reactive Polymer-Single-Walled Carbon Nanotube Complexes for Wearable Electronics, *ACS Appl. Nano Mater.* **2019**, *2*, 4968–4973.

[5] J.-S. Yang, T. M. Swager, Fluorescent Porous Polymer Films as TNT Chemosensors: Electronic and Structural Effects, *J. Am. Chem. Soc.* **1998**, *120*, 11864–11873.

[6] M. H. Gehlen, The centenary of the Stern-Volmer equation of fluorescence quenching: From the single line plot to the SV quenching map, *J. Photochem. Photobiol. C: Photochem. Rev.* **2020**, *42*, 100338.

[7] C. Ruckebusch, M. Sliwa, P. Pernot, A. de Juan, R. Tauler, Comprehensive data analysis of femtosecond transient absorption spectra: A review, *J. Photochem. Photobiol. C: Photochem. Rev.* **2012**, *13*, 1–27.

[8] S. E. Webber, The Role of Time-Dependent Measurements in Elucidating Static Versus Dynamic Quenching Processes**,** *Photochem. Photobiol.* **1997**, *65*, 33–38.

[9] X. Sun, Y. Wang, Y. Lei, Fluorescence based explosive detection: from mechanisms to sensory materials, *Chem. Soc. Rev.* **2015**, *44*, 8019–8061.
